# Unsupervised Phenotyping of Atrial Fibrillation Using Variational Autoencoders in a Critical Care Cohort: The ATLAS-AF Study

**DOI:** 10.1177/11795468261490000

**Published:** 2026-09-11

**Authors:** Samer G. Salman, Akhil Javvadi, Zane G. Salman, Sai Madhav Yedupati, Alessandra Pina, Prashanthan Sanders, Sainyam Galhotra, Ajay Tripuraneni

**Affiliations:** 1 School of Medicine, 3989 Baylor College of Medicine, Houston, TX, USA; 2 Alfred AI Inc, Austin, TX, USA; 3 Department of Neuroscience, College of Natural Sciences, 12330 The University of Texas at Austin, Austin, TX, USA; 4 College of Engineering and Applied Sciences, University of Cincinnati, Cincinnati, OH, USA; 5 Centre for Heart Rhythm Disorders, Adelaide University and Royal Adelaide Hospital, Adelaide, SA, Australia; 6 Department of Computer Science, Cornell University, Ithaca, NY, USA

**Keywords:** atrial fibrillation, phenotyping, variational autoencoder, unsupervised learning, risk stratification, survival analysis, precision medicine

## Abstract

**Objective:**

Atrial fibrillation (AF) is a heterogeneous disorder associated with substantial morbidity and mortality, yet management remains largely guided by traditional classifications and risk scores. We aimed to identify distinct AF phenotypes using variational autoencoder (VAE)-based unsupervised learning and to evaluate differences in mortality, healthcare utilization, and treatment patterns across them.

**Methods:**

Retrospective observational cohort study in MIMIC-IV v3.1, a single-center critical care database, restricted to the 2014 to 2016, 2017 to 2019, and 2020 to 2022 anchor year groups. In 13,967 patients with AF, a patient-level matrix of 35 demographic, comorbidity, medication, laboratory, AF characteristic, and utilization features was used to train a VAE for nonlinear dimensionality reduction, followed by K-means clustering at k = 6. Phenotypes were compared by Kaplan-Meier and Cox analysis of all-cause mortality, with Phenotype 4 (High AF Chronicity) as the reference because it had the longest reverse Kaplan-Meier follow-up, and by incremental discrimination beyond routinely available variables. Reproducibility was assessed by two-level bootstrap resampling against a permuted-data null.

**Results:**

Six phenotypes were identified (n = 1,906 to 2,925): Phenotype 1 (Cerebrovascular-Predominant), Phenotype 2 (Hypoalbuminemic-Anemic), Phenotype 3 (Low Comorbidity Burden), Phenotype 4 (High AF Chronicity), Phenotype 5 (Warfarin-Predominant Anticoagulation), and Phenotype 6 (Renal Dysfunction). All-cause mortality differed across phenotypes (log-rank p<0.001), with 14 of 15 pairwise comparisons significant after Bonferroni correction. Observed-death proportions ranged from 11.1% to 48.1%, but accrued over markedly unequal observation windows (reverse Kaplan-Meier median follow-up 5.3 to 854.9 days) and are not comparable risks. Phenotype membership remained associated with mortality after adjustment for age and sex, the adjustment set that minimizes overlap with the phenotype-defining features. Adding phenotype to a model of age, sex, CHA_2_DS_2_-VASc, and three routine laboratory values raised the concordance index from 0.683 to 0.733, although a conventional model containing utilization variables and no phenotype term reached 0.745. Thirty-day readmission ranged from 13.0% to 42.1% and intensive care admission from 33.6% to 77.8%; because utilization variables also helped define the clusters, these are descriptive characteristics of the phenotypes rather than independent outcomes, and rebuilding the phenotypes without those variables halved the intensive care spread to 25.1 percentage points.

**Conclusion:**

VAE-based unsupervised learning identified six AF phenotypes with substantial differences in mortality, healthcare utilization, and treatment patterns, highlighting clinically meaningful heterogeneity within AF. Cluster separation metrics were modest and individual phenotype assignment was only moderately reproducible when the model was refit end to end (Adjusted Rand Index 0.406), indicating that phenotypes lie along overlapping continua rather than representing discrete entities, although a between-phenotype mortality gradient was present in all 100 end-to-end resamples (minimum range 18.8 percentage points). Temporal validation did not meet the pre-specified ±5% threshold for phenotype distributions (maximum difference 9.0%). These findings are hypothesis-generating and support future prospective evaluation of phenotype-guided risk stratification, with the longer-term goal of informing precision cardiovascular medicine.

## Introduction

Atrial fibrillation (AF) is the most common sustained cardiac arrhythmia. It affects more than 33 million people worldwide and contributes substantially to cardiovascular morbidity and mortality.^1^ Even with that clinical burden, AF management has largely followed a one-size-fits-all model. Treatment decisions are still guided mainly by duration-based categories such as paroxysmal, persistent, long standing persistent, and permanent AF, along with risk scores like CHA_2_DS_2_-VASc and HAS-BLED.^1^ But growing evidence shows that AF is not a single uniform disease. It is a highly heterogeneous condition, with different underlying mechanisms, comorbidity patterns, and clinical courses that are not well captured by traditional classification schemes.^2,3^ That heterogeneity suggests a clear problem: when we treat all AF patients as though they are broadly the same, we may miss opportunities for more precise and effective care.^4^

Recent advances in machine learning have begun to shift the field toward unsupervised phenotype discovery in AF. Several studies have used clustering methods to identify clinically distinct AF subgroups with different risk profiles and outcomes.^5-9^ Across these studies, a consistent pattern has emerged: data-driven phenotypes often stratify patients more effectively than conventional risk scores alone, and they reveal subgroups with different comorbidities, treatment responses, and prognoses. In large registries such as ORBIT-AF, GARFIELD-AF, and CABANA, for example, cluster analyses have identified phenotypes that range from younger, lower-risk patients to older, multimorbid groups with substantially higher mortality.^5,6,8^ Still, most of this work has relied on traditional clustering approaches such as K-means, K-prototype, or hierarchical clustering applied either directly to clinical features or after linear dimensionality reduction with principal component analysis. These approaches often require substantial parameter tuning and do not learn representations from the underlying data distribution.

To our knowledge, no prior study has used variational autoencoders (VAEs) for unsupervised phenotype discovery in AF populations. VAEs offer a fundamentally different approach to dimensionality reduction and representation learning.^10-12^ Unlike linear methods, VAEs use deep neural networks to learn nonlinear, probabilistic latent representations of high-dimensional clinical data. That makes them well suited to capturing complex, non-additive relationships among clinical features that linear methods may miss.^12-15^ VAEs have already shown promise in cancer subtyping, disease phenotyping from multi-omics data, and the extraction of biologically meaningful latent spaces from high-dimensional biomedical datasets.^10,11,13,16^ In AF, one recent study used a VAE to extract ECG features before applying tree-based clustering, but that work focused on electrocardiographic signals rather than comprehensive clinical phenotyping, and the VAE functioned primarily as an ECG feature extractor rather than a method for integrating diverse clinical variables.^17^ The present study addresses that gap by applying VAE-based unsupervised learning to a comprehensive patient-level feature matrix that includes demographics, comorbidities, medications, laboratory values, and AF characteristics. Our goal was to discover clinically actionable phenotypes that differ in mortality, healthcare utilization, and treatment patterns.

We hypothesized that VAE-based dimensionality reduction, followed by clustering in the learned latent space, would identify distinct AF phenotypes with clinically meaningful differences in outcomes and treatment patterns. By using the VAE’s ability to learn nonlinear representations, we sought to uncover phenotypic structure that may remain hidden when traditional clustering methods are applied directly to raw clinical data or to linearly transformed features. Identifying such phenotypes could support phenotype-guided risk stratification and management strategies, and move AF care closer to a more personalized, precision-based model.

## Methods

### Study Design, Setting, and Period

This was a retrospective observational cohort study of patients with atrial fibrillation (AF) cared for at a single tertiary academic medical center, Beth Israel Deaconess Medical Center (Boston, Massachusetts, United States), and captured in the Medical Information Mart for Intensive Care IV (MIMIC-IV) version 3.1 database. No patients were prospectively enrolled and no intervention was assigned; all data were generated during routine clinical care and were extracted retrospectively.

MIMIC-IV shifts every patient’s dates by a random offset for de-identification and reports the true calendar period of a patient’s care only as a three-year anchor year group, so the study period is specified by anchor year group rather than by exact admission dates. Eligible patients were those in the 2014 to 2016, 2017 to 2019, and 2020 to 2022 anchor year groups, so the cohort covers hospital admissions occurring between 2014 and 2022, a nine-year period. Patients in the 2008 to 2010 and 2011 to 2013 anchor year groups were excluded because International Classification of Diseases, Tenth Revision (ICD-10) coding was not consistently available for them.

### Data Source

MIMIC-IV version 3.1 is a publicly available, de-identified database curated from the electronic health record of Beth Israel Deaconess Medical Center and distributed through PhysioNet under a credentialed data use agreement. It contains hospital-wide administrative and clinical data, including admissions and transfers, International Classification of Diseases Ninth and Tenth Revision diagnosis and procedure codes, laboratory results, microbiology, prescriptions, and medication administration records, together with a separate module of intensive care unit charting. All variables used in this study were derived from the hospital module of the source tables rather than from a preprocessed derivative dataset. Records were linked across encounters by the de-identified patient identifier, so for each eligible patient all hospital encounters were retrieved, not only those carrying an AF diagnosis, to support longitudinal characterization.

### Inclusion and Exclusion Criteria

Eligibility was assessed at the patient level and applied in the order given below.

Inclusion criteria: (1) presence in MIMIC-IV version 3.1, which contains records only for patients aged 18 years or older at admission; (2) at least one hospital admission carrying an ICD-10 atrial fibrillation diagnosis code in any diagnosis position, where atrial fibrillation was defined by an explicit six-code list, namely I48.0 (paroxysmal), I48.11 (longstanding persistent), I48.19 (other persistent), I48.20 (chronic, unspecified), I48.21 (permanent), and I48.91 (unspecified); and (3) membership of the 2014 to 2016, 2017 to 2019, or 2020 to 2022 anchor year group.

**Figure 1.**
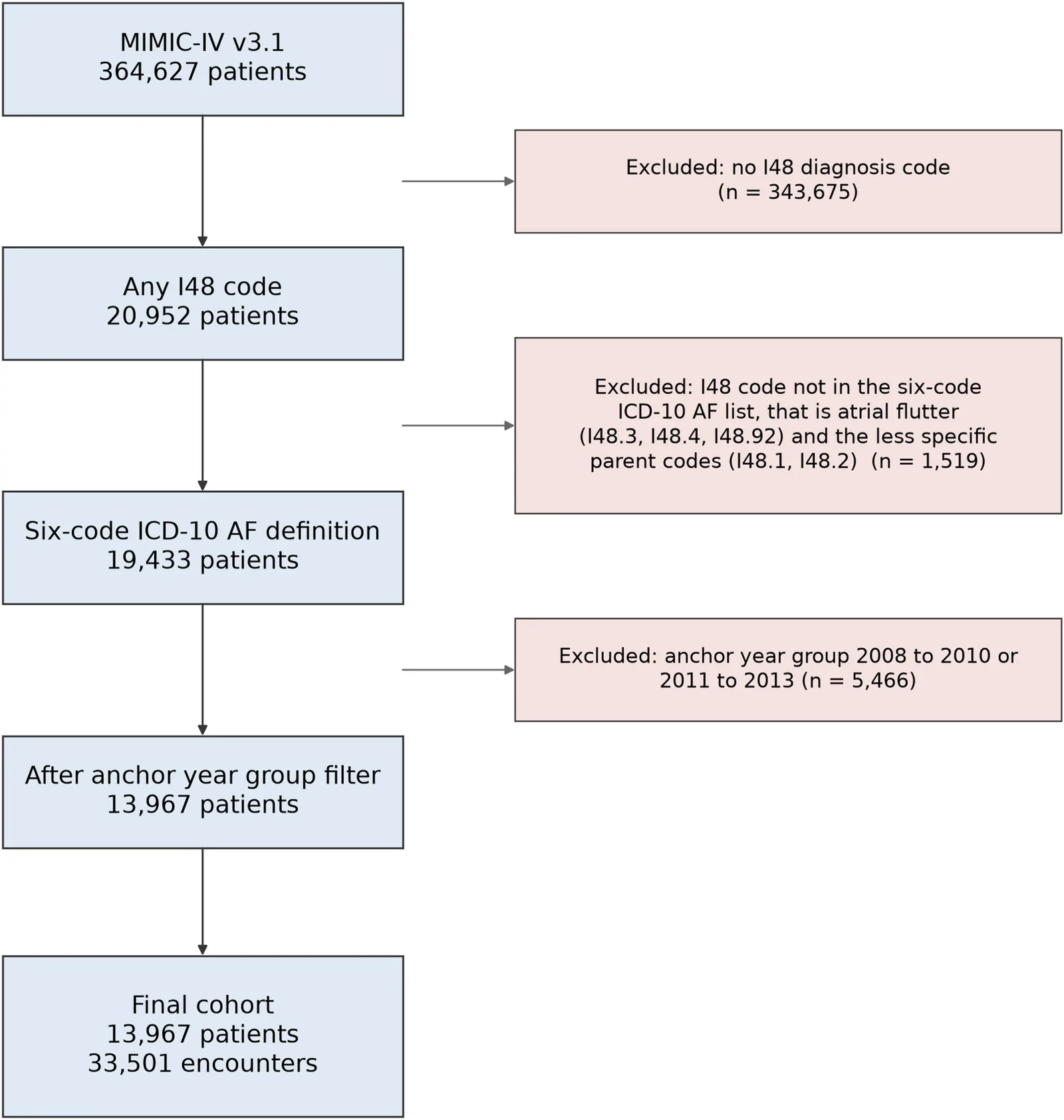
STROBE flow diagram of patient selection. Abbreviations: ICD-10 = International Classification of Diseases, Tenth Revision; MIMIC-IV = Medical Information Mart for Intensive Care IV

**Figure 2.**
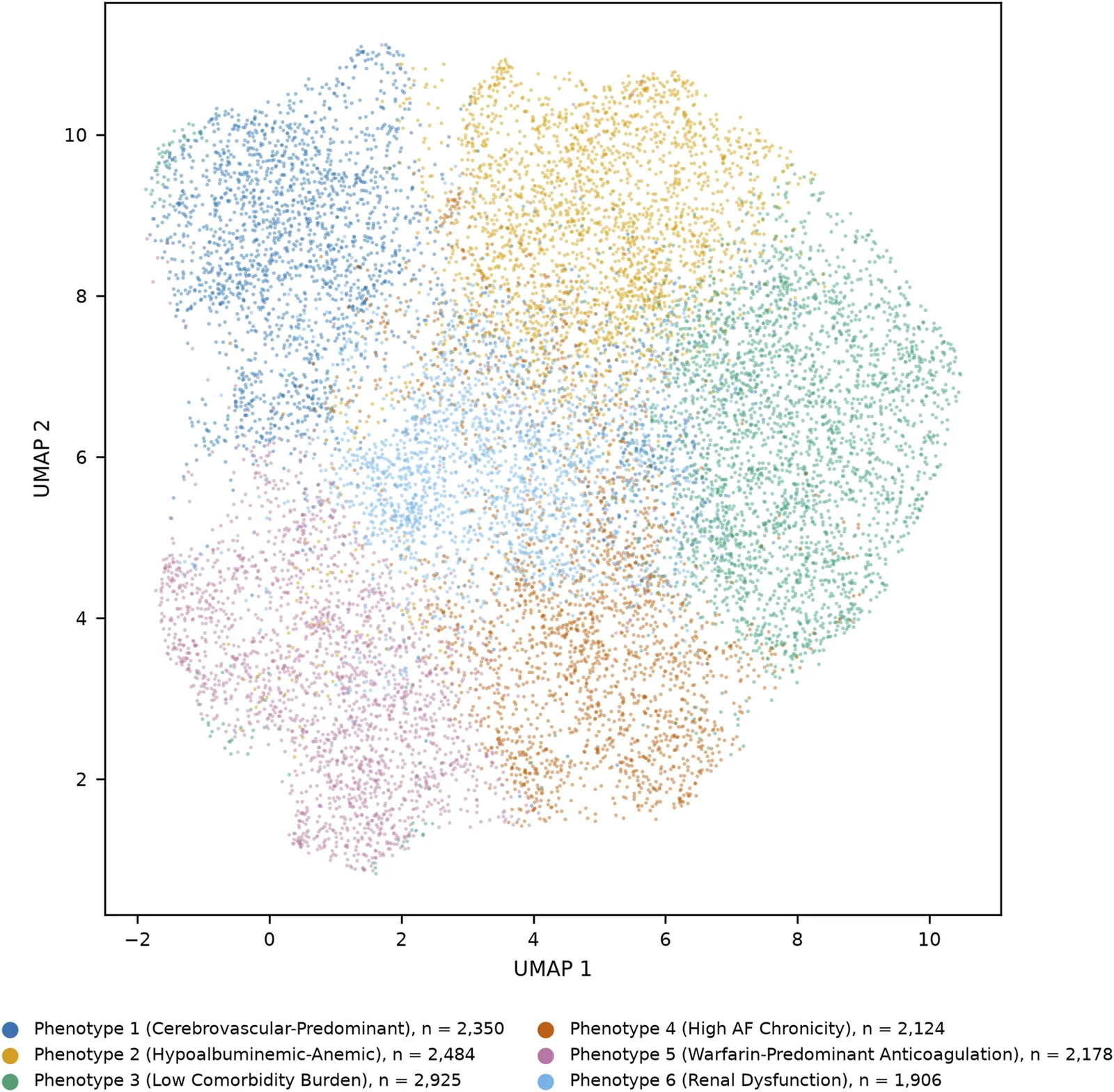
UMAP projection of atrial fibrillation phenotypes. Uniform Manifold Approximation and Projection visualization of patient embeddings derived from the 10-dimensional VAE latent space. Colors represent the six identified phenotypes. Abbreviations: UMAP = Uniform Manifold Approximation and Projection; VAE = variational autoencoder

Exclusion criteria: (1) atrial fibrillation recorded only under the Ninth Revision (ICD-9 427.3x). Case ascertainment used ICD-10 codes exclusively, so patients whose atrial fibrillation was coded only under ICD-9 were never identified, are absent from the cohort by construction, and do not appear as a counted exclusion at any node of Figure 1; (2) codes within the I48 family that fall outside the six-code list, including the atrial flutter codes I48.3, I48.4, and I48.92 and the less specific parent codes I48.1 and I48.2, which were not treated as qualifying diagnoses; and (3) membership of the 2008 to 2010 or 2011 to 2013 anchor year group.

No exclusions were applied on the basis of age, sex, comorbidity, length of stay, completeness of laboratory data, or vital status, and no patient was excluded for missing data at any later stage of the analysis.

### Sample Size

No a priori sample size or power calculation was performed. The entire eligible cohort available in the data source was analyzed, so the sample size was determined by the number of patients meeting the eligibility criteria rather than by a target precision, and no hypothesis test reported here was powered in advance. This is acknowledged as a limitation in the Discussion.

The precision actually achieved can be stated post hoc. The analytic cohort comprised 13,967 patients with 4,531 deaths (32.4%). The smallest phenotype contained 1,906 patients and no phenotype contained fewer than 324 deaths. The resulting precision is illustrated by the smallest and largest phenotype hazard ratios in the primary adjusted Cox model, 3.52 (95% CI 3.06 to 4.05) for Phenotype 3 (Low Comorbidity Burden) and 7.20 (95% CI 6.49 to 7.99) for Phenotype 2 (Hypoalbuminemic-Anemic), and by the 95% confidence interval of 0.725 to 0.742 around the apparent concordance index of 0.733 for the model containing the phenotype alongside age, sex, CHA_2_DS_2_-VASc, and three routine laboratory values.

### Reporting Guideline

This study is reported in accordance with the STROBE (Strengthening the Reporting of Observational Studies in Epidemiology) statement for cohort studies^18^ and with the RECORD (REporting of studies Conducted using Observational Routinely-collected health Data) extension for research conducted using routinely collected health data,^19^ and the completed STROBE and RECORD checklist is provided as a supplementary file (Supplementary Checklist S1), including the items that are reported only in part and the one item that is not reported.

### Feature Engineering

A patient-level feature matrix was constructed by aggregating clinical information across all encounters. Features spanned demographics, AF characteristics, comorbidities, clinical risk scores, medications, and healthcare utilization, and 35 of them were supplied to the representation-learning and clustering model (Supplementary Table S1). Binary comorbidity and medication variables were encoded using an “ever” definition, set to 1 if the corresponding diagnosis code or prescription appeared in any encounter in the patient’s record. Continuous laboratory measures were summarized as the median across encounters. Laboratory features with less than 30% patient-level coverage were excluded from model input.

Three feature definitions require explicit statement, because their names are less specific than their construction. First, the AF chronicity variable is the interval in years between a patient’s first and last AF-coded admission, and it is referred to throughout as the AF coded admission interval. It measures the span of coded contact with the health system rather than the duration of the arrhythmia, and it is 0.0 by construction for the 6,927 patients (49.6%) with a single admission. Relatedly, AF subtype severity was taken as the most severe AF subtype code recorded anywhere in the record; of the 499 patients ever coded longstanding persistent or permanent AF, only 280 (56.1%) still carry that code at the index admission. Second, the HAS-BLED score as implemented here omits the labile international normalized ratio component, which is not recoverable from these data, and substitutes an indicator of warfarin exposure in its place. HAS-BLED is therefore partly a treatment variable in this study, and it shares that warfarin term with the feature that most distinguishes Phenotype 5 (Warfarin-Predominant Anticoagulation). Third, both CHA_2_DS_2_-VASc and HAS-BLED were computed from the same patient-level “ever” comorbidity flags rather than from values present at the index encounter; relative to index-admission values, this cumulative encoding raises the mean CHA_2_DS_2_-VASc by 0.29 points and the mean HAS-BLED by 0.43 points.

### Missingness Assessment and Imputation

Missingness was assessed for all 42 candidate patient-level variables before imputation. Seven were excluded from model input as data-availability artifacts rather than clinical measurements: four vital sign variables (mean and maximum heart rate, mean and minimum systolic blood pressure), which had no recorded values in the derived dataset, and three electrocardiography-derived variables, whose availability depends on linkage to a separate electrocardiogram archive covering only part of the cohort. That left the 35 features used for representation learning and clustering. Of those 35, 29 had no missing values, 5 had missingness below 5% (creatinine, hemoglobin, potassium, sodium, and the international normalized ratio), and 1, serum albumin, had 20.4% missingness. No feature retained for modeling had more than 30% missing values.

For clustering-eligible continuous features with <30% missingness, K-nearest neighbors (KNN) imputation (k=5) was applied, leveraging local data structure to estimate missing values. Remaining missing values were imputed with population medians.

### Variational Autoencoder for Representation Learning

We employed a variational autoencoder (VAE) for nonlinear dimensionality reduction of 35 clustering-eligible features. The encoder used two hidden layers (64 and 32 units) with ReLU activation and 20% dropout, producing a 10-dimensional latent representation. The decoder mirrored the encoder and used separate output heads for binary and continuous features. Reconstruction loss combined binary cross-entropy (binary variables) and mean squared error (continuous variables), plus a beta-weighted KL divergence term (beta = 0.5). Binary features remained on the 0/1 scale, and continuous features were standardized prior to training.

Hyperparameters were selected via grid search over latent dimensions (8, 10, 12), beta values (0.5, 1.0, 2.0), and learning rates (0.001, 0.0005), choosing the configuration with the lowest validation reconstruction loss; the selected configuration was a latent dimension of 10, beta of 0.5, and a learning rate of 0.0005 (Supplementary Table S2). Training used the Adam optimizer, batch size 64, and early stopping (patience 20; maximum 200 epochs), with convergence at epoch 148 (Supplementary Figure S1). Model selection was performed once on the full cohort, and the selected configuration and the number of clusters were then held fixed in every resampling and sensitivity analysis reported below, so none of those analyses propagates uncertainty in model selection.

### Phenotype Discovery and Characterization

K-means clustering was applied to patient latent vectors, and k = 2 through 8 were evaluated (Supplementary Table S3). Four internal validity indices computed on the primary latent space did not converge on a common solution: the silhouette coefficient was maximal at k=6 (0.1386), the gap statistic at k=7 (2.4767), the Calinski-Harabasz index at k=4 (1,722), and the Davies-Bouldin index at k=8 (1.568). Across k=4 to k=8 the silhouette coefficient varied by only 0.017 and inertia declined gradually without a sharp elbow, so no index distinguished these solutions to a meaningful degree. We therefore compared the k=4 through k=8 solutions directly on three grounds, stated before the comparison rather than chosen after it (Supplementary Figure S9, Supplementary Table S9). First, reproducibility: each solution was resampled 50 times at 80% of the cohort with k-means refitting and nearest-centroid assignment of the full cohort, and all five were highly reproducible (adjusted Rand index 0.889 to 0.955), with the maximum and the tightest dispersion at k=6 (0.955, standard deviation 0.010). Second, per-cluster statistical power for the survival analyses: at k=6 the smallest cluster contained 1,906 patients and every cluster contained at least 324 deaths, whereas at k=8 these fell to 936 patients and 165 deaths. Third, interpretability: the cross-classification of solutions showed that k=4 dispersed two of the six profiles, while seven of the eight clusters at k=8 drew the majority of their members from a single k=6 phenotype and therefore subdivided existing profiles rather than adding new ones. k=6 was selected on these grounds and is one of several statistically near-equivalent solutions rather than an optimum identified by any single index. Cluster profiles were summarized using standardized z-scores computed within each cluster relative to the overall cohort. Phenotype labels were assigned post hoc based on the dominant distinguishing features (|z| > 0.5), rather than predefined templates.

Phenotype labels are post hoc descriptive shorthand for the standardized feature profile of each cluster and are not adjudicated clinical categories. In particular, the label High AF Chronicity denotes a cluster distinguished by repeated AF-coded contact and by the recorded AF subtype code, and does not represent a clinically adjudicated diagnosis of longstanding persistent or permanent AF. Labels name the features that separate a cluster and carry no implication about the appropriateness of the care those patients received; interpretive statements about the phenotypes are confined to the Discussion. Throughout this report the phenotypes are designated Phenotype 1 (Cerebrovascular-Predominant), Phenotype 2 (Hypoalbuminemic-Anemic), Phenotype 3 (Low Comorbidity Burden), Phenotype 4 (High AF Chronicity), Phenotype 5 (Warfarin-Predominant Anticoagulation), and Phenotype 6 (Renal Dysfunction).

### Survival Analysis and Follow-Up

Time zero was the admission time of the index encounter, defined as the first hospital admission carrying an ICD-10 I48 atrial fibrillation diagnosis code. Time zero was not the first admission of any type in the record: 3,525 patients (25.2%) had at least one earlier admission without an atrial fibrillation code in MIMIC-IV, a median of 621.6 days (interquartile range 150.8 to 1,260.6 days) before the index encounter. Patients who died were followed to the recorded date of death; patients not known to have died were censored at the latest discharge time recorded across all of their encounters, with follow-up floored at 1 day.

Because patients not known to have died were censored at the latest discharge time in the record, censoring time is structurally a measure of healthcare utilization. Half of the cohort (6,927 patients, 49.6%) had a single admission, and 4,904 patients (35.1%) had a single admission and were censored alive; for these patients follow-up time is identically the index length of stay (Pearson r = 0.9999). The proportion of patients whose follow-up is determined in this way varied from 12.9% in Phenotype 4 (High AF Chronicity) to 61.1% in Phenotype 3 (Low Comorbidity Burden). Cumulative length of stay and number of encounters are also inputs to the clustering model, so follow-up duration and phenotype membership are not independent. MIMIC-IV contains no outpatient encounters, so apart from the recorded date of death no follow-up information is available between or after hospital admissions.

Phenotype 4 (High AF Chronicity) was used as the reference category in Cox models. The reference was selected on pre-stated criteria: at least 1,000 patients and at least 100 deaths, then the longest reverse Kaplan-Meier median follow-up, with the lowest proportion censored as a tiebreaker. Phenotype 4 had a reverse Kaplan-Meier median follow-up of 854.9 days against 5.3 days for Phenotype 3 (Low Comorbidity Burden), which served as the reference in the original analysis, a censoring proportion of 70.9% against 88.9%, and 1,215 patients at risk at 1 year against 164. Phenotype 4 is the best observed phenotype rather than an unbiased one: its follow-up advantage is itself utilization driven.

Follow-up duration was quantified by the reverse Kaplan-Meier method overall and within each phenotype, and numbers at risk are reported at 0, 30, 90, 180, 365, 730, and 1,095 days. Independence of censoring from phenotype was tested formally using a reverse Kaplan-Meier log-rank test across phenotypes, a Cox model of the censoring hazard on phenotype, and Spearman rank correlations between follow-up time and healthcare utilization among censored patients. Two pre-stated analyses addressed early censoring bias: landmark analyses restricted to patients alive and under observation at 30 days and at 90 days, with time reset to the landmark, and administrative censoring of all patients at a common 1-year horizon. Because observation windows differ markedly across phenotypes, crude mortality figures are reported as observed-death proportions rather than as risks, and identification bounds for 1-year mortality are reported for the phenotypes with the shortest follow-up.

### Statistical Analysis

Baseline differences across phenotypes were tested using chi-square tests for categorical variables and Kruskal-Wallis tests for continuous variables. Survival was evaluated with Kaplan-Meier curves and a global log-rank test; pairwise log-rank tests across all phenotype pairs used Bonferroni correction (alpha = 0.0033). Cox proportional hazards models estimated hazard ratios for all-cause mortality against Phenotype 4 (High AF Chronicity) as the reference, which was selected on the pre-stated follow-up criteria described above rather than on mortality. Three models are reported: unadjusted, a primary model adjusted for age and sex only, and a secondary model that additionally adjusts for CHA_2_DS_2_-VASc. Adjustment was restricted to age and sex in the primary model because all eight CHA_2_DS_2_-VASc component terms, and the composite score itself, were supplied to the clustering model, so adjusting for the score conditions on variables that helped define the exposure. Age and sex were themselves supplied to the clustering model, so this adjustment set reduces rather than eliminates overlap between the covariates and the phenotype definitions. Variance inflation factors were computed on the design matrix of the secondary model. The proportional hazards assumption was assessed for every model using Schoenfeld residual tests, per covariate and globally, with rank and Kaplan-Meier time transforms; where the assumption was rejected, hazard ratios were additionally estimated within disjoint follow-up intervals. Treatment pattern differences were assessed with chi-square testing.

Incremental discrimination was assessed with nested Cox models for all-cause mortality using Harrell’s concordance index and likelihood ratio tests. Four models were pre-stated: CHA_2_DS_2_-VASc alone; age, sex, and CHA_2_DS_2_-VASc; that model plus three routinely measured laboratory values (creatinine, hemoglobin, and albumin); and that model plus the phenotype, entered as five indicator terms with Phenotype 4 (High AF Chronicity) as the reference. A further model added encounter count, any intensive care admission, and total length of stay without the phenotype, as a conventional comparator built only from routinely available variables. Optimism was estimated by Harrell’s bootstrap with 500 resamples, refitting every model on each resample, evaluating it on the resample and on the original cohort, averaging the difference, and subtracting it from the apparent value. Because the phenotypes were learned from the full cohort, discrimination was also estimated by three repeats of stratified five-fold cross-validation in which the standardizer, the variational autoencoder, and the k-means solution were refitted from scratch within each training fold and validation patients were assigned to the nearest training-fold centroid. Cumulative/dynamic time-dependent areas under the curve at 30, 90, and 365 days were computed with inverse probability of censoring weights and are reported descriptively, because the independence assumption those weights require is contradicted by the censoring analysis described above. Creatinine and hemoglobin were observed in 99.6% and 99.5% of patients and albumin in 79.6%; all three were k-nearest-neighbor imputed during feature engineering, so no patient was excluded from these models.

Analyses were performed in Python 3.12 using scikit-learn, PyTorch, lifelines, statsmodels, and scipy. All random processes were seeded, and the seed is recorded with each analysis output.

### Validation and Sensitivity Analyses

We conducted three validation strategies. First, temporal split validation trained the VAE on the earliest anchor year group (2014 to 2016) and evaluated the later groups (2017 to 2019 and 2020 to 2022), comparing phenotype distributions against a pre-specified threshold of ±5 percentage points and comparing outcome associations. Because temporal validation retrains both the VAE and K-means, temporal cluster identities were aligned to the primary phenotypes using Hungarian matching based on cosine similarity of z-score profiles. Second, bootstrap stability was assessed at two levels, each over 100 iterations of 80% subsampling. In the first, the trained variational autoencoder was held fixed and only K-means was refit, isolating the reproducibility of the partition given the learned representation. In the second, end-to-end variant, the standardizer and the variational autoencoder were refit from scratch on each subsample using the same selected hyperparameters and early stopping criterion, K-means was refit in the resulting latent space, and the full cohort was assigned by nearest centroid; bootstrap clusters were matched to the primary solution by the Hungarian algorithm before scoring. Both variants report pair-counting Jaccard similarity, the Adjusted Rand Index, per-cluster matched Jaccard, the pairwise consensus distribution, and the Proportion of Ambiguous Clustering, defined as the fraction of patient pairs whose consensus value falls between 0.1 and 0.9. As an empirical null, the entire end-to-end procedure was repeated over 50 iterations on a permuted dataset in which every feature column was independently shuffled, preserving all marginal distributions while destroying the joint structure. Third, sensitivity analyses tested alternate cluster counts (k ± 2) and latent dimensions (±2).

Because three of the input variables describe healthcare utilization rather than baseline clinical status, the entire representation learning and clustering procedure was repeated on reduced feature sets. In the first variant, cumulative length of stay, number of encounters, and any intensive care admission were removed, leaving 32 baseline clinical variables. In the second variant, the number of AF-coded encounters and the AF coded admission interval were also removed, leaving 30 variables. In each variant the variational autoencoder was retrained from scratch with the same architecture, the same selected hyperparameters (latent dimension 10, beta 0.5, learning rate 0.0005) and the same early stopping rule, and patients were re-clustered at k = 6. Cluster names were re-derived from standardized feature profiles rather than carried over, and the resulting clusters are lettered BO1-A to BO1-F for the first variant and BO2-A to BO2-F for the second, to distinguish them both from the six phenotypes of the primary analysis and from the index-admission clusters described next. Agreement with the primary solution was quantified by the adjusted Rand index and adjusted mutual information. Because cluster identifiers are not stable when the encoder is refit, each variant was fit ten times from different random initializations, and an additional control in which the unchanged 35-variable feature set was refit the same ten times was used as the benchmark against which agreement was judged.

In a further pre-stated sensitivity analysis addressing the cumulative encoding of the clustering features, every comorbidity, medication, clinical score and laboratory variable was recomputed using only data recorded at the index admission, using identical International Classification of Diseases code sets, identical medication keyword lists and identical score component definitions. Three features (total encounters, the AF coded admission interval and the number of AF-coded encounters) have no index-admission counterpart because they are defined over the whole record, and four laboratory analytes (potassium, sodium, albumin and the international normalized ratio) are not carried at admission level in the derived dataset; all seven were excluded, leaving 28 features. The variational autoencoder was retrained from scratch on this matrix with the same architecture, hyperparameters and cluster number, and the resulting clusters, lettered IA-A to IA-F, were compared with the primary solution using the adjusted Rand index and adjusted mutual information. Two controls were run under the same protocol so that the comparison could be interpreted: a refit control that used the unchanged 35-feature matrix and was refit with a fresh random initialization and a fresh training and validation split, and a feature-restriction control that used the same 28 features with the original cumulative values. Because agreement between two refits of the same specification is itself well below one, every comparison is reported against a refit benchmark rather than against complete agreement. This analysis and the baseline-only re-clustering above remove different things and are reported separately; they are not combined into a single statement about robustness.

## Results

### Study Cohort

From 364,627 patients in MIMIC-IV, 20,952 had at least one diagnosis code in the I48 family. Restricting case ascertainment to the six ICD-10 codes listed in the Methods removed 1,519 patients whose only I48 codes fell outside that list, namely the atrial flutter codes I48.3, I48.4, and I48.92 and the less specific parent codes I48.1 and I48.2, leaving 19,433 patients; excluding the 2008 to 2010 and 2011 to 2013 anchor year groups removed a further 5,466, leaving 13,967 patients with 33,501 total encounters, who comprised the final cohort (Figure 1). No node of the flow diagram excludes patients coded only under the Ninth Revision, because ICD-9 codes were never used for case ascertainment.

### Phenotype Identification and Characterization

The VAE (latent dimension=10, β=0.5, learning rate=0.0005) converged at epoch 148 via early stopping. K-Means clustering with k=6 identified six AF phenotypes (silhouette=0.1386; Supplementary Figure S2), visualized in UMAP space (Figure 2).

Observed-death proportions ranged from 11.1% to 48.1% across the six phenotypes, a spread of 37.0 percentage points (Table 1). These proportions accrued over markedly unequal observation windows and are not comparable risks; follow-up, censoring, and the identification limits that follow from them are reported below.

**Table 1.** Baseline Characteristics by AF Phenotype

Feature	Overall	Phenotype 1 (Cerebrovascular-predominant)	Phenotype 2 (Hypoalbuminemic-anemic)	Phenotype 3 (Low comorbidity burden)	Phenotype 4 (High AF chronicity)	Phenotype 5 (Warfarin-predominant anticoagulation)	Phenotype 6 (Renal dysfunction)	p-value
Female	5,765 (41.3%)	1,255 (53.4%)	1,206 (48.6%)	905 (30.9%)	967 (45.5%)	812 (37.3%)	620 (32.5%)	< 0.001
Race: White	10,293 (73.7%)	1,572 (66.9%)	1,725 (69.4%)	2,320 (79.3%)	1,689 (79.5%)	1,652 (75.8%)	1,335 (70.0%)	< 0.001
Heart Failure	6,265 (44.9%)	956 (40.7%)	964 (38.8%)	430 (14.7%)	1,505 (70.9%)	1,176 (54.0%)	1,234 (64.7%)	< 0.001
Hypertension	11,410 (81.7%)	2,177 (92.6%)	1,725 (69.4%)	1,938 (66.3%)	1,970 (92.7%)	1,819 (83.5%)	1,781 (93.4%)	< 0.001
Diabetes Mellitus	4,472 (32.0%)	872 (37.1%)	637 (25.6%)	494 (16.9%)	733 (34.5%)	678 (31.1%)	1,058 (55.5%)	< 0.001
Chronic Kidney Disease	4,185 (30.0%)	537 (22.9%)	488 (19.6%)	118 (4.0%)	840 (39.5%)	692 (31.8%)	1,510 (79.2%)	< 0.001
Prior Stroke/TIA	2,887 (20.7%)	1,888 (80.3%)	194 (7.8%)	89 (3.0%)	282 (13.3%)	215 (9.9%)	219 (11.5%)	< 0.001
CAD/MI	6,414 (45.9%)	1,227 (52.2%)	798 (32.1%)	819 (28.0%)	1,125 (53.0%)	1,096 (50.3%)	1,349 (70.8%)	< 0.001
COPD	3,534 (25.3%)	475 (20.2%)	740 (29.8%)	522 (17.8%)	722 (34.0%)	552 (25.3%)	523 (27.4%)	< 0.001
Valvular Heart Disease	1,440 (10.3%)	217 (9.2%)	185 (7.4%)	153 (5.2%)	357 (16.8%)	342 (15.7%)	186 (9.8%)	< 0.001
Obesity	2,545 (18.2%)	291 (12.4%)	449 (18.1%)	438 (15.0%)	550 (25.9%)	499 (22.9%)	318 (16.7%)	< 0.001
Obstructive Sleep Apnea	2,194 (15.7%)	271 (11.5%)	274 (11.0%)	415 (14.2%)	590 (27.8%)	351 (16.1%)	293 (15.4%)	< 0.001
Beta-Blocker	11,038 (79.0%)	1,895 (80.6%)	2,048 (82.4%)	1,887 (64.5%)	1,877 (88.4%)	1,841 (84.5%)	1,490 (78.2%)	< 0.001
Calcium Channel Blocker	2,922 (20.9%)	475 (20.2%)	735 (29.6%)	475 (16.2%)	628 (29.6%)	401 (18.4%)	208 (10.9%)	< 0.001
Digoxin	1,361 (9.7%)	198 (8.4%)	340 (13.7%)	104 (3.6%)	347 (16.3%)	273 (12.5%)	99 (5.2%)	< 0.001
Antiarrhythmic	4,345 (31.1%)	517 (22.0%)	802 (32.3%)	710 (24.3%)	806 (37.9%)	893 (41.0%)	617 (32.4%)	< 0.001
Warfarin	3,597 (25.8%)	382 (16.3%)	245 (9.9%)	57 (1.9%)	562 (26.5%)	1,948 (89.4%)	403 (21.1%)	< 0.001
DOAC	5,995 (42.9%)	1,031 (43.9%)	1,020 (41.1%)	1,285 (43.9%)	1,710 (80.5%)	252 (11.6%)	697 (36.6%)	< 0.001
ACE-I/ARB	5,618 (40.2%)	1,139 (48.5%)	718 (28.9%)	905 (30.9%)	1,273 (59.9%)	900 (41.3%)	683 (35.8%)	< 0.001
Statin	9,005 (64.5%)	1,814 (77.2%)	1,196 (48.1%)	1,392 (47.6%)	1,604 (75.5%)	1,532 (70.3%)	1,467 (77.0%)	< 0.001
Has ECG	5,265 (37.7%)	877 (37.3%)	800 (32.2%)	968 (33.1%)	956 (45.0%)	954 (43.8%)	710 (37.3%)	< 0.001
ECG AF Detected	4,131 (29.6%)	683 (29.1%)	646 (26.0%)	722 (24.7%)	724 (34.1%)	834 (38.3%)	522 (27.4%)	< 0.001
Any ICU Admission	8,279 (59.3%)	1,783 (75.9%)	1,933 (77.8%)	984 (33.6%)	951 (44.8%)	1,416 (65.0%)	1,212 (63.6%)	< 0.001
Age at First AF	76.0 (67.0-84.0)	80.0 (73.0-86.0)	72.0 (64.0-81.0)	70.0 (61.0-79.0)	77.0 (70.0-85.0)	75.0 (67.0-83.0)	79.0 (72.0-87.0)	< 0.001
Total Encounters	2.0 (1.0-3.0)	1.0 (1.0-3.0)	2.0 (1.0-3.0)	1.0 (1.0-2.0)	3.0 (2.0-5.0)	1.0 (1.0-2.0)	1.0 (1.0-3.0)	< 0.001
AF Subtype Severity	1.0 (1.0-3.0)	1.0 (1.0-3.0)	1.0 (1.0-3.0)	1.0 (1.0-3.0)	3.0 (3.0-4.0)	1.0 (1.0-3.0)	1.0 (1.0-3.0)	< 0.001
AF Coded Admission Interval (Years)	0.0 (0.0-0.1)	0.0 (0.0-0.0)	0.0 (0.0-0.1)	0.0 (0.0-0.0)	1.3 (0.0-3.1)	0.0 (0.0-0.0)	0.0 (0.0-0.0)	< 0.001
AF Encounters	1.0 (1.0-2.0)	1.0 (1.0-2.0)	1.0 (1.0-2.0)	1.0 (1.0-1.0)	2.0 (2.0-4.0)	1.0 (1.0-2.0)	1.0 (1.0-2.0)	< 0.001
CHA2DS2-VASc	4.0 (3.0-5.0)	6.0 (5.0-7.0)	4.0 (2.0-5.0)	3.0 (2.0-4.0)	5.0 (4.0-6.0)	4.0 (3.0-5.0)	5.0 (4.0-6.0)	< 0.001
HAS-BLED	4.0 (2.0-5.0)	4.0 (4.0-5.0)	3.0 (2.0-4.0)	2.0 (2.0-3.0)	4.0 (3.0-5.0)	4.0 (3.0-5.0)	4.0 (3.0-5.0)	< 0.001
Creatinine (Median)	1.0 (0.8-1.3)	0.9 (0.7-1.1)	0.9 (0.7-1.2)	0.9 (0.8-1.0)	1.0 (0.8-1.3)	1.0 (0.8-1.4)	1.9 (1.3-3.3)	< 0.001
Hemoglobin (Median)	10.6 (9.0-12.3)	11.0 (9.6-12.4)	8.9 (8.2-10.1)	12.5 (11.1-13.7)	11.2 (9.7-12.7)	10.4 (9.1-11.8)	9.2 (8.4-10.5)	< 0.001
Potassium (Median)	4.2 (4.0-4.4)	4.1 (3.9-4.3)	4.0 (3.9-4.2)	4.2 (4.0-4.4)	4.1 (4.0-4.3)	4.1 (4.0-4.3)	4.5 (4.2-4.7)	< 0.001
Sodium (Median)	139.0 (137.0-141.0)	140.0 (138.0-142.0)	139.0 (136.5-141.0)	139.0 (137.5-141.0)	139.0 (137.5-141.0)	138.5 (136.5-140.5)	138.0 (135.0-140.0)	< 0.001
Albumin (Median)	3.6 (3.2-4.0)	3.7 (3.3-4.0)	3.0 (2.6-3.3)	4.0 (3.7-4.3)	3.8 (3.4-4.0)	3.7 (3.2-4.0)	3.5 (3.1-3.8)	< 0.001
INR (Median)	1.3 (1.2-1.6)	1.2 (1.1-1.4)	1.3 (1.2-1.4)	1.2 (1.1-1.3)	1.4 (1.2-1.6)	1.9 (1.4-2.4)	1.3 (1.2-1.5)	< 0.001
Total LOS (Days)	9.8 (4.9-21.0)	10.7 (5.7-19.7)	20.0 (9.4-42.1)	4.6 (1.9-7.8)	14.6 (7.5-28.4)	9.0 (5.3-17.4)	10.8 (5.5-21.4)	< 0.001
Observed deaths, n (%)	4,531 (32.4%)	883 (37.6%)	1,181 (47.5%)	324 (11.1%)	618 (29.1%)	608 (27.9%)	917 (48.1%)	< 0.001
Observed median follow-up (days, IQR)	18 (6-194)	15 (6-128)	31 (10-132)	5 (2-14)	544 (57-1203)	13 (6-98)	20 (6-154)	N/A

Continuous variables: median (IQR). Categorical variables: n (%). P-values: Kruskal-Wallis (continuous) or chi-square (categorical). Observed deaths are counts of deaths recorded during follow-up and accrue over markedly unequal observation windows; reverse Kaplan-Meier follow-up and censoring by phenotype are given in Supplementary Table S8. The AF coded admission interval is the interval in years between a patient’s first and last AF-coded admission and is 0.0 for patients with a single admission. Has ECG and ECG AF Detected are reported as baseline descriptors and were not supplied to the clustering model.

Phenotype 1 (Cerebrovascular-Predominant) (n = 2,350; 883 observed deaths, 37.6%) was distinguished by elevated prior stroke or transient ischemic attack (z = +1.47), elevated CHA_2_DS_2_-VASc (z = +0.99), and elevated HAS-BLED (z = +0.57).

Phenotype 2 (Hypoalbuminemic-Anemic) (n = 2,484; 1,181 observed deaths, 47.5%) was distinguished by low albumin (z = -0.99), low hemoglobin (z = -0.74), and elevated cumulative length of stay (z = +0.66), with hypertension below the cohort mean (z = -0.32).

Phenotype 3 (Low Comorbidity Burden) (n = 2,925; 324 observed deaths, 11.1%) was distinguished by elevated median hemoglobin (z = +0.78), elevated median albumin (z = +0.69), low HAS-BLED (z = -0.93), and low CHA_2_DS_2_-VASc (z = -0.86).

Phenotype 4 (High AF Chronicity) (n = 2,124; 618 observed deaths, 29.1%) was distinguished by an elevated AF coded admission interval (z = +1.34), elevated AF subtype severity (z = +1.10), and an elevated number of AF-coded encounters (z = +1.00).

Phenotype 5 (Warfarin-Predominant Anticoagulation) (n = 2,178; 608 observed deaths, 27.9%) was distinguished by elevated warfarin exposure (z = +1.46), elevated median international normalized ratio (z = +1.19), and low direct oral anticoagulant exposure (z = -0.63).

**Figure 3.**
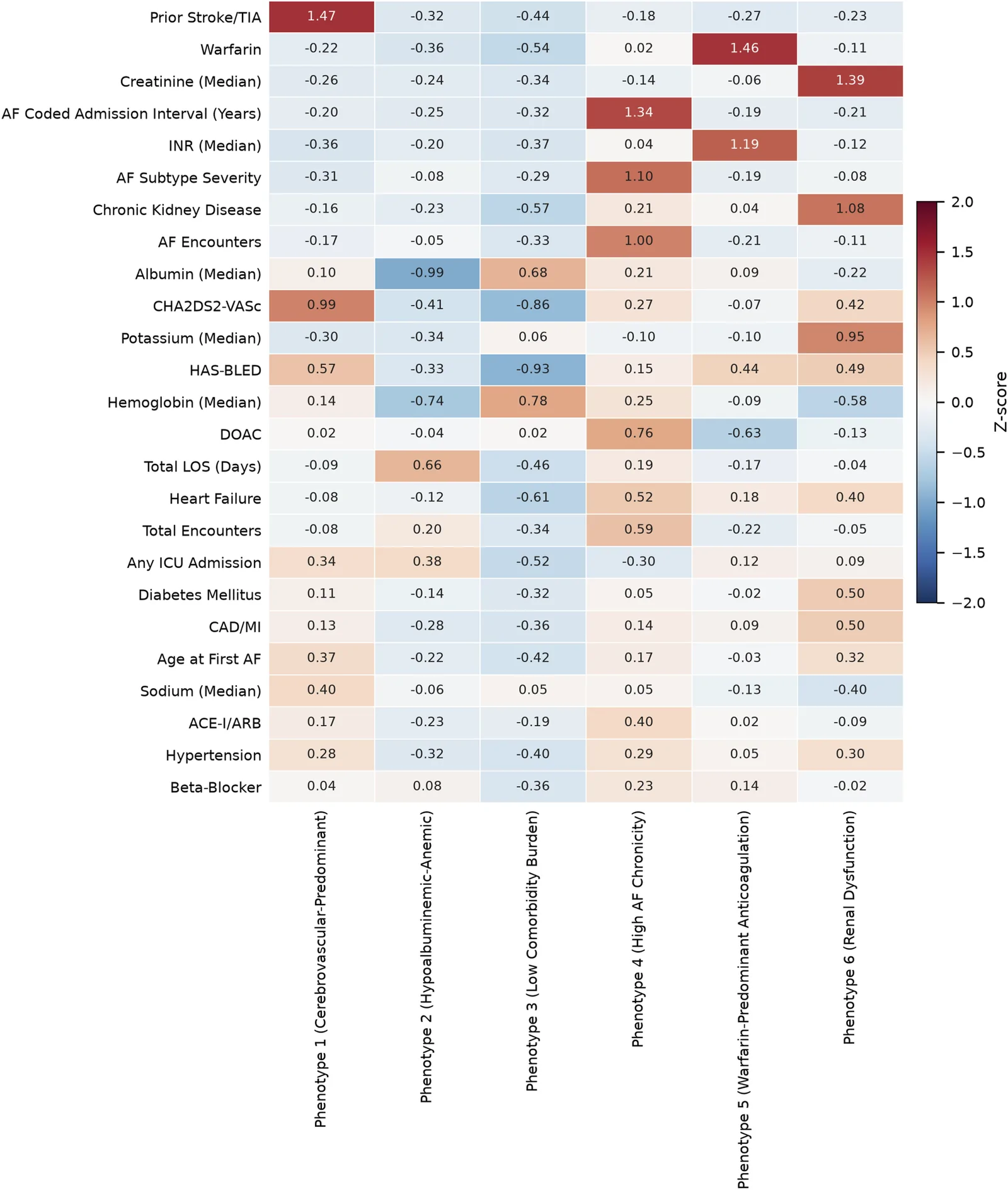
Standardized feature profiles by phenotype. Heatmap of phenotype-specific standardized z-scores relative to the overall cohort. Red indicates values above the cohort mean; blue indicates values below the cohort mean

Phenotype 6 (Renal Dysfunction) (n = 1,906; 917 observed deaths, 48.1%) was distinguished by elevated median creatinine (z = +1.39), elevated chronic kidney disease (z = +1.07), elevated median potassium (z = +0.95), and low median hemoglobin (z = -0.58). The standardized z-score profiles for all six phenotypes are shown in Figure 3 and reported numerically for all 35 clustering features in Supplementary Table S4.

Distributions of CHA_2_DS_2_-VASc and HAS-BLED scores across phenotypes are shown in Supplementary Figure S3.

A radar plot of key clinical features by phenotype is provided in Supplementary Figure S4.

### Baseline Characteristics

Baseline characteristics across the six phenotypes are summarized in Table 1. Significant differences were observed across all demographic, comorbidity, medication, and laboratory features (all p<0.001).

### Primary Outcome

**Figure 4.**
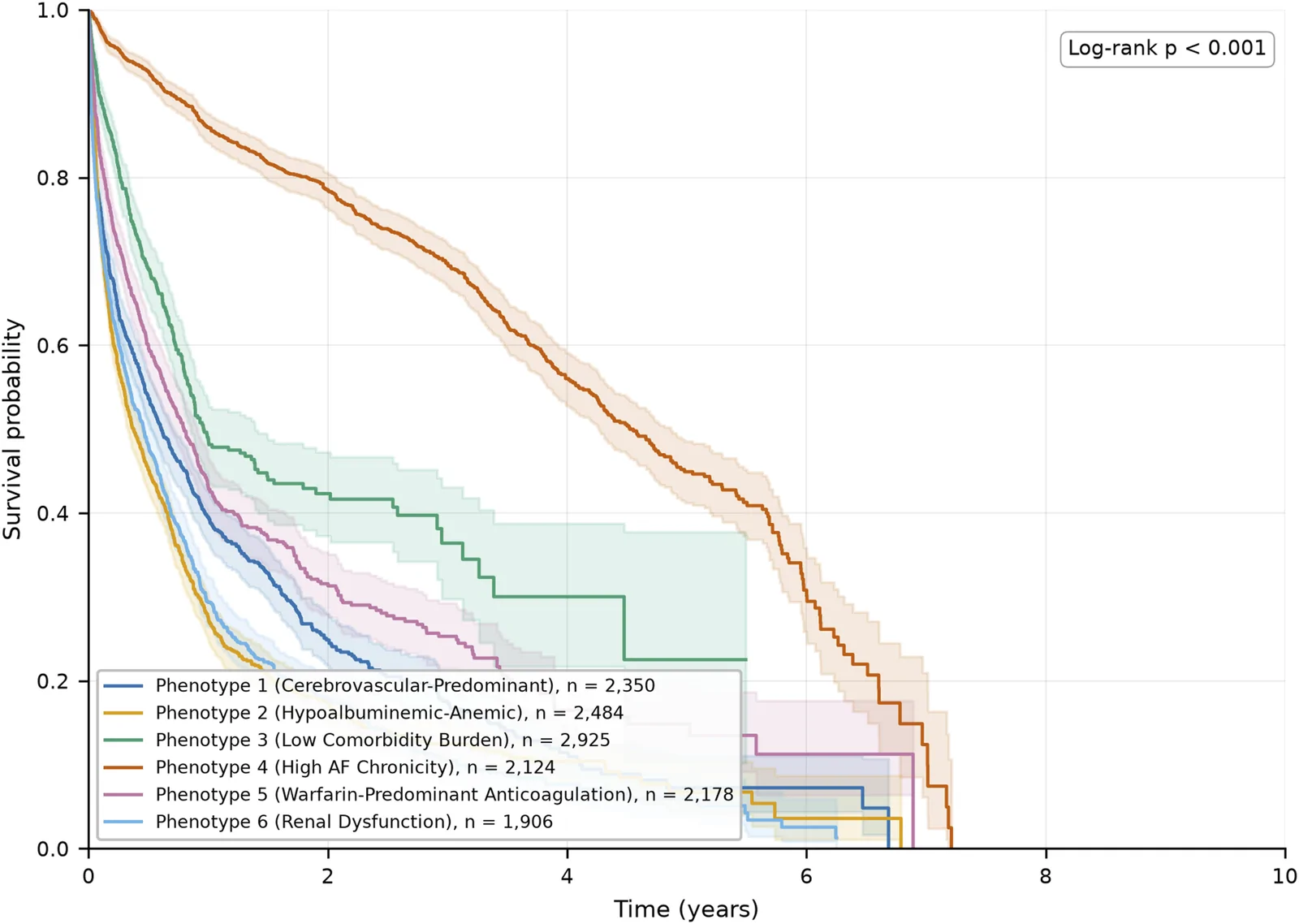
Kaplan-Meier survival by phenotype. Kaplan-Meier curves comparing all-cause mortality across six atrial fibrillation phenotypes. Survival differences were assessed using the log-rank test (p < 0.001). Numbers at risk by phenotype and follow-up time are given in Supplementary Table S11

All-cause mortality differed significantly across phenotypes (global log-rank chi-square 1,594.7 on 5 degrees of freedom, p < 0.001; Figure 4). Pairwise log-rank tests showed that 14 of 15 comparisons were significant after Bonferroni correction (p < 0.0033). The only non-significant comparison was Phenotype 2 (Hypoalbuminemic-Anemic) versus Phenotype 6 (Renal Dysfunction) (p = 0.71), which had similar observed-death proportions (Supplementary Table S7).

Phenotype 4 (High AF Chronicity) served as the reference category in all Cox models and was selected on the pre-stated follow-up criteria given in the Methods rather than on mortality. Unadjusted hazard ratios for all-cause mortality against Phenotype 4 ranged from 2.64 (95% CI 2.30 to 3.04) for Phenotype 3 (Low Comorbidity Burden) to 5.75 (95% CI 5.17 to 6.39) for Phenotype 6 (Renal Dysfunction), all p < 0.001 (Table 2).

**Table 2.** Cox Proportional Hazards Models for All-Cause Mortality by Atrial Fibrillation Phenotype, With Phenotype 4 (High AF Chronicity) as the Reference

Variable	Model 1, unadjusted: HR (95% CI)	Model 1 P value	Model 2, adjusted for age and sex (primary): HR (95% CI)	Model 2 P value	Model 3, adjusted for age, sex and CHA_2_DS_2_-VASc (secondary): HR (95% CI)	Model 3 P value	Variance inflation factor in model 3
Phenotype 1 (Cerebrovascular-Predominant)	4.66 (4.19 to 5.19)	< 0.001	4.35 (3.91 to 4.84)	< 0.001	4.62 (4.13 to 5.16)	< 0.001	1.89
Phenotype 2 (Hypoalbuminemic-Anemic)	5.68 (5.13 to 6.29)	< 0.001	7.20 (6.49 to 7.99)	< 0.001	6.91 (6.21 to 7.69)	< 0.001	1.91
Phenotype 3 (Low Comorbidity Burden)	2.64 (2.30 to 3.04)	< 0.001	3.52 (3.06 to 4.05)	< 0.001	3.26 (2.82 to 3.77)	< 0.001	2.23
Phenotype 4 (High AF Chronicity) [reference]	1.00 (reference)	​	1.00 (reference)	​	1.00 (reference)	​	​
Phenotype 5 (Warfarin-Predominant Anticoagulation)	3.61 (3.22 to 4.05)	< 0.001	3.96 (3.53 to 4.45)	< 0.001	3.91 (3.48 to 4.39)	< 0.001	1.74
Phenotype 6 (Renal Dysfunction)	5.75 (5.17 to 6.39)	< 0.001	5.65 (5.08 to 6.29)	< 0.001	5.76 (5.17 to 6.42)	< 0.001	1.66
Age at first atrial fibrillation admission, per year	Not in model	​	1.040 (1.037 to 1.043)	< 0.001	1.042 (1.039 to 1.046)	< 0.001	1.46
Female sex	Not in model	​	1.007 (0.948 to 1.070)	0.814	1.045 (0.981 to 1.113)	0.172	1.17
CHA_2_DS_2_-VASc score, per point	Not in model	​	Not in model	​	0.955 (0.932 to 0.977)	< 0.001	2.37
Patients in model	13,967	​	13,967	​	13,967	​	​
Deaths	4,531	​	4,531	​	4,531	​	​
Concordance	0.663	​	0.699	​	0.701	​	​
Likelihood ratio test, chi square (df)	1729.2 (5)	​	2476.2 (7)	​	2491.3 (8)	​	​
Likelihood ratio test p value	< 0.001	​	< 0.001	​	< 0.001	​	​
Global proportional hazards test, chi square (df)	116.0 (5), p < 0.001	​	140.6 (7), p < 0.001	​	178.2 (8), p < 0.001	​	​
Maximum variance inflation factor	Not applicable	​	1.95	​	2.37	​	​

HR, hazard ratio; CI, confidence interval. Phenotype 4 (High AF Chronicity) is the reference category; it was selected because it has the longest reverse Kaplan-Meier median follow-up of any phenotype (854.9 days, 95% CI 776.1 to 910.8). Model 2 is the primary adjusted model and adjusts only for age and sex, which is the adjustment set that minimizes overlap with the phenotype-defining features. Model 3 additionally adjusts for CHA_2_DS_2_-VASc and is reported as a secondary model: the composite CHA_2_DS_2_-VASc score and all eight of its component terms were supplied to the clustering model, so Model 3 conditions on variables that helped define the phenotypes, and its estimates should not be read as evidence of phenotype-specific risk independent of established scores; the CHA_2_DS_2_-VASc coefficient reverses direction between the score-only model and Model 3, which shows that in Model 3 the term no longer carries its clinical meaning. Variance inflation factors are reported for Model 3; values above 5 conventionally indicate moderate collinearity and values above 10 serious collinearity. Age and sex were also inputs to the clustering model, so Model 2 reduces rather than eliminates the overlap. The proportional hazards assumption was rejected in all three models (global Schoenfeld residual test p < 0.001), so each hazard ratio is an average of a time-varying hazard ratio over the observed follow-up; period-specific estimates are given in Supplementary Table S14.

**Figure 5.**
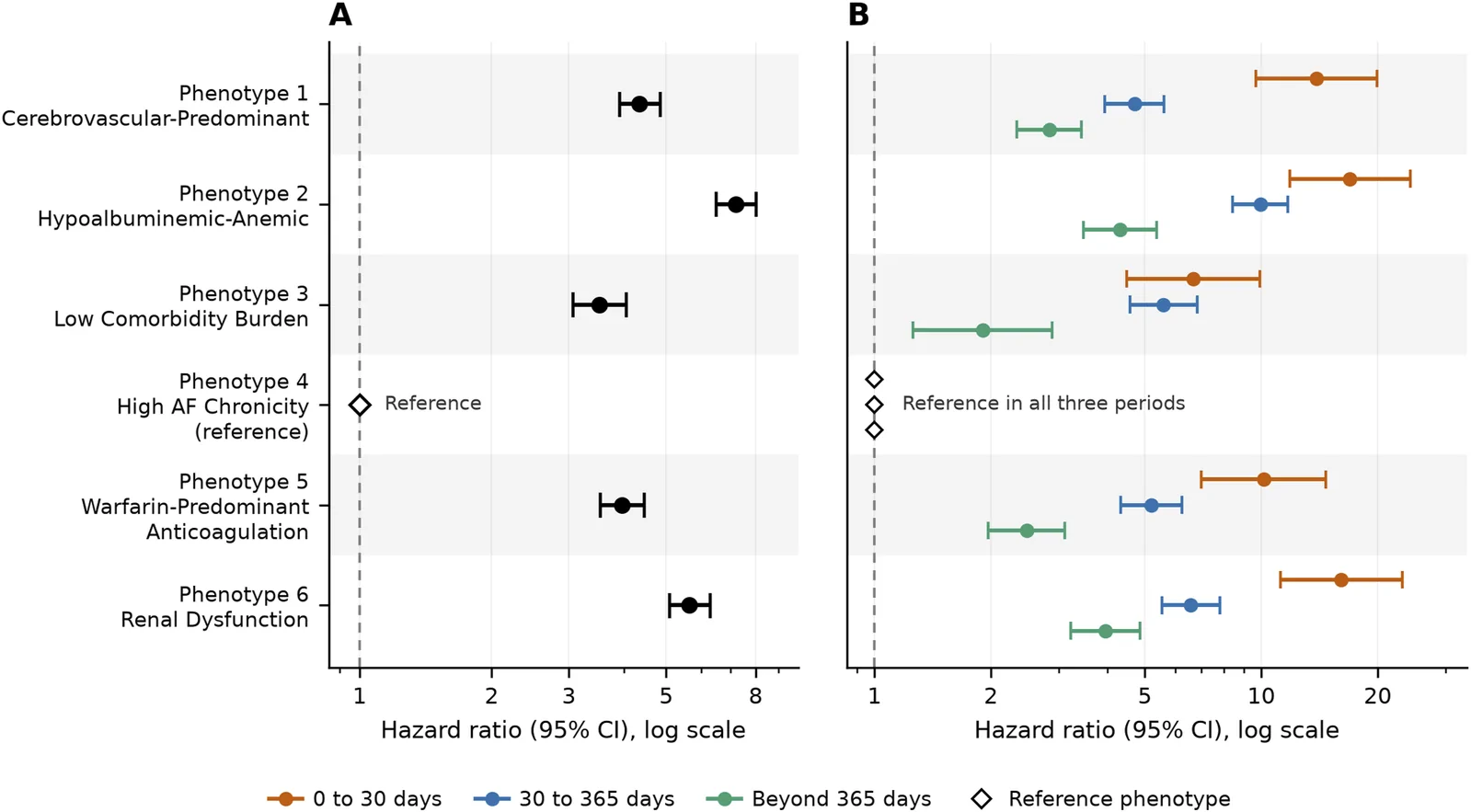
Hazard ratios for all-cause mortality by atrial fibrillation phenotype, with Phenotype 4 (High AF Chronicity) as the reference. (A) Pooled hazard ratios from the primary Cox proportional hazards model, adjusted for age at first atrial fibrillation admission and for sex, estimated over the whole of follow-up (13,967 patients, 4,531 deaths). (B) The same model refitted separately within three disjoint follow-up intervals: 0 to 30 days (1,541 deaths), 30 to 365 days (2,087 deaths), and beyond 365 days (903 deaths). Period-specific estimates are shown because the proportional hazards assumption was rejected in every model fitted (global Schoenfeld residual test chi square 140.6 on 7 degrees of freedom, p < 0.001, for the primary model), so each pooled hazard ratio in panel A is an average of a hazard ratio that falls as follow-up lengthens rather than a constant multiplier. Between the first 30 days and the period beyond 1 year the hazard ratios attenuate 3.5 to 4.9 fold. Phenotype 4 (High AF Chronicity) was chosen as the reference because it has the longest reverse Kaplan-Meier median follow-up of any phenotype (854.9 days, 95% CI 776.1 to 910.8); it is the best observed reference rather than an unbiased one, and its follow-up advantage is itself driven by health care utilization. Circles are point estimates and horizontal bars are 95% confidence intervals; open diamonds mark the reference phenotype, which is fixed at 1.00 by definition and has no confidence interval. Both x axes are logarithmic and the dashed vertical line marks a hazard ratio of 1.00; note that the two panels cover different ranges. Every hazard ratio shown differs significantly from the reference (p < 0.001, except Phenotype 3 (Low Comorbidity Burden) beyond 365 days, p = 0.002). Estimates additionally adjusted for the CHA_2_DS_2_-VASc score are reported as a secondary model in Table 2 and are deliberately not shown here, because the CHA_2_DS_2_-VASc score and all eight of its component terms were supplied to the clustering model; age and sex were also clustering inputs, so panel A reduces rather than eliminates that overlap. Numerical values for every estimate plotted are given in Table 2 (panel A) and in Supplementary Table S14 (panel B). Phenotypes: Phenotype 1 (Cerebrovascular-Predominant), n = 2,350; Phenotype 2 (Hypoalbuminemic-Anemic), n = 2,484; Phenotype 3 (Low Comorbidity Burden), n = 2,925; Phenotype 4 (High AF Chronicity), n = 2,124; Phenotype 5 (Warfarin-Predominant Anticoagulation), n = 2,178; Phenotype 6 (Renal Dysfunction), n = 1,906. CI, confidence interval

After adjustment for age and sex, phenotype remained associated with mortality (Table 2, Figure 5). Against Phenotype 4 (High AF Chronicity), adjusted hazard ratios ranged from 3.52 (95% CI 3.06 to 4.05) for Phenotype 3 (Low Comorbidity Burden) to 7.20 (95% CI 6.49 to 7.99) for Phenotype 2 (Hypoalbuminemic-Anemic), all p < 0.001. Adding CHA_2_DS_2_-VASc to the model changed no phenotype estimate by more than 7.4% and left the ordering unchanged, and every variance inflation factor in that model was below 2.5 (maximum 2.37 for CHA_2_DS_2_-VASc itself; largest phenotype indicator 2.23; Supplementary Table S15), so collinearity did not destabilize the estimates by conventional criteria. The CHA_2_DS_2_-VASc term nonetheless behaved incoherently once age was in the model, changing from a hazard ratio of 1.069 per point when fitted alone to 0.955 per point (95% CI 0.932 to 0.977) when fitted alongside age, sex, and phenotype, and it improved discrimination by only 0.002 (concordance 0.699 to 0.701). Adjusted estimates from the CHA_2_DS_2_-VASc model are therefore reported as a secondary analysis only and should not be read as evidence of phenotype-specific risk independent of established scores.

The proportional hazards assumption was rejected in all three models (global Schoenfeld residual test chi-square 116.0 on 5 degrees of freedom for the unadjusted model, 140.6 on 7 for the age and sex adjusted model, and 178.2 on 8 for the CHA_2_DS_2_-VASc adjusted model, all p < 0.001; Supplementary Table S16). Every reported hazard ratio is therefore an average of a relative hazard that falls as follow-up lengthens, not a constant multiplier. Refitting the primary model within disjoint follow-up intervals gave hazard ratios against Phenotype 4 of 6.67 to 16.96 in the first 30 days, 4.70 to 9.93 between 30 and 365 days, and 1.90 to 4.32 beyond 365 days, an attenuation of 3.5 to 4.9 fold (Figure 5, Supplementary Table S14). The rank ordering of the phenotypes was identical to the pooled model in the first and last intervals; in the 30 to 365 day interval Phenotype 1 (Cerebrovascular-Predominant) and Phenotype 3 (Low Comorbidity Burden) exchanged positions.

### Secondary Outcomes

Thirty-day readmission ranged from 13.0% in Phenotype 3 (Low Comorbidity Burden) to 42.1% in Phenotype 4 (High AF Chronicity) (chi-square = 744.0, p < 0.001). Readmission was not itself supplied to the clustering model, but it is derived from the same encounter record as the number of encounters, which was.

Intensive care admission ranged from 33.6% in Phenotype 3 (Low Comorbidity Burden) to 77.8% in Phenotype 2 (Hypoalbuminemic-Anemic) (chi-square = 1,647.6, p < 0.001), and cumulative length of stay differed markedly, with a median of 20.0 days in Phenotype 2 against 4.6 days in Phenotype 3, a 4.3 fold difference (Kruskal-Wallis H = 2,936.7, p < 0.001). Any intensive care admission and cumulative length of stay were themselves supplied to the clustering model, so these are descriptive characteristics of groups that were in part defined by utilization rather than independent outcomes. When the phenotypes were rebuilt without the utilization variables, the spread in intensive care admission fell from 44.2 to 25.1 percentage points, and the contrast between the baseline-only clusters that received the majority of Phenotype 2 and of Phenotype 3 narrowed from 4.3 fold to 2.0 fold (median 11.7 against 5.8 days). The overall gradient in length of stay was not diminished by that rebuild (4.4 fold across the baseline-only clusters against 4.3 fold in the primary partition). These sensitivity analyses are reported in full below.

### Follow-Up, Censoring, and Landmark Analyses

Median follow-up estimated by the reverse Kaplan-Meier method was 43.1 days (95% CI 38.0 to 50.5) overall and ranged from 5.3 days (95% CI 5.0 to 5.6) in Phenotype 3 (Low Comorbidity Burden) to 854.9 days (95% CI 776.1 to 910.8) in Phenotype 4 (High AF Chronicity). The number of patients at risk fell from 13,967 at time zero to 6,133 at 30 days and 2,389 at 365 days; at 365 days, 164 patients (5.6%) remained at risk in Phenotype 3 against 1,215 (57.2%) in Phenotype 4. Numbers at risk for every phenotype at 0, 30, 90, 180, 365, 730, and 1,095 days are given in Supplementary Table S11, and follow-up and censoring by phenotype in Supplementary Table S8.

Censoring was formally tested and was not independent of phenotype. The censoring distributions differed across phenotypes (reverse Kaplan-Meier log-rank chi-square 3,213.0 on 5 degrees of freedom, p < 0.001), and a Cox model of the censoring hazard on phenotype was highly significant (likelihood ratio test p < 0.001). Relative to Phenotype 4 (High AF Chronicity), Phenotype 3 (Low Comorbidity Burden) was censored 5.62 times faster (95% CI 5.24 to 6.02). Among censored patients, follow-up time was strongly rank-correlated with cumulative length of stay (Spearman rho = 0.73, p < 0.001) and with the number of encounters (Spearman rho = 0.64, p < 0.001). Censoring in this cohort is therefore informative and is driven by the same utilization variables that contribute to phenotype definition (Supplementary Table S12).

Two sensitivity analyses were performed to mitigate early censoring bias. In landmark analyses restricted to patients alive and under observation at 30 days (n = 6,103, 43.7%) and at 90 days (n = 4,560, 32.6%), mortality still differed across phenotypes (log-rank p < 0.001 at both landmarks), and hazard ratios against Phenotype 4 (High AF Chronicity) ranged from 2.71 to 5.16 at the 30-day landmark and from 2.77 to 4.67 at the 90-day landmark (Supplementary Table S13). When all patients were administratively censored at a common 1-year horizon, hazard ratios against Phenotype 4 ranged from 4.15 to 8.72, and the ordering of the phenotypes was preserved except that Phenotype 2 (Hypoalbuminemic-Anemic) and Phenotype 6 (Renal Dysfunction) exchanged adjacent positions, moving from 5.68 and 5.75 under full follow-up (Table 2) to 8.72 and 8.69 under administrative censoring. Those two phenotypes are the single pair whose mortality did not differ significantly from each other (p = 0.71), and their two censored estimates differ by 0.03, so the exchange is not a substantive reordering.

### Incremental Discrimination Beyond Routine Variables

Discrimination for all-cause mortality was compared across nested Cox models (Supplementary Figure S10, Supplementary Table S17). CHA_2_DS_2_-VASc alone discriminated poorly (Harrell C = 0.533, 95% CI 0.522 to 0.543), which is expected for a score developed to estimate stroke risk rather than death. Adding age and sex raised the C-index to 0.603 (0.594 to 0.613), and adding three routinely measured laboratory values (creatinine, hemoglobin, and albumin) raised it further to 0.683 (0.673 to 0.692), an increment of 0.079 (0.069 to 0.089; likelihood ratio 1,256.7 on 3 degrees of freedom, p < 0.001). Adding the phenotype to this model raised the C-index to 0.733 (0.725 to 0.742), an increment of 0.051 (0.046 to 0.056; likelihood ratio 1,244.5 on 5 degrees of freedom, p < 0.001). Optimism estimated by a 500-resample bootstrap was negligible for every model (at most 0.0008 C-index units), so optimism-corrected values were essentially unchanged (0.533, 0.603, 0.682, and 0.732 respectively). Time-dependent areas under the curve at 30, 90, and 365 days were 0.506, 0.503, and 0.506 for CHA_2_DS_2_-VASc alone and 0.747, 0.774, and 0.830 for the model containing the phenotype.

The size of the phenotype’s contribution depended on what it was compared against. Added to age, sex, and CHA_2_DS_2_-VASc, the phenotype raised the C-index by 0.098 (0.090 to 0.104); added to the same model already containing the three laboratory values, by 0.051 (0.046 to 0.056); and added to a model that also contained encounter count, any intensive care admission, and total length of stay, by 0.022 (0.018 to 0.025). Approximately half of the phenotype’s apparent incremental discrimination over a CHA_2_DS_2_-VASc-based model is therefore attributable to information carried by three routine laboratory results. A conventional Cox model containing age, sex, CHA_2_DS_2_-VASc, the three laboratory values, and the three utilization variables, with no phenotype term, reached a C-index of 0.745 (0.737 to 0.754), which exceeded the model containing the phenotype but not those utilization variables (0.733, 0.725 to 0.742).

Because the phenotypes were derived using variables that also contribute to CHA_2_DS_2_-VASc, discrimination was re-estimated under cross-validation in which the standardizer, the variational autoencoder, and the k-means solution were all refitted inside each training fold, with validation patients assigned to the nearest training-fold centroid in that fold’s latent space (three repeats of stratified five-fold cross-validation, giving 15 independent refits of the representation and the clustering). Cross-validated C-indices were 0.533 (0.528 to 0.538) for CHA_2_DS_2_-VASc alone, 0.603 (0.598 to 0.608) after adding age and sex, 0.682 (0.676 to 0.688) after adding the three laboratory values, and 0.741 (0.736 to 0.747) after adding the phenotype, an increment of 0.059 (0.056 to 0.062, paired across folds, p < 0.001). Repeating the same folds while assigning held-out patients the phenotype labels derived from the full cohort gave a lower cross-validated C-index (0.732, 0.726 to 0.739), so the phenotype’s contribution did not depend on the clustering having seen the held-out patients.

### Hazard Rate Vs. Cumulative Mortality Paradox

Observed-death proportions and hazard ratios point in opposite directions for the phenotypes with the shortest observation. Phenotype 3 (Low Comorbidity Burden) had the lowest observed-death proportion of the six (11.1%) yet an unadjusted hazard ratio of 2.64 (95% CI 2.30 to 3.04) against Phenotype 4 (High AF Chronicity), whose observed-death proportion was 29.1%. Two features of the data produce this pattern. First, the observation windows differ by more than two orders of magnitude between these two phenotypes (reverse Kaplan-Meier median follow-up 5.3 days against 854.9 days), so the two proportions do not accrue over comparable time. Second, the relative hazard is not constant: refitting the primary model within disjoint follow-up intervals showed that hazard ratios against Phenotype 4 attenuate 3.5 to 4.9 fold between the first 30 days and the period beyond 1 year (Figure 5), so phenotypes observed only briefly are compared on the steep early portion of the hazard curve.

One-year mortality is only weakly identified for the phenotypes with short follow-up. For Phenotype 3 (Low Comorbidity Burden), 10.2% of patients were observed to die within 365 days, the Kaplan-Meier estimate of 1-year mortality was 51.2%, and the interval consistent with the observed data spans 10.2% to 94.4%. For Phenotype 4 (High AF Chronicity), the corresponding figures were 10.4%, 14.0%, and 10.4% to 42.8%. The all-cause mortality proportions reported by phenotype are therefore observed-death proportions accrued over markedly different observation windows and should not be read as 1-year risks (Supplementary Table S8).

### Treatment Patterns

**Figure 6.**
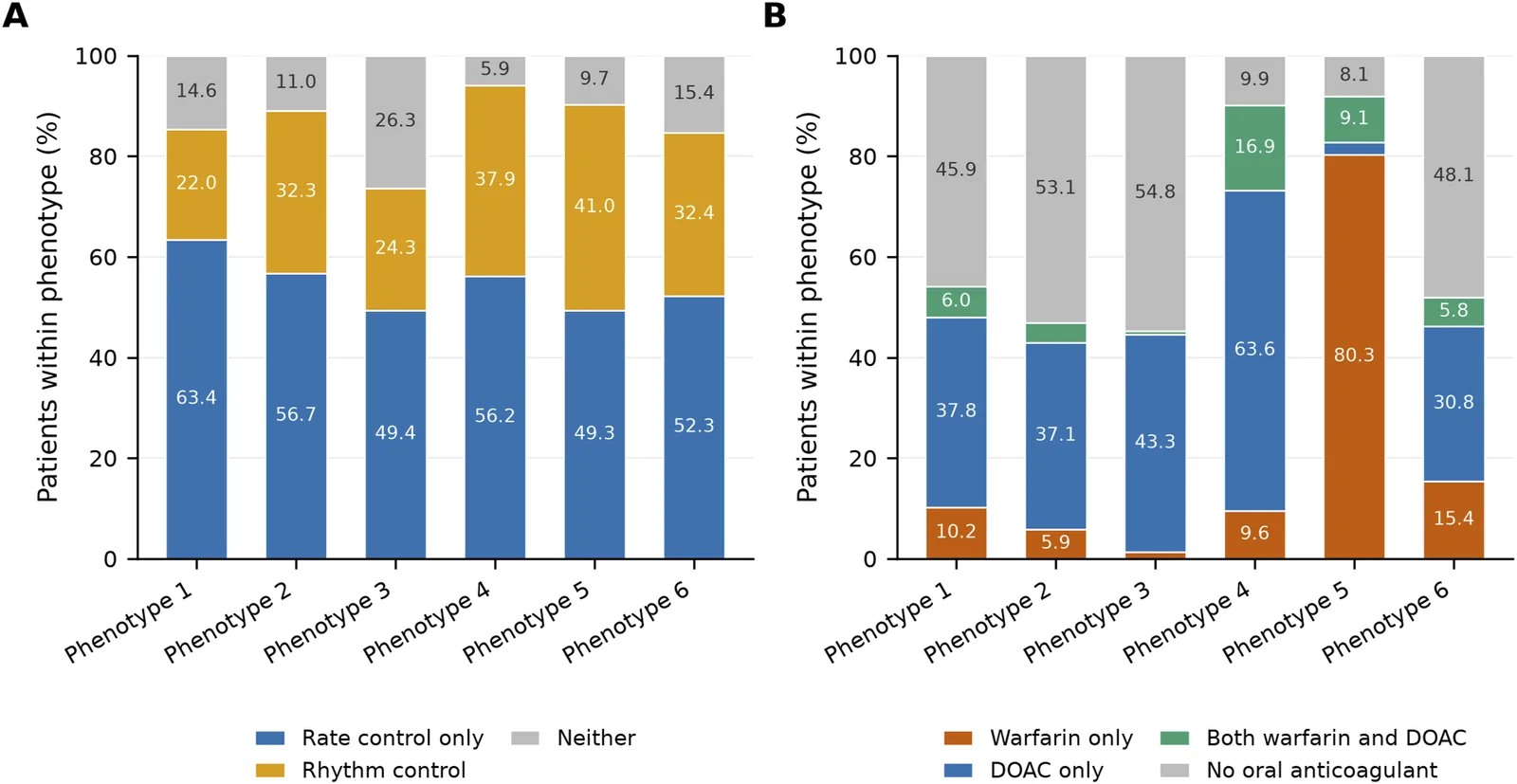
Treatment patterns by atrial fibrillation phenotype. (A) Rate versus rhythm control. Rate control only denotes a patient prescribed a beta blocker, a calcium channel blocker, or digoxin and no antiarrhythmic drug; rhythm control denotes a patient prescribed any antiarrhythmic drug, alone or together with a rate-control agent; neither denotes a patient prescribed no rate-control agent and no antiarrhythmic drug. (B) Anticoagulation pattern, expressed as four mutually exclusive categories: warfarin only, direct oral anticoagulant (DOAC) only, both warfarin and a DOAC, and no oral anticoagulant. A patient prescribed both warfarin and a DOAC at any point in the record is counted once in the both category rather than counted separately in the warfarin and DOAC categories. Within each panel the categories are mutually exclusive and exhaustive, so bars sum to 100% of the patients in that phenotype; the y axis is bounded at 100%. Medication variables are patient-level ever-prescribed flags spanning the full MIMIC-IV record rather than index-admission exposures. Phenotypes: Phenotype 1 (Cerebrovascular-Predominant), n = 2,350; Phenotype 2 (Hypoalbuminemic-Anemic), n = 2,484; Phenotype 3 (Low Comorbidity Burden), n = 2,925; Phenotype 4 (High AF Chronicity), n = 2,124; Phenotype 5 (Warfarin-Predominant Anticoagulation), n = 2,178; Phenotype 6 (Renal Dysfunction), n = 1,906. Segments smaller than 5% are not labeled within the bars. Percentages are rounded to one decimal place and may not add to exactly 100. DOAC, direct oral anticoagulant

Treatment patterns differed across phenotypes (Figure 6; chi-square testing, p < 0.001). Rate control without an antiarrhythmic agent was the most common pattern in every phenotype, ranging from 49.3% in Phenotype 5 (Warfarin-Predominant Anticoagulation) to 63.4% in Phenotype 1 (Cerebrovascular-Predominant). Rhythm control ranged from 22.0% in Phenotype 1 to 41.0% in Phenotype 5, and the proportion receiving neither a rate-control agent nor an antiarrhythmic ranged from 5.9% in Phenotype 4 (High AF Chronicity) to 26.3% in Phenotype 3 (Low Comorbidity Burden).

Anticoagulation was summarized as four mutually exclusive categories. Warfarin without a direct oral anticoagulant ranged from 1.3% in Phenotype 3 (Low Comorbidity Burden) to 80.3% in Phenotype 5 (Warfarin-Predominant Anticoagulation); a direct oral anticoagulant without warfarin from 2.4% in Phenotype 5 to 63.6% in Phenotype 4 (High AF Chronicity); exposure to both agents at some point in the record from 0.6% in Phenotype 3 to 16.9% in Phenotype 4; and no recorded oral anticoagulant from 8.1% in Phenotype 5 to 54.8% in Phenotype 3. Because medication variables are cumulative ever-prescribed flags across the whole record, the category of exposure to both agents indicates switching or sequential exposure rather than concurrent dual anticoagulation. Supplementary Table S6 reports the same patients under the overlapping ever-exposed definitions of warfarin and direct oral anticoagulant use, in which a patient exposed to both agents is counted in both columns, so those percentages are higher than the mutually exclusive ones given here.

Anticoagulant exposure also varied by anchor year group (Supplementary Figure S7, Supplementary Table S21). Across the 2014 to 2016, 2017 to 2019, and 2020 to 2022 groups, warfarin exposure fell from 37.3% to 26.4% to 14.4% (Cochran-Armitage z = -24.18, p < 0.001) while direct oral anticoagulant exposure rose from 38.0% to 41.7% to 48.9% (z = +10.18, p < 0.001). Within Phenotype 5 (Warfarin-Predominant Anticoagulation), warfarin exposure fell from 93.9% to 89.8% to 81.5% (z = -6.83, p < 0.001), and the phenotype accounted for a falling share of each successive group, 19.6%, 16.4%, and 10.9% (z = -11.11, p < 0.001).

### Validation

#### Temporal Split Validation

Temporal validation did not meet the pre-specified ±5% distribution threshold (maximum difference 9.0%). After Hungarian matching (mean cosine similarity 0.891, range 0.821 to 0.955), mortality concordance was reasonable (0.0 to 8.8 percentage point differences), suggesting that outcome associations are more temporally stable than cluster sizes (Supplementary Figure S5A, Supplementary Table S5).

#### Bootstrap Stability

Bootstrap stability was assessed at two levels. Holding the trained variational autoencoder fixed and refitting K-means on 100 subsamples of 80% of the cohort, agreement with the primary partition was high: pair-counting Jaccard 0.926 (SD 0.018), Adjusted Rand Index 0.954 (SD 0.012), and per-cluster matched Jaccard 0.961 (SD 0.010; 0.948 to 0.976 across the six phenotypes), with a Proportion of Ambiguous Clustering of 0.040 (Supplementary Figure S5B). An end-to-end bootstrap that also retrained the variational autoencoder from scratch on each subsample gave lower agreement, as expected when the latent axes are re-estimated: pair-counting Jaccard 0.343 (SD 0.045), Adjusted Rand Index 0.406 (SD 0.060), matched Jaccard 0.516 (SD 0.056), and a Proportion of Ambiguous Clustering of 0.352. The identical end-to-end pipeline applied to a permuted dataset, in which each feature column was independently shuffled to preserve marginal distributions while destroying joint structure, still yielded an Adjusted Rand Index of 0.199 (SD 0.058) and a matched Jaccard of 0.378 (SD 0.060), indicating that the analytic chance constant of 0.091 substantially understates the agreement this pipeline produces when no structure is present. Outcome-level stability exceeded assignment-level stability: the range in phenotype-specific observed-death proportions was 39.2 percentage points across end-to-end replicates (95% interval 34.6 to 45.2), compared with 37.0 points in the primary solution and 2.1 points (0.9 to 3.8) under the permuted null (Supplementary Figure S8, Supplementary Table S10).

Reproducibility was not uniform across phenotypes. Under the end-to-end variant, per-phenotype matched Jaccard values were 0.533 for Phenotype 1 (Cerebrovascular-Predominant), 0.614 for Phenotype 2 (Hypoalbuminemic-Anemic), 0.563 for Phenotype 3 (Low Comorbidity Burden), 0.507 for Phenotype 4 (High AF Chronicity), 0.435 for Phenotype 5 (Warfarin-Predominant Anticoagulation), and 0.446 for Phenotype 6 (Renal Dysfunction), against a permuted-null mean of 0.378; the 95% intervals for Phenotypes 5 and 6 extended well below that null mean. Those two phenotypes were also the pair most often assigned together in the end-to-end consensus matrix (co-assignment 0.207, more than double the next highest pair, 0.153 for Phenotypes 1 and 4). What reproduced was the size of the mortality gradient rather than the position of individual phenotypes along it: the gradient never fell below 18.8 percentage points in any of the 100 end-to-end replicates while it never exceeded 4.4 points under the permuted null, but rank concordance of phenotype mortality with the primary solution was Spearman 0.618, the complete rank order was reproduced in 16% of replicates, and the highest-mortality phenotype was recovered in 36% of replicates against 88% for the lowest (Supplementary Table S10).

#### Sensitivity Analyses

K-sensitivity analysis showed silhouette scores from 0.1213 at k=4 to 0.1386 at k=6, a total spread of 0.017, and the four internal validity indices did not converge on a common solution (Supplementary Table S9, Supplementary Figure S9). Bootstrap reproducibility was high at every value tested, with adjusted Rand indices of 0.889 at k=4, 0.930 at k=5, 0.955 at k=6, 0.926 at k=7, and 0.904 at k=8. The key mortality findings held qualitatively across solutions: a low-mortality cluster (11.6% at k=4, 11.1% at k=6, 8.7% at k=8) and a high-mortality cluster (48.1%, 48.1%, and 53.5%, respectively) were present at every k. Reducing to k=4 dispersed the Phenotype 4 (High AF Chronicity) and Phenotype 6 (Renal Dysfunction) profiles, only 39.0% and 57.2% of whose members reached any single k=4 cluster, and absorbed both into a composite cluster of 3,906 patients whose 35.1% mortality lies between the 27.9% to 48.1% range of the phenotypes it combines. Increasing to k=8 subdivided rather than replaced the six profiles, in that seven of the eight k=8 clusters drew the majority of their members from a single k=6 phenotype. Phenotype 2 (Hypoalbuminemic-Anemic) sent 67.9% of its 2,484 patients to one k=8 cluster of 2,536 patients with 45.5% mortality, supplying 66.5% of that cluster, and a further 24.5% to a high-utilization cluster of 936 patients with 53.5% mortality, supplying 65.1% of that cluster. Phenotype 5 (Warfarin-Predominant Anticoagulation) sent 54.3% of its 2,178 patients to a high-INR cluster of 1,239 patients with 39.1% mortality, supplying 95.4% of that cluster, and a further 38.3% to the one k=8 cluster that was not dominated by any single phenotype, a group of 2,164 patients with 13.0% mortality that took 38.6% of its members from Phenotype 5 and the remainder from the other five. The two additional clusters therefore separated utilization intensity and anticoagulation intensity within profiles that already existed at k=6 rather than identifying new ones.

Latent dimension sensitivity (dimensions 8, 10, 12) yielded silhouette scores 0.1320 to 0.1632, indicating moderate robustness (Supplementary Figure S5C, Supplementary Figure S5D). The feature correlation matrix is presented in Supplementary Figure S6.

When the three healthcare utilization variables were removed and the model was retrained, all six phenotypes reappeared with their defining standardized profiles: a cluster with higher age, higher CHA_2_DS_2_-VASc score and more prior stroke (z = +0.67, +0.64 and +0.57); a cluster with low comorbidity burden (CHA_2_DS_2_-VASc z = -1.00); a warfarin predominant cluster (warfarin z = +1.42, international normalized ratio z = +1.12); a hypoalbuminemic and anemic cluster (albumin z = -1.03, hemoglobin z = -0.64); a renal cluster (creatinine z = +1.32, chronic kidney disease z = +1.05); and a high chronicity cluster (AF coded admission interval z = +2.17, AF encounters z = +1.74). Agreement with the primary partition was moderate (adjusted Rand index 0.438, SD 0.054 across ten refits; adjusted mutual information 0.449, SD 0.042). This is not lower than the agreement obtained when the unchanged 35 variable feature set is refit ten times with the training and validation split held fixed and only the network initialization varied (adjusted Rand index 0.400, SD 0.070; Mann-Whitney p = 0.104). Refitting the same unchanged feature set while allowing the split to vary as well gave an adjusted Rand index of 0.432 over five refits (range 0.384 to 0.513), and an end-to-end bootstrap that additionally resampled patients gave 0.406, so refitting this pipeline on unchanged data reproduces the published partition at an adjusted Rand index of approximately 0.40 to 0.43 depending on which sources of randomness are held fixed. The disagreement observed after removing the utilization variables therefore reflects instability of the learned representation under refitting rather than loss of those variables. Between 55.4% and 86.6% of the patients in each published phenotype were assigned to a single baseline-only cluster (Supplementary Figure S11, Supplementary Table S18).

Because healthcare utilization no longer contributed to cluster definition, length of stay and intensive care admission became outcomes rather than inputs in that solution. Both still differed across the baseline-only clusters. Median cumulative length of stay ranged from 5.8 to 25.6 days (Kruskal-Wallis p < 0.001) and intensive care admission from 48.2% to 73.3% (p < 0.001). Observed-death proportions ranged from 8.5% to 48.4%, a gradient of 39.9 percentage points against 37.0 percentage points in the primary partition, with a global log-rank chi-square of 2,604.7 on 5 degrees of freedom (p < 0.001) and unadjusted hazard ratios of 0.42 to 4.98 relative to BO1-B, the cluster with the lowest observed mortality. The concordance of the unadjusted Cox model was 0.698, higher than the 0.663 obtained for the published phenotypes. In the stricter second variant, which also removed the two encounter derived atrial fibrillation variables, the mortality gradient was 43.3 percentage points and the global log-rank chi-square was 909.1 (p < 0.001).

Two aspects of the original comparison were nonetheless affected. The spread in intensive care admission narrowed from 44.2 percentage points across the published phenotypes to 25.1 percentage points across the baseline-only clusters and to 18.2 percentage points when the encounter derived variables were also removed, so roughly half of the apparent separation in intensive care use was attributable to including that variable in the model. The ordering of length of stay also changed: median cumulative stay in Phenotype 2 (Hypoalbuminemic-Anemic) was 20.0 days in the primary partition against 4.6 days in Phenotype 3 (Low Comorbidity Burden), a 4.3 fold difference, whereas in the baseline-only partition the clusters that received the majority of those two phenotypes, BO1-D and BO1-B, differed by 11.7 against 5.8 days, a 2.0 fold difference. Length of stay and intensive care use therefore do differ by phenotype, but the magnitude of those differences in the primary analysis was inflated by the inclusion of utilization variables among the clustering features.

Phenotype 4 (High AF Chronicity) was the one component of the solution that depended on encounter derived variables. In the first variant it was recovered as a distinct cluster (55.4% of its patients assigned to a single cluster, with the AF coded admission interval at z = +2.17). In the stricter second variant, in which that interval and the count of AF coded encounters were both removed, it no longer emerged: its patients distributed across four clusters, the largest receiving 36.7%, and a cluster defined by obesity and obstructive sleep apnea (z = +1.08 and +0.96) appeared in its place. This phenotype should therefore be interpreted as a marker of repeated coded contact with the health system rather than as an independently measured disease duration.

Recomputing every feature at the index admission alone reduced the prevalence of the 18 comorbidity and medication flags by a mean of 5.0 percentage points (median 4.9, maximum 9.2 for angiotensin converting enzyme inhibitors or angiotensin receptor blockers), a mean relative inflation of 1.22 for the cumulative encoding. Chronic kidney disease fell from 30.0% (4,185 patients) to 25.1% (3,499), so 686 patients (16.4% of those ever coded) carried the diagnosis only from a non-index admission. The inflation was entirely a function of record length: among the 6,927 patients with a single admission the two encodings were identical for all 18 flags, whereas among the 7,040 patients with more than one admission the cumulative encoding raised prevalence by a mean of 9.8 percentage points. Retraining the model on the 28 index-admission features yielded a six-cluster solution whose agreement with the primary partition was an adjusted Rand index of 0.148 (0.159 averaged over five random initializations) and an adjusted mutual information of 0.187. That value should be read against the agreement expected when the model is refit at all rather than against complete agreement: refitting on the unchanged 35 variable feature set, varying the random initialization and the training and validation split across five refits, already gave a mean adjusted Rand index of 0.432 (range 0.384 to 0.513). Of the 0.273 drop from that refit baseline, 0.193 was attributable to dropping the seven features without index-admission counterparts and 0.080 to re-encoding the remainder. Isolating the encoding change alone, by comparing the index-admission solution with a refit of the same 28 features under cumulative encoding at matched seeds, gave an adjusted Rand index of 0.291, against a matched within-arm control in which two refits of that same cumulative specification agreed at an adjusted Rand index of 0.533, so the change of encoding moved the partition further than refitting alone does (Supplementary Figure S12, Supplementary Tables S19 and S20). The mortality gradient was preserved: observed-death proportions ranged 36.2 percentage points across the six index-admission clusters, against 37.0 percentage points in the primary solution.

## Discussion

This study identified six AF phenotypes using an approach that combined variational autoencoder based dimensionality reduction with unsupervised clustering. The phenotypes differed substantially in all-cause mortality, with observed-death proportions ranging from 11.1% to 48.1% over markedly unequal observation windows, and in 30-day readmission, which ranged from 13.0% to 42.1%. They also differed in intensive care utilization, which ranged from 33.6% to 77.8%, and in treatment patterns. Because intensive care admission and cumulative length of stay were themselves supplied to the clustering model, those two contrasts describe the phenotypes rather than serving as independent outcomes, and the intensive care spread falls from 44.2 to 25.1 percentage points when the phenotypes are rebuilt without utilization variables. Formal measures of cluster separation were modest. Even so, the phenotypes showed coherent clinical profiles, and a between-phenotype mortality gradient was present in every end-to-end resample of the model while being an order of magnitude smaller when the joint structure of the data was destroyed. Taken together, these findings suggest that the approach captures real heterogeneity within this AF population that is not fully reflected in current classification schemes.

The main methodological contribution of this work is the use of variational autoencoders for AF phenotyping. To our knowledge, this is the first study to apply VAEs to comprehensive clinical phenotype discovery in an AF population. Previous AF phenotyping studies have generally used more traditional clustering methods, including K-means, K-prototype, and hierarchical clustering, either on raw clinical features or on linearly transformed features derived from principal component analysis.^5-9^ Those approaches have identified clinically relevant subgroups, but they are limited in their ability to capture nonlinear relationships among features. VAEs learn probabilistic, nonlinear latent representations through deep neural networks and may therefore detect complex feature interactions that linear methods miss.^10-12,14,15^ In that context, the six-phenotype solution identified here separates a cerebrovascular-predominant group from a group defined by renal dysfunction, and it isolates a group defined by warfarin-predominant anticoagulation that a duration-based classification would not distinguish.

The clinical profiles of these phenotypes both align with and extend the prior AF phenotyping literature, and the extent of their novelty should be stated plainly. Most of the six correspond to combinations of comorbidity burden, illness acuity, anticoagulation pattern, or coded AF chronicity that are already familiar from conventional clinical characterization, and they are not new disease entities. Phenotype 1 (Cerebrovascular-Predominant), Phenotype 6 (Renal Dysfunction), and Phenotype 3 (Low Comorbidity Burden) in particular reproduce groupings that CHA_2_DS_2_-VASc and routine multimorbidity assessment would identify without any clustering: the CHA_2_DS_2_-VASc score correlates with membership of Phenotype 1 at r = +0.45 and of Phenotype 3 at r = -0.44, and with age at r = +0.55. What the analysis adds is therefore not novel biology but a single framework in which these co-occurrence patterns and their outcome gradients are estimated together and can be inspected feature by feature. Phenotype 3 (Low Comorbidity Burden), marked by younger age and a low comorbidity burden, resembles in its feature profile the young clusters reported in ORBIT-AF and other registries,^5,6,20,21^ but it cannot be described as low risk on these data: it has the shortest reverse Kaplan-Meier follow-up of the six phenotypes at 5.3 days, and the interval for 1-year mortality that these data identify runs from 10.2% to 94.4%, so whether it is a candidate for the kind of early rhythm control strategy tested in EAST-AFNET 4 cannot be judged from this cohort.^1,5,6,20,21^ At the other end of the spectrum, Phenotype 2 (Hypoalbuminemic-Anemic) and Phenotype 6 (Renal Dysfunction), with observed-death proportions of 47.5% and 48.1%, represent high-burden subgroups that would be expected to require integrated, multi-organ management. Phenotype 4 (High AF Chronicity) was characterized by a long AF coded admission interval (z = +1.34) and a high 30-day readmission rate (42.1%) despite an intermediate observed-death proportion of 29.1%. That pattern is consistent with a chronic cycle of healthcare utilization, and whether catheter ablation aimed at interrupting AF progression could reduce recurrent hospitalization in such a group is a hypothesis that would require prospective testing.^3,22^

The dependence of the phenotypes on established risk scores can be stated precisely. The composite CHA_2_DS_2_-VASc score was one of the features supplied to the variational autoencoder, and all eight of its component terms, spanning heart failure, hypertension, age, diabetes, prior stroke or transient ischemic attack, vascular disease, and female sex, were also supplied. Comparing phenotypes after adjusting for CHA_2_DS_2_-VASc therefore cannot establish that phenotype carries information the score does not, and no such claim is made. In the model containing age, sex, three routine laboratory values, and the phenotype, the CHA_2_DS_2_-VASc coefficient reversed direction (hazard ratio 0.92 per point, 95% CI 0.89 to 0.94), which is a collinearity artifact and must not be read as a clinical finding. Inference about the phenotypes therefore rests on discrimination rather than on adjusted hazard ratios for the score.

These results place a boundary on what the phenotypes contribute. They discriminate mortality beyond age, sex, CHA_2_DS_2_-VASc, and routine laboratory values, and that increment persists after optimism correction and after cross-validation in which the representation and the clustering were re-derived within each training fold. They do not, however, outperform a conventional regression fitted directly to the same underlying variables. The clustering compresses a continuous multivariable risk surface into six ordered strata, and that compression costs discrimination. The value of the phenotypes is therefore not that they predict death better than a regression can, but that they summarize a high-dimensional clinical profile into a small number of interpretable strata whose composition can be inspected, described, and compared across settings. Any claim of predictive superiority over conventional modeling would be unsupported by these data. The weak performance of CHA_2_DS_2_-VASc alone in this comparison (concordance index 0.533) should not be read as a failure of the score: it was developed to estimate stroke risk rather than death, and near-chance discrimination for all-cause mortality in a critically ill cohort is the expected result.

The phenotype defined by warfarin-predominant anticoagulation has potential quality improvement implications, and it also carries the clearest evidence in this study that a phenotype can be shaped by how treatment is recorded rather than by a clinical state. Phenotype 5 (Warfarin-Predominant Anticoagulation) was marked by high warfarin exposure (z = +1.46) and an elevated international normalized ratio (z = +1.19) together with low direct oral anticoagulant exposure (z = -0.63), and it remained warfarin-dominant after anticoagulation was recoded into mutually exclusive categories. There was little corresponding increase in guideline-concordant reasons to remain on warfarin, such as valvular heart disease (z = +0.18) or severe chronic kidney disease (z = +0.04) (Figure 3), and the elevated international normalized ratio suggests active therapeutic monitoring rather than treatment abandonment.

Warfarin exposure in this cohort was concentrated in the earlier anchor year groups, both overall and within Phenotype 5, and the phenotype accounted for a falling share of each successive group. Because medication variables are cumulative ever-exposed flags and record length falls sharply across these groups, part of that decline reflects differing opportunity to accumulate exposure; the same direction was present, at lower absolute levels, among the 6,927 patients with a single admission, in whom the flag reduces to index-encounter exposure (warfarin 29.5%, 24.0%, and 12.9%; direct oral anticoagulant 20.1%, 30.1%, and 43.1%; both p < 0.001). Warfarin exposure nonetheless remained at 81.5% within the phenotype in the most recent group, so this is a shrinking but persistent group rather than a purely historical one. These observations are consistent with the hypothesis of clinical inertia, that some patients started on warfarin before direct oral anticoagulants were established were never transitioned, but they do not establish it. The contraindication case mix that would justify continued warfarin, including mechanical valves, moderate to severe mitral stenosis, and severe renal impairment, cannot be resolved from the available features, and the anchor year group is a de-identified three-year window rather than a prescription date. Whether phenotype-guided anticoagulation review improves outcomes would require prospective evaluation and is not demonstrated by this analysis.^1^

Two further cautions attach to this phenotype. The proportion of patients ever prescribed both warfarin and a direct oral anticoagulant ranged from 0.6% in Phenotype 3 (Low Comorbidity Burden) to 16.9% in Phenotype 4 (High AF Chronicity); because the medication variables are cumulative over the whole record, this indicates switching or sequential exposure rather than concurrent dual anticoagulation. More importantly, Phenotype 5 was the least reproducible of the six phenotypes when the model was refit end to end (matched Jaccard 0.435) and again when every feature was recomputed at the index admission (matched Jaccard 0.141), yet it was the best preserved of the six when clustering features were removed, with 86.6% of its members held together in a single baseline-only cluster. A group that survives the removal of other variables but dissolves when the measurement window for its own defining variable changes is substantially an artifact of a cumulative treatment flag. That is also why the HAS-BLED implementation used here matters: it substitutes a warfarin term for the labile international normalized ratio component, so the same variable that defines Phenotype 5 also loads into a score that helps separate Phenotype 6 (Renal Dysfunction), and those two phenotypes are the pair most often merged when the model is refit.

Encoding comorbidities and medications as ever present across the whole record inflated their prevalence by a mean of 5.0 percentage points, entirely because patients with longer records accumulate more opportunities to be coded; among patients with a single admission the cumulative and index-admission encodings were identical. Because record length falls sharply across the anchor year groups, from a mean of 3.47 encounters in 2014 to 2016 to 1.59 in 2020 to 2022, this encoding also mixes calendar era into apparent disease burden. The two laboratory values with an index-admission equivalent move in the opposite direction from the diagnosis flags: median creatinine was 1.254 across the whole record against 1.370 at the index admission, and median hemoglobin 10.762 against 11.206, so both are higher at the index admission than in the whole-record median. Cumulative encoding also raises the mean CHA_2_DS_2_-VASc score by 0.29 points and the mean HAS-BLED score by 0.43 points relative to their index-admission values.

The effect on phenotype composition was not uniform. Phenotype 4 (High AF Chronicity) was the most affected, with a mean absolute change in feature prevalence of 10.8 percentage points and a halving of the proportion of its members coded longstanding persistent or permanent AF, from 19.0% to 9.7%. Phenotype 6 (Renal Dysfunction), by contrast, was the second most robust of the six, retaining a matched Jaccard of 0.347 against the published partition, behind only Phenotype 1 (Cerebrovascular-Predominant) at 0.490; its chronic kidney disease prevalence fell only from 79.2% to 74.8%, because the phenotype is anchored by creatinine measured at the index admission rather than by a cumulative diagnosis flag, and a renal cluster with the highest observed-death proportion of the six re-emerged under index-admission encoding. The phenotypes most sensitive to the change were instead Phenotype 5 (Warfarin-Predominant Anticoagulation), which is defined by a cumulative medication flag and retained a matched Jaccard of only 0.141, and Phenotype 3 (Low Comorbidity Burden), which is defined by the absence of comorbidity and is therefore the phenotype most exposed to patients losing flags (0.210).

The two re-specification analyses reached different verdicts, and they should not be summarized as a single statement about robustness. Removing the three healthcare utilization variables did not move the partition further than refitting the model on unchanged data already does, so the circularity in the utilization comparisons, while real and conceded above for intensive care admission and length of stay, did not change which patients group together. Recomputing every feature at the index admission did move the partition, and by a margin larger than refitting alone produces. The practical implication is that the phenotypes reported here are properties of a cumulative, whole-record representation of a patient, and a version intended for use within a single admission would have to be derived that way from the outset rather than transferred from this one.

These phenotypes were derived in a single-center critical care database, and that constrains what they describe. MIMIC-IV captures the inpatient record of one tertiary academic hospital, the cohort was assembled from hospital admissions carrying an AF diagnosis code, 59.3% of patients had at least one intensive care admission, and the overall observed-death proportion was 32.4%. That is a selected, acutely ill population, and it differs from the ambulatory and registry AF populations in which precision management strategies are usually contemplated. The consequences are concrete rather than generic. Phenotype 2 (Hypoalbuminemic-Anemic) is defined by markers of acute illness that would be far less prevalent, and far less discriminating, in an outpatient cohort. Phenotype 3 (Low Comorbidity Burden) is in part a group of brief admissions, and its 5.3-day reverse Kaplan-Meier median follow-up reflects the hospital record rather than the patients’ clinical course. MIMIC-IV contains no outpatient encounters, so AF managed entirely in clinic is invisible here, and both the prevalence and the composition of the phenotypes would be expected to differ in a population that includes it. Temporal validation within the same institution did not reproduce the phenotype distribution to within the pre-specified ±5 percentage point threshold (maximum difference 9.0%), which is a further reason to expect distributional shift across settings. These phenotypes should therefore be read as a description of hospitalized AF in one critical care system, and they should not be extrapolated to ambulatory or registry-based AF populations without derivation and validation in those settings.

This study does not establish a clinical application for these phenotypes, and it is worth stating concretely what would have to be true before a phenotype assignment could influence care. Four conditions would need to be met. First, the assignment would have to be reproducible for an individual patient, which it is not in these data: refitting the model end to end reproduced individual assignments at an Adjusted Rand Index of 0.406, and two of the six phenotypes were recovered only marginally above an empirical null. A deployable version would need a fixed, published encoder and a fixed set of centroids, so that assignment becomes a deterministic function of a patient’s record rather than the output of a model that is refit. Second, the assignment would have to be computable at the moment a decision is made. Cumulative encoding is pervasive here rather than incidental: 33 of the 35 features are computed over the whole record, only sex and age at the first AF admission being fixed at the index admission, and seven of those 33 have no index-admission equivalent at all. Recomputing every feature at the index admission lowered the prevalence of all 18 comorbidity and medication flags and moved the partition beyond refit noise, so a bedside version would have to be derived from index-admission data from the outset. Third, the phenotype would have to carry information a clinician does not already have. Here it adds 0.050 to the optimism-corrected concordance index of a model containing age, sex, CHA_2_DS_2_-VASc, and three routine laboratory values, but a conventional regression that also uses utilization variables discriminates slightly better without using the phenotypes at all, so the case for them rests on interpretability rather than on prediction. Fourth, and decisively, phenotype membership would have to modify treatment effect and not merely prognosis. Nothing in this study addresses that. Establishing it would require either a prospective cohort with protocolized treatment or a secondary analysis of randomized trial data in which the phenotype is derived from baseline variables and tested as an effect modifier for a specific decision, such as early rhythm control, referral for ablation, or anticoagulant choice. Until such an analysis exists, these phenotypes are a way of describing AF heterogeneity, not a basis for allocating treatment.

The modest formal cluster separation metrics, including a silhouette score of 0.1386 against 0.1185 for the single reference partition the identical pipeline produces on permuted data (the mean across permuted-null resampling replicates is 0.1149, standard deviation 0.0104; Supplementary Table S10), and an end-to-end bootstrap Adjusted Rand Index of 0.406 against 0.199 for that same permuted null, represent a substantial limitation of this work and should temper confidence in the reproducibility of individual cluster assignments. Two of the six phenotypes were recovered only marginally above that null, and their 95% intervals extended below it. These results indicate that patients likely lie along overlapping continua rather than in sharply distinct groups, and that the six-phenotype partition is one categorical summary of what is fundamentally continuous variation. What reproduces under refitting is the magnitude of the between-phenotype mortality gradient, not the assignment of an individual patient or the ordering of the middle phenotypes, so these should be understood as reproducible strata along a continuum rather than as six discrete entities. Neither bootstrap variant propagates uncertainty in model selection, because the hyperparameters and the number of clusters were held fixed throughout. The number of clusters is itself not determined by the data: the four internal validity indices do not converge on k = 6, the silhouette coefficient being maximal at k = 6, the gap statistic at k = 7, the Calinski-Harabasz index at k = 4, and the Davies-Bouldin index at k = 8, and k = 6 was chosen among several statistically near-equivalent solutions on reproducibility, per-cluster event counts, and interpretability. The phenotype labels are descriptive shorthand for standardized feature profiles rather than adjudicated clinical categories; the chronicity phenotype in particular rests on codes that are present at the index admission in only 56.1% of the patients who ever carry them. Notwithstanding these limitations, the phenotypes showed clinically meaningful differences in outcomes, including a 37.0 percentage point range in mortality and highly significant pairwise survival differences in 14 of 15 comparisons, and formed clinically coherent profiles consistent with known AF biology.^9^

Three further limitations follow from the survival models. First, age and sex were themselves inputs to the representation-learning model, so even the primary adjusted model reduces rather than eliminates the overlap between the covariates and the phenotype definitions; no adjustment set available in this design is fully independent of the exposure. Second, the proportional hazards assumption was rejected in every model, so the reported hazard ratios summarize an average relative hazard over a period in which that hazard falls several fold. Third, the reference phenotype was chosen because it has the longest observed follow-up, not because it is unbiased; its follow-up advantage is itself a function of healthcare utilization, and it is not the least censored phenotype overall.

Censoring in this cohort is informative, and the problem is structural rather than incidental. Patients not known to have died were censored at the last recorded discharge, so censoring time is itself a measure of healthcare utilization; for the 4,904 patients (35.1%) with a single admission who were censored alive, follow-up is identically the index length of stay, and because MIMIC-IV records no outpatient encounters, deaths occurring after discharge are not captured. Removing the utilization variables from the clustering model does not remove utilization from the analysis, because reverse Kaplan-Meier follow-up still ranged from 6.5 to 1,264.5 days across the baseline-only clusters. Discrimination statistics inherit the same imbalance: the patient pairs that are comparable under censoring are not a random sample of all pairs, so both the concordance index and the time-dependent areas under the curve are affected, and the time-dependent estimates additionally rely on a censoring model that assumes censoring is independent of covariates, an assumption these data contradict. They are reported as descriptive rather than as unbiased measures of discrimination. Finally, no a priori sample size or power calculation was performed. The entire eligible cohort was analyzed, so precision was determined by the number of patients meeting the eligibility criteria rather than by a target precision. The phenotype-level comparisons are well powered at this sample size, with no phenotype containing fewer than 324 deaths, but the landmark and sensitivity analyses were not powered in advance, and the smaller of them, such as the 90-day landmark analysis in which 32.6% of the cohort remained, should be read accordingly.

Several directions for future work follow from these findings. First, external validation in independent cohorts is essential if these phenotypes are to be generalized beyond the single-center MIMIC-IV population; the failure of temporal validation to meet the pre-specified distribution threshold suggests that phenotype prevalence may shift across time periods and across healthcare settings, even where the associations with outcomes appear more stable. Second, the question of incremental value is now partly answered and partly open: the phenotypes add discrimination beyond age, sex, CHA_2_DS_2_-VASc, and routine laboratory values, but not beyond a conventional model that also uses utilization variables, so the open question is whether they add anything to treatment selection rather than to prognosis. Third, prospective studies are needed to test whether phenotype-guided treatment strategies improve outcomes compared with standard care. No treatment-response analysis was performed in this study, so any therapeutic implications remain speculative and hypothesis-generating, and these phenotypes should be viewed as hypothesis-generating rather than directly actionable for clinical decision-making. Fourth, future models should incorporate time-varying covariates so that they can capture the evolution of comorbidities and AF progression. The present analysis compresses longitudinal disease trajectories into static ever-present flags, and the index-admission sensitivity analysis shows that this is not a cosmetic simplification: it inflates chronic diagnosis prevalence by a mean of 5.0 percentage points, mixes calendar era into apparent disease burden, and materially changes which patients cluster together. Finally, future phenotyping efforts may benefit from integrating additional data modalities, including electrocardiographic features, imaging measures of atrial structure and function, and biomarkers of inflammation and fibrosis.^2,22^ These additions could sharpen phenotype definitions and provide deeper mechanistic insight.

## Conclusion

This study identified six AF phenotypes using a VAE-based unsupervised learning framework. The phenotypes differed substantially in observed-death proportions, which ranged from 11.1% to 48.1% over markedly unequal observation windows, and in 30-day readmission, which ranged from 13.0% to 42.1%. They also differed in intensive care utilization, which ranged from 33.6% to 77.8%, and in treatment patterns; because intensive care admission and cumulative length of stay were themselves inputs to the clustering model, those two contrasts describe the phenotypes rather than serving as independent outcomes, and the intensive care spread falls from 44.2 to 25.1 percentage points when the phenotypes are rebuilt without utilization variables. Statistically significant differences were observed across nearly all pairwise survival comparisons. The six phenotypes, designated Phenotype 1 (Cerebrovascular-Predominant), Phenotype 2 (Hypoalbuminemic-Anemic), Phenotype 3 (Low Comorbidity Burden), Phenotype 4 (High AF Chronicity), Phenotype 5 (Warfarin-Predominant Anticoagulation), and Phenotype 6 (Renal Dysfunction), showed coherent clinical profiles consistent with known pathophysiologic patterns. Several of them largely reflect comorbidity groupings already embedded in established risk scores; others, such as the warfarin-predominant group, offer hypothesis-generating signals for further study rather than directly actionable targets. Formal cluster separation metrics were modest, and full end-to-end resampling showed that individual assignments are only moderately reproducible, indicating that phenotype boundaries are soft, even though a between-phenotype mortality gradient was present in all 100 end-to-end resamples. Adding phenotype improved discrimination for mortality beyond age, sex, CHA_2_DS_2_-VASc, and routine laboratory values, but a conventional model built from routinely available variables without any phenotype term discriminated slightly better, so the contribution of these phenotypes is interpretive stratification rather than improved prediction. Censoring heterogeneity and short follow-up in the phenotypes with the briefest observation further limit the strength of comparative survival conclusions. Within those limits, the reproducible outcome gradient and the coherent clinical profiles suggest that VAE-based phenotyping captures meaningful heterogeneity within AF. These findings support further development and prospective testing of phenotype-guided approaches to AF risk stratification, with the longer-term goal of identifying subgroups that derive differential benefit from specific anticoagulation, rhythm control, or comorbidity management strategies.

## Supplemental Material

Supplemental Material - Unsupervised Phenotyping of Atrial Fibrillation Using Variational Autoencoders in a Critical Care Cohort: The ATLAS-AF Study Supplemental Material for Unsupervised Phenotyping of Atrial Fibrillation Using Variational Autoencoders in a Critical Care Cohort: The ATLAS-AF Study by Samer G. Salman, BSA, Akhil Javvadi, MS, Zane G. Salman, Sai Madhav Yedupati, MS, Alessandra Pina, MD, Prashanthan Sanders, PhD, MBBS, Sainyam Galhotra, PhD, Ajay Tripuraneni in Clinical Medicine Insights: Cardiology

Supplemental Material - Unsupervised Phenotyping of Atrial Fibrillation Using Variational Autoencoders in a Critical Care Cohort: The ATLAS-AF Study Supplemental Material for Unsupervised Phenotyping of Atrial Fibrillation Using Variational Autoencoders in a Critical Care Cohort: The ATLAS-AF Study by Samer G. Salman, BSA, Akhil Javvadi, MS, Zane G. Salman, Sai Madhav Yedupati, MS, Alessandra Pina, MD, Prashanthan Sanders, PhD, MBBS, Sainyam Galhotra, PhD, Ajay Tripuraneni in Clinical Medicine Insights: Cardiology

## Data Availability

The data that support the findings of this study are available from the Medical Information Mart for Intensive Care IV (MIMIC-IV) database (version 3.1), available through PhysioNet (https://physionet.org/). Access to the database requires completion of the required data use agreements and credentialing process. The analytical code used in this study is available from the corresponding author upon reasonable request. No new datasets were generated beyond the processing and analysis of the MIMIC-IV database.
