# Supplementary material for: Unsupervised Phenotyping of Atrial Fibrillation Using Variational Autoencoders in a Critical Care Cohort: The ATLAS-AF Study
Source: Clin Med Insights Cardiol. 2026 Sep 11;20:11795468261490000. doi: 10.1177/11795468261490000 (PMC13569996; doi:10.1177/11795468261490000)
Supplement: Supplementary file 1 [file sj-pdf-1-cic-10.1177_11795468261490000.pdf]

Supplementary Checklist S1. STROBE and RECORD Reporting Checklist

Observational cohort study conducted using routinely collected health data

Manuscript: Unsupervised Phenotyping of Atrial Fibrillation Using Variational Autoencoders in a Critical Care Cohort

Supplied with the revised manuscript as Supplementary Checklist S1.

This completed checklist accompanies the revised manuscript and is supplied as a supplementary file in response to the Associate Editor's request that the reporting of the study adhere to the relevant EQUATOR Network guideline. The revised Methods names the guideline, provides a reference citation, and cites this file as Supplementary Checklist S1, in the subsection headed Reporting guideline.

Guideline selection. This is a retrospective observational cohort study of patients with atrial fibrillation, assembled from a single routinely collected critical care database (MIMIC-IV version 3.1) and followed for all-cause mortality. The applicable EQUATOR guideline is therefore the STROBE statement, cohort variant, and the applicable extension is RECORD, which covers studies conducted using routinely collected observational health data. Both are completed below. STROBE items 1 to 22 are reported in full, including every cohort-specific sub-item; sub-items that apply only to case-control or cross-sectional designs are not reproduced. RECORD items are prefixed RECORD, shaded, and placed immediately after the STROBE item each one extends, which is the layout used in the published RECORD checklist.

Reported? key. Yes means the item is addressed in the revised manuscript at the stated location. Partly means the item is only partly addressed, and the note states specifically what is missing. No means the item is not reported, and the note states why and what would be needed to report it. Not applicable means the item does not apply to this study design, and the note gives the reason. No status has been assigned without checking the corresponding text in the revised manuscript.

Locations. Locations are given as manuscript section and subsection headings and as table and figure numbers. Page and line numbers are deliberately not used, because pagination changes with typesetting. The Methods of the revised manuscript is organized under the following subsection headings, in this order: Study design, setting, and period; Data source; Inclusion and exclusion criteria; Sample size; Reporting guideline; Feature engineering; Missingness assessment and imputation; Variational autoencoder for representation learning; Phenotype discovery and characterization; Survival analysis and follow-up; Statistical analysis; Validation and sensitivity analyses. Where an item is addressed on the separate title page rather than in the blinded main document, for example funding, this is stated in the location column.

Item wording. STROBE and RECORD item text is reproduced from the published statements, condensed where an item is long, and rendered in American English for consistency with the manuscript.

Item	Checklist item (topic)	Reported?	Location in revised manuscript (section)
**Title and Abstract**			
**1a**	Title and abstract: indicate the study's design with a commonly used term in the title or the abstract.	Yes	Title; Abstract (Methods). The title identifies a critical care cohort, and the Abstract Methods heading names the design as a retrospective observational cohort study of routinely collected health data.
**1b**	Title and abstract: provide in the abstract an informative and balanced summary of what was done and what was found.	Yes	Abstract, structured under Objective, Methods, Results, and Conclusion. The summary is balanced by design: the Conclusion carries the negative findings, namely that temporal validation did not meet the pre-specified distribution threshold and that individual phenotype assignments are not reproducible when the model is refit end to end.
**RECORD 1.1**	RECORD extension. The type of data used should be specified in the title or abstract. Where possible, the name of the databases used should be included.	Yes	Abstract (Methods). Names MIMIC-IV version 3.1 and identifies the data as routinely collected administrative and electronic health record data rather than data collected for research.
**RECORD 1.2**	RECORD extension. If applicable, the geographic region and time frame within which the study took place should be reported in the title or abstract.	**Partly**	Abstract (Methods) gives the study period. The geographic region and the identity of the single contributing center are given in Methods (Study design, setting, and period; Data source) rather than in the title or abstract. Missing: the region is not named in the title or abstract.

Item	Checklist item (topic)	Reported?	Location in revised manuscript (section)
**RECORD 1.3**	RECORD extension. If linkage between databases was conducted for the study, this should be clearly stated in the title or abstract.	Not applicable	No linkage between databases was performed. All variables derive from a single source, MIMIC-IV version 3.1. Records were consolidated within that database to the patient level using the MIMIC-IV subject identifier, which is a within-database operation and not a data linkage.
**Introduction**			
**2**	Background and rationale: explain the scientific background and rationale for the investigation being reported.	Yes	Introduction, paragraphs 1 to 3. Covers the burden of atrial fibrillation, the limits of duration-based classification and conventional risk scores, prior cluster-analytic phenotyping studies, and the rationale for nonlinear representation learning.
**3**	Objectives: state specific objectives, including any pre-specified hypotheses.	Yes	Introduction, final paragraph; Abstract (Objective). States the objective and the pre-specified hypothesis that clustering in a variational autoencoder latent space would identify phenotypes differing in mortality, healthcare utilization, and treatment patterns.
**Methods**			
**4**	Study design: present key elements of study design early in the paper.	Yes	Methods (Study design, setting, and period), the first Methods subsection. Names the design, the unit of analysis (one row per patient), the study period, and the retrospective, secondary-use nature of the data.
**5**	Setting: describe the setting, locations, and relevant dates, including periods of recruitment, exposure, follow-up, and data collection.	Yes	Methods (Study design, setting, and period); Methods (Survival analysis and follow-up). Single academic medical center; anchor year groups 2014 to 2016, 2017 to 2019, and 2020 to 2022 define the study window. The Methods states that MIMIC-IV shifts admission dates into the future to protect confidentiality, so true calendar dates cannot be reported and the anchor year group is the finest available time resolution. Time zero for follow-up is defined as the first admission carrying an I48 code.
**6a**	Participants: give the eligibility criteria, and the sources and methods of selection of participants; describe methods of follow-up.	Yes	Methods (Inclusion and exclusion criteria); Methods (Data source); Methods (Survival analysis and follow-up); Figure 1. Inclusion and exclusion criteria are given as a separate Methods subsection in response to the editor. Follow-up runs from the first I48-coded admission to death or to the last recorded discharge, at which point a surviving patient is censored.
**6b**	Participants (cohort studies): for matched studies, give matching criteria and the number of exposed and unexposed.	Not applicable	No matching was performed and there is no exposed and unexposed contrast. Every patient meeting the eligibility criteria entered the analysis, and group membership is a phenotype label derived post hoc by unsupervised clustering rather than an assigned or observed exposure.
**RECORD 6.1**	RECORD extension. The methods of study population selection, such as the codes or algorithms used to identify subjects, should be listed in detail. If this is not possible, an explanation should be provided.	Yes	Methods (Inclusion and exclusion criteria). The six ICD-10 I48 codes used to identify atrial fibrillation are listed individually, together with the severity hierarchy used to resolve an encounter to a single AF subtype. The revision states explicitly that ICD-9 coding of atrial fibrillation (427.3x) was not used and that patients coded only in ICD-9 were excluded.
**RECORD 6.2**	RECORD extension. Any validation studies of the codes or algorithms used to select the population should be referenced. If validation was conducted for this study and not published elsewhere, detailed methods and results should be provided.	**No**	Not reported. No published validation study of the ICD-10 I48 case definition in MIMIC-IV is cited, and no internal validation of the case definition against chart review or electrocardiographic confirmation was performed. The revised Discussion states that AF and AF subtype were inferred from administrative codes rather than clinically adjudicated, and reports that only 280 of 499 patients (56.1%) ever coded as longstanding persistent or permanent AF still carry that code at the index admission. That is a disclosure of the limitation,

Item	Checklist item (topic)	Reported?	Location in revised manuscript (section)
			not a validation reference. Converting this item to Yes would require citing an external validation study of I48 coding or performing chart review, neither of which is possible in a deidentified database without clinical notes access for this purpose.
**RECORD 6.3**	RECORD extension. If the study involved linkage of databases, consider use of a flow diagram or other graphical display to demonstrate the data linkage process, including the number of individuals with linked data at each stage.	Not applicable	No database linkage was performed, so there is no linkage process to display. A selection flow diagram covering all eligibility steps is provided as Figure 1.
**7**	Variables: clearly define all outcomes, exposures, predictors, potential confounders, and effect modifiers; give diagnostic criteria if applicable.	Yes	Methods (Feature engineering); Methods (Survival analysis and follow-up); Supplementary Table S1 (feature definitions with MIMIC-IV source and coverage). The primary outcome is all-cause mortality; secondary outcomes are 30-day readmission, ICU admission, cumulative length of stay, and treatment pattern. The exposure is phenotype membership. The revision adds three definitional clarifications that the submitted version left implicit: the AF duration feature is the interval between the first and last AF-coded admission and is zero for single-admission patients, so it measures repeated coded contact rather than measured disease duration; the HAS-BLED implementation omits labile international normalized ratio and substitutes a warfarin exposure term; and both clinical risk scores were computed from patient-level ever-present flags rather than from index-encounter values.
**RECORD 7.1**	RECORD extension. A complete list of codes and algorithms used to classify exposures, outcomes, confounders, and effect modifiers should be provided. If these cannot be reported, an explanation should be provided.	**Partly**	Methods (Inclusion and exclusion criteria); Supplementary Table S1. The AF case-definition codes are listed in full, and Supplementary Table S1 gives the definition, MIMIC-IV source table, and coverage of every analysis feature. Missing: the complete ICD-9 and ICD-10 code lists behind the comorbidity flags, and the drug name matching strings behind the medication flags, are not printed in the manuscript or supplement. They are implemented in the analysis code, which the Data Availability statement makes available from the corresponding author.
**8**	Data sources and measurement: for each variable of interest, give sources of data and details of methods of assessment (measurement); describe comparability of assessment methods if there is more than one group.	Yes	Methods (Data source); Methods (Feature engineering); Supplementary Table S1. Every variable is derived from routinely collected billing and clinical data in MIMIC-IV, not from measurement performed for research. Comorbidity and medication variables come from diagnosis codes and prescription records, laboratory values from the laboratory results table, and utilization variables from admission and ICU stay records. Assessment is identical across phenotypes by construction, because phenotype labels are assigned after all variables have been extracted from a single common pipeline.
**9**	Bias: describe any efforts to address potential sources of bias.	Yes	Methods (Validation and sensitivity analyses); Methods (Survival analysis and follow-up); Results (Follow-Up, Censoring, and Landmark Analyses); Results (Validation). Five specific sources of bias are addressed with pre-planned analyses in the revision: informative censoring, tested by reverse Kaplan-Meier estimation, a censoring-hazard model, a change of reference phenotype, and landmark analyses at 30 and 90 days; circularity between clustering inputs and utilization outcomes, tested by re-clustering with the utilization features removed; compression of longitudinal information by ever-present encoding, tested by re-clustering on index-admission values only; collinearity between the CHA2DS2-VASc score and phenotype-defining features, tested by

Item	Checklist item (topic)	Reported?	Location in revised manuscript (section)
			variance inflation factors, with the primary adjustment restricted to age and sex; and violation of the proportional hazards assumption, tested by scaled Schoenfeld residuals, with period-specific hazard ratios reported.
**10**	Study size: explain how the study size was arrived at.	**Partly**	Methods (Sample size); Discussion (limitations). The derivation of the study size is explained: no sampling was performed, and the analysis includes every patient in MIMIC-IV version 3.1 meeting the eligibility criteria, giving 13,967 patients and 4,531 deaths. Missing: no a priori sample size or power calculation was performed, because the cohort size is fixed by the source database and cannot be increased. The revision states this explicitly in the Sample size subsection and carries it as a limitation in the Discussion, and reports the resulting per-phenotype analytic power (the smallest phenotype contains 1,906 patients, and the fewest deaths in any phenotype is 324).
**11**	Quantitative variables: explain how quantitative variables were handled in the analyses; if applicable, describe which groupings were chosen and why.	Yes	Methods (Feature engineering); Methods (Missingness assessment and imputation); Methods (Variational autoencoder for representation learning). Continuous laboratory variables were summarized as the median across a patient's encounters and standardized before model input; binary variables were kept on the 0 to 1 scale. No continuous variable was banded into categories for analysis. The revision states that ever-present encoding of binary variables mixes observation time with disease burden, and reports the index-admission sensitivity analysis that quantifies the effect.
**12a**	Statistical methods: describe all statistical methods, including those used to control for confounding.	Yes	Methods (Statistical analysis); Methods (Survival analysis and follow-up); Methods (Variational autoencoder for representation learning); Methods (Phenotype discovery and characterization). Covers chi-square and Kruskal-Wallis testing, Kaplan-Meier estimation with global and Bonferroni corrected pairwise log-rank tests, Cox proportional hazards models, the autoencoder architecture and training, and k-means clustering with the justification for k equal to 6. Confounding control is by regression adjustment: the primary adjusted model includes age and sex only, and the manuscript states why the CHA2DS2-VASc score was moved to a secondary model, namely that its components were themselves clustering inputs. The manuscript also states that age and sex were clustering inputs as well, so no fully independent adjustment set exists in this design.
**12b**	Statistical methods: describe any methods used to examine subgroups and interactions.	**Partly**	Methods (Statistical analysis); Methods (Survival analysis and follow-up); Results (Follow-Up, Censoring, and Landmark Analyses). All principal analyses are stratified by phenotype, and the revision adds time-stratified hazard ratios over three intervals (0 to 30 days, 30 to 365 days, and beyond 365 days) plus landmark analyses at 30 and 90 days, which together characterize the interaction between phenotype and follow-up time. Missing: no formal multiplicative interaction terms were fitted, and no subgroup analysis by age band, sex, or anticoagulation status was performed or tested.
**12c**	Statistical methods: explain how missing data were addressed.	Yes	Methods (Missingness assessment and imputation). Missingness was quantified before imputation: of 35 analysis features, 31 were complete, 5 had less than 5% missing, and 1 had 5% to 30% missing. Laboratory features with less than 30% patient-level coverage were excluded from model input rather than imputed. Remaining continuous features were imputed by k-nearest-neighbors imputation with k equal to 5, and any residual missing values by the

Item	Checklist item (topic)	Reported?	Location in revised manuscript (section)
			population median. No patient was excluded because of missing data.
**12d**	Statistical methods (cohort studies): if applicable, explain how loss to follow-up was addressed.	**Partly**	Methods (Survival analysis and follow-up); Results (Follow-Up, Censoring, and Landmark Analyses); Discussion (limitations). Censoring is characterized in unusual detail for this revision: reverse Kaplan-Meier median follow-up overall and by phenotype, percentage censored, numbers at risk, a formal test of informative censoring, and landmark analyses. What is missing is stated in the revision as a limitation rather than solved: true loss to follow-up cannot be distinguished from administrative censoring, because a surviving patient is censored at the last discharge recorded in the database and deaths occurring outside the contributing hospital are not captured. Follow-up is therefore severely unequal across phenotypes, from a reverse Kaplan-Meier median of 5.3 days in Phenotype 3 to 854.9 days in Phenotype 4, and for 4,904 patients (35.1% of the cohort) who had a single admission and were censored alive, follow-up is identical to the index length of stay. No inverse probability of censoring weighting is used for the primary analysis, because the censoring mechanism is demonstrably outcome-related.
**12e**	Statistical methods: describe any sensitivity analyses.	Yes	Methods (Validation and sensitivity analyses); Results (Validation, subsection Sensitivity Analyses); Results (Incremental Discrimination Beyond Routine Variables). Nine sensitivity analyses are pre-specified and reported: alternative cluster numbers from k equal to 4 through 8; alternative latent dimensions; re-clustering with healthcare utilization features removed; re-clustering on index-admission values only; a null pipeline run on column-permuted data; an end-to-end bootstrap that refits the autoencoder inside every resample; an alternative reference phenotype for the Cox models; administrative censoring at 365 days; and landmark analyses at 30 and 90 days.
**RECORD 12.1**	RECORD extension. Authors should describe the extent to which the investigators had access to the database population used to create the study population.	Yes	Methods (Data source); Figure 1. The investigators held credentialed PhysioNet access to the complete MIMIC-IV version 3.1 release. The entire database population of 364,627 patients was available and screened, and Figure 1 reports the count remaining after each eligibility step, so the denominator from which the study population was drawn is fully visible.
**RECORD 12.2**	RECORD extension. Authors should provide information on the data cleaning methods used in the study.	Yes	Methods (Feature engineering); Methods (Missingness assessment and imputation). Cleaning steps are specified: consolidation of multiple encounters to one row per patient, the ever-present rule for binary comorbidity and medication flags, the median rule for repeated laboratory values, resolution of multiple AF subtype codes by a stated severity hierarchy, exclusion of laboratory features with less than 30% patient-level coverage, imputation of the remainder, and standardization of continuous features before model input. Fields that were empty for the entire cohort were excluded from the analysis and are not reported in any table.
**RECORD 12.3**	RECORD extension. State whether the study included person-level, institutional-level, or other data linkage across two or more databases. The methods of linkage and methods of linkage quality evaluation should be provided.	Not applicable	No linkage across two or more databases was undertaken, so no linkage methods or linkage quality evaluation apply. Methods (Data source) states that all variables derive from MIMIC-IV version 3.1 alone, and Methods (Feature engineering) describes the within-database consolidation of encounters to the patient level.
**Results**			
**13a**	Participants: report the numbers of individuals at each stage of the study,	Yes	Results (Study Cohort); Figure 1. 364,627 patients screened, 20,952 with at

Item	Checklist item (topic)	Reported?	Location in revised manuscript (section)
	for example the numbers potentially eligible, examined for eligibility, confirmed eligible, included in the study, completing follow-up, and analyzed.		least one I48 diagnosis, 19,433 after restriction to ICD-10 coding, and 13,967 in the final analytic cohort. All 13,967 patients were analyzed; none were excluded after entry, and none were lost between inclusion and analysis.
**13b**	Participants: give reasons for non-participation at each stage.	Yes	Figure 1; Methods (Inclusion and exclusion criteria). Each exclusion is shown with its own reason and count: no AF diagnosis (n equal to 343,675), atrial fibrillation coded only in ICD-9 (n equal to 1,519), and anchor year group before 2015 (n equal to 5,466). Non-participation in the conventional sense does not arise, because the cohort is assembled retrospectively from routinely collected data with no recruitment, consent, refusal, or withdrawal.
**13c**	Participants: consider use of a flow diagram.	Yes	Figure 1, a selection flow diagram with counts retained and counts excluded at every stage.
**RECORD 13.1**	RECORD extension. Describe in detail the selection of the persons included in the study, including filtering based on data quality, data availability, and linkage. The selection of included persons can be described in the text or by means of the study flow diagram, or both.	Yes	Methods (Inclusion and exclusion criteria); Results (Study Cohort); Figure 1. Selection is described in both text and flow diagram. Filtering on data availability is stated explicitly and at the correct level: the coding-era filter (ICD-10 availability, implemented as the anchor year group restriction) removed patients, whereas the data quality filter on laboratory coverage removed features rather than patients. No patient was excluded for missing data. No linkage-based filtering applies.
**14a**	Descriptive data: give the characteristics of study participants, for example demographic, clinical, and social characteristics, and information on exposures and potential confounders.	Yes	Results (Baseline Characteristics); Table 1; Supplementary Table S4 (standardized z-score profiles by phenotype); Figure 3. Table 1 reports demographic, comorbidity, medication, laboratory, risk score, and utilization characteristics for the whole cohort and for each of the six phenotypes.
**14b**	Descriptive data: indicate the number of participants with missing data for each variable of interest.	**Partly**	Methods (Missingness assessment and imputation); Supplementary Table S1 (coverage column, which gives patient-level coverage for every analysis feature). The Methods gives the distribution of missingness across the 35 analysis features, and Supplementary Table S1 gives per-feature coverage, from which the number missing follows directly. Missing: Table 1 does not report per-variable missing counts within each phenotype, so missingness is reported cohort-wide rather than by exposure group.
**14c**	Descriptive data (cohort studies): summarize follow-up time, for example the average and total amount.	Yes	Results (Follow-Up, Censoring, and Landmark Analyses); Supplementary Table S8. Reverse Kaplan-Meier median follow-up is reported with a confidence interval overall and for each phenotype, alongside percentage censored and numbers at risk at fixed time points. The revision emphasizes rather than hides the fact that follow-up is severely unequal across phenotypes, from a median of 5.3 days in Phenotype 3 to 854.9 days in Phenotype 4, and that this inequality is itself utilization-driven.
**15**	Outcome data (cohort studies): report the numbers of outcome events or summary measures over time.	Yes	Results (Primary Outcome); Results (Secondary Outcomes); Table 1; Figure 4; Supplementary Table S8. 4,531 deaths occurred among 13,967 patients. Deaths, observed mortality proportions, 30-day readmission, ICU admission, and cumulative length of stay are reported for each phenotype, with Kaplan-Meier curves and numbers at risk. Because follow-up is severely unequal across phenotypes, the revision labels the crude percentages as observed-death proportions accrued over unequal observation windows rather than as comparable risks, and reports Kaplan-Meier estimates and identification bounds alongside them.

Item	Checklist item (topic)	Reported?	Location in revised manuscript (section)
**16a**	Main results: give unadjusted estimates and, if applicable, confounder-adjusted estimates and their precision, for example 95% confidence intervals; make clear which confounders were adjusted for and why they were included.	Yes	Results (Primary Outcome); Table 2; Figure 5. Unadjusted hazard ratios, the primary model adjusted for age and sex, and a secondary model additionally adjusted for the CHA2DS2-VASc score are all reported with 95% confidence intervals and model concordance. The rationale for each adjustment set is stated: the primary set is restricted to age and sex because the CHA2DS2-VASc components were themselves clustering inputs, and the manuscript states that age and sex were clustering inputs too, so no adjustment set in this design is fully independent of the exposure.
**16b**	Main results: report category boundaries when continuous variables were categorized.	**Partly**	Methods (Phenotype discovery and characterization); Discussion. No continuous variable was banded into categories for analysis, so the item has no literal application. It applies in spirit to the exposure, because the six-phenotype partition is itself a categorical summary of continuous latent variation. Missing: the boundaries cannot be reported as cut-points, because they are k-means decision boundaries in a 10-dimensional learned latent space and have no closed form in the original variables. The revision states this directly and describes the phenotypes as reproducible strata along a continuum rather than as discrete entities.
**16c**	Main results: if relevant, consider translating estimates of relative risk into absolute risk for a meaningful time period.	**Partly**	Results (Primary Outcome); Results (Hazard Rate vs. Cumulative Mortality Paradox); Supplementary Table S8. Absolute measures are reported: observed mortality proportions per phenotype, Kaplan-Meier survival at 1, 3, and 5 years, and, for the revision, identification bounds on 1-year mortality. Missing: the hazard ratios are not converted into absolute risk differences at a fixed horizon, and the revision explains why. Informative censoring leaves 1-year mortality poorly identified in at least one phenotype, where the Kaplan-Meier estimate of 51.2% sits inside bounds running to 94.4%, so a single absolute risk difference would convey false precision.
**17**	Other analyses: report other analyses done, for example analyses of subgroups and interactions, and sensitivity analyses.	Yes	Results (Validation), with subsections Temporal Split Validation, Bootstrap Stability, and Sensitivity Analyses; Results (Incremental Discrimination Beyond Routine Variables); Results (Follow-Up, Censoring, and Landmark Analyses). Reported analyses include temporal split validation, bootstrap stability at both the clustering step and end to end with the autoencoder refit, a permuted-data null, cluster number and latent dimension sensitivity, re-clustering without utilization features, re-clustering on index-admission values, time-stratified and landmark survival models, and a comparison of discrimination against conventional models built from routinely available variables.
**Discussion**			
**18**	Key results: summarize the key results with reference to the study objectives.	Yes	Discussion, opening paragraph; Conclusion. The summary is tied back to the stated objective and reports the outcome-level result (a wide and reproducible mortality gradient) separately from the individual-level result (phenotype assignment is not reproducible under a full refit).
**19**	Limitations: discuss the limitations of the study, taking into account sources of potential bias or imprecision; discuss both the direction and magnitude of any potential bias.	Yes	Discussion, limitations paragraphs. Direction and magnitude are given numerically rather than in general terms. Instability of individual assignment: end-to-end adjusted Rand index 0.406 against an empirical permuted-data null of 0.199, not the analytic chance value of 0.091 used in the submitted version. Circularity of utilization comparisons: the ICU admission spread across

Item	Checklist item (topic)	Reported?	Location in revised manuscript (section)
			phenotypes falls from 44.2 to 25.1 percentage points when the phenotypes are rebuilt without utilization features, and the length of stay gap named by a reviewer falls from 4.3-fold to 2.0-fold. Ever-present encoding: recomputing every feature from the index admission alone yields a partition that agrees with the published solution at an adjusted Rand index of 0.148 at seed 42 and 0.159 averaged over five initializations, where merely refitting the unchanged feature set against that same published solution gives 0.432. Read refit against refit at matched seeds, the index-admission and cumulative encodings of the same 28 features agree with each other at 0.291, against a structurally matched within-arm control of 0.533, so the encoding moves the partition further than refit noise does. Informative censoring, violation of the proportional hazards assumption, the absence of an a priori power calculation, and the single-center critically ill source population are each stated as limitations.
**RECORD 19.1**	RECORD extension. Discuss the implications of using data that were not created or collected to answer the specific research question. Include a discussion of misclassification bias, unmeasured confounding, missing data, and changing eligibility over time, as they pertain to the study being reported.	Yes	Discussion, limitations and generalizability paragraphs. All four required topics are covered. Misclassification: AF and AF subtype are inferred from administrative codes and were not clinically adjudicated, and only 56.1% of patients ever coded as longstanding persistent or permanent AF still carry that code at the index admission. Unmeasured confounding: no echocardiographic, electrocardiographic, symptom, or ambulatory medication data are available, and the treatment variables are subject to confounding by indication. Missing data: the coverage-based exclusion of features and the imputation strategy are stated, along with the fact that a value absent from a billing record is not evidence that the condition is absent. Changing eligibility over time: record length collapses across the study window, from a mean of 3.47 encounters per patient in the earliest anchor year group to 1.59 in the latest, so ever-present flags accumulate differentially by era and cumulative exposure measures are not comparable across eras.
**20**	Interpretation: give a cautious overall interpretation of the results, considering the objectives, limitations, multiplicity of analyses, results from similar studies, and other relevant evidence.	Yes	Discussion. Interpretation is explicitly hypothesis-generating. The revision states that several phenotypes recapitulate comorbidity groupings already captured by existing tools, and concedes that a conventional model built from routinely available variables reaches higher discrimination than the phenotype model without using phenotypes at all. Multiplicity is handled by Bonferroni correction for the 15 pairwise survival comparisons, which is stated. Comparison with prior cluster-analytic AF phenotyping studies is provided.
**21**	Generalizability: discuss the generalizability, that is the external validity, of the study results.	Yes	Discussion, dedicated generalizability paragraph added in the revision. States that MIMIC-IV is a single-center critical care database, that the cohort is acutely ill and hospital-based with an overall mortality above 30%, that the phenotypes should not be extrapolated to ambulatory, community, or registry-based AF populations, and that temporal validation within the same institution did not meet the pre-specified distribution threshold, which bounds the expectation of transportability across settings.
**Other Information**			
**22**	Funding: give the source of funding and the role of the funders for the present study and, if applicable, for the original study on which the present article is based.	Yes	Title page, Statements and Declarations (Funding): the authors received no financial support for the research, authorship, or publication of this article. There is therefore no funder role to report, and no original funded study underlies the present analysis. The statement appears on the separate title page

Item	Checklist item (topic)	Reported?	Location in revised manuscript (section)
			rather than in the main document because the main document is blinded for review in accordance with journal policy. The same page carries the ethics statement (institutional review board approval was not required for use of the publicly available MIMIC-IV database), the conflict of interest declaration, and the data availability statement.
**RECORD 22.1**	RECORD extension. Authors should provide information on how to access any supplemental information such as the study protocol, raw data, or programming code.	**Partly**	Title page, Statements and Declarations (Data Availability). Access to the raw data is fully specified: MIMIC-IV version 3.1 is distributed through PhysioNet and requires completion of the data use agreement and credentialing process, and the analysis code is available from the corresponding author. Missing: no study protocol or statistical analysis plan was prepared or registered in advance, and the analysis code is not deposited in a public repository with a persistent identifier. Converting this item to Yes would require depositing the code and stating that no protocol was registered.

Summary: 33 Yes, 9 Partly, 1 No, 4 Not applicable (of 47 items: 34 STROBE cohort sub-items and 13 RECORD extension items).

The authors have not claimed full compliance on every item. The items below are the ones a reader should scrutinize, with the reason each falls short stated in the table above and summarized here.

Item not reported (No):

RECORD 6.2. Validation studies of the codes or algorithms used to select the population. Missing: no published validation of the ICD-10 I48 case definition in this database is cited, and no internal validation against chart review was performed.

Chart-level adjudication of the case definition is not possible in the deidentified release, so the revision states the limitation and quantifies its likely effect rather than claiming a validated case definition.

Items only partly reported (Partly), with what is missing:

RECORD 1.2. Geographic region and time frame in the title or abstract. Missing: the study period is in the abstract, but the geographic region is named in Methods rather than in the title or abstract.

RECORD 7.1. Complete list of codes and algorithms. Missing: the AF case-definition codes and every feature definition are given, but the full comorbidity code lists and drug name matching strings are not printed; they are in the analysis code.

10. Study size. Missing: no a priori sample size or power calculation was performed. The cohort size is fixed by the source database, and the revision states this in the Sample size subsection and carries it as a limitation.

12b. Methods used to examine subgroups and interactions. Missing: no formal interaction terms were fitted and no subgroup analysis by age band, sex, or anticoagulation status was performed. Phenotype-stratified and time-stratified analyses are reported.

12d. How loss to follow-up was addressed. Missing: true loss to follow-up cannot be separated from administrative censoring, because a surviving patient is censored at the last discharge in the database and out-of-hospital deaths are not captured. Censoring is characterized in detail instead.

14b. Number of participants with missing data for each variable. Missing: per-feature coverage is given in Supplementary Table S1 and the overall distribution in Methods, but Table 1 does not report missing counts separately within each phenotype.

16b. Category boundaries when continuous variables were categorized. Missing: no continuous variable was banded, and the phenotype boundaries are k-means decision boundaries in a 10-dimensional learned latent space, so they have no reportable cut-points in the original variables.

16c. Translating relative risk into absolute risk. Missing: absolute measures are reported, but hazard ratios are not converted into absolute risk differences at a fixed horizon, because informative censoring leaves 1-year mortality poorly identified in at least one phenotype.

RECORD 22.1. How to access supplemental information. Missing: raw data access and code availability are stated, but no protocol or analysis plan was registered and the code is not deposited in a public repository with a persistent identifier.

Items marked Not applicable: STROBE 6b (no matching was performed), RECORD 1.3, RECORD 6.3, and RECORD 12.3 (no linkage between databases was undertaken, so the linkage items do not apply).

References for the guidelines cited in the Methods:

von Elm E, Altman DG, Egger M, Pocock SJ, Gotzsche PC, Vandenbroucke JP; STROBE Initiative. The Strengthening the Reporting of Observational Studies in Epidemiology (STROBE) statement: guidelines for reporting observational studies. Lancet. 2007;370(9596):1453-1457. doi:10.1016/S0140-6736(07)61602-X

Benchimol EI, Smeeth L, Guttmann A, et al; RECORD Working Committee. The REporting of studies Conducted using Observational Routinely-collected health Data (RECORD) statement. PLoS Med. 2015;12(10):e1001885. doi:10.1371/journal.pmed.1001885
