# Supplementary material for: Unsupervised Phenotyping of Atrial Fibrillation Using Variational Autoencoders in a Critical Care Cohort: The ATLAS-AF Study
Source: Clin Med Insights Cardiol. 2026 Sep 11;20:11795468261490000. doi: 10.1177/11795468261490000 (PMC13569996; doi:10.1177/11795468261490000)
Supplement: Supplementary file 2 [file sj-pdf-2-cic-10.1177_11795468261490000.pdf]

SUPPLEMENTARY MATERIAL

Note on cluster labels. The six phenotypes of the primary analysis are named Phenotype 1 (Cerebrovascular-Predominant), Phenotype 2 (Hypoalbuminemic-Anemic), Phenotype 3 (Low Comorbidity Burden), Phenotype 4 (High AF Chronicity), Phenotype 5 (Warfarin-Predominant Anticoagulation) and Phenotype 6 (Renal Dysfunction). Three further six-cluster solutions are reported as sensitivity analyses. Because cluster identifiers are not stable when the model is refit, the clusters of those solutions are lettered A to F by descending size within each solution and are never given a phenotype name. To keep the three lettered solutions distinct, every printed label carries a solution prefix: BO1-A to BO1-F for the baseline-only solution from which the three healthcare utilization variables were removed (32 variables; Supplementary Figure S11, Supplementary Table S18), BO2-A to BO2-F for the stricter baseline-only solution from which the two encounter-derived atrial fibrillation variables were also removed (30 variables; same figure and table), and IA-A to IA-F for the index-admission solution (28 variables; Supplementary Figure S12, Supplementary Tables S19 and S20). A letter alone is never used in text or in a table.

Supplementary Figures

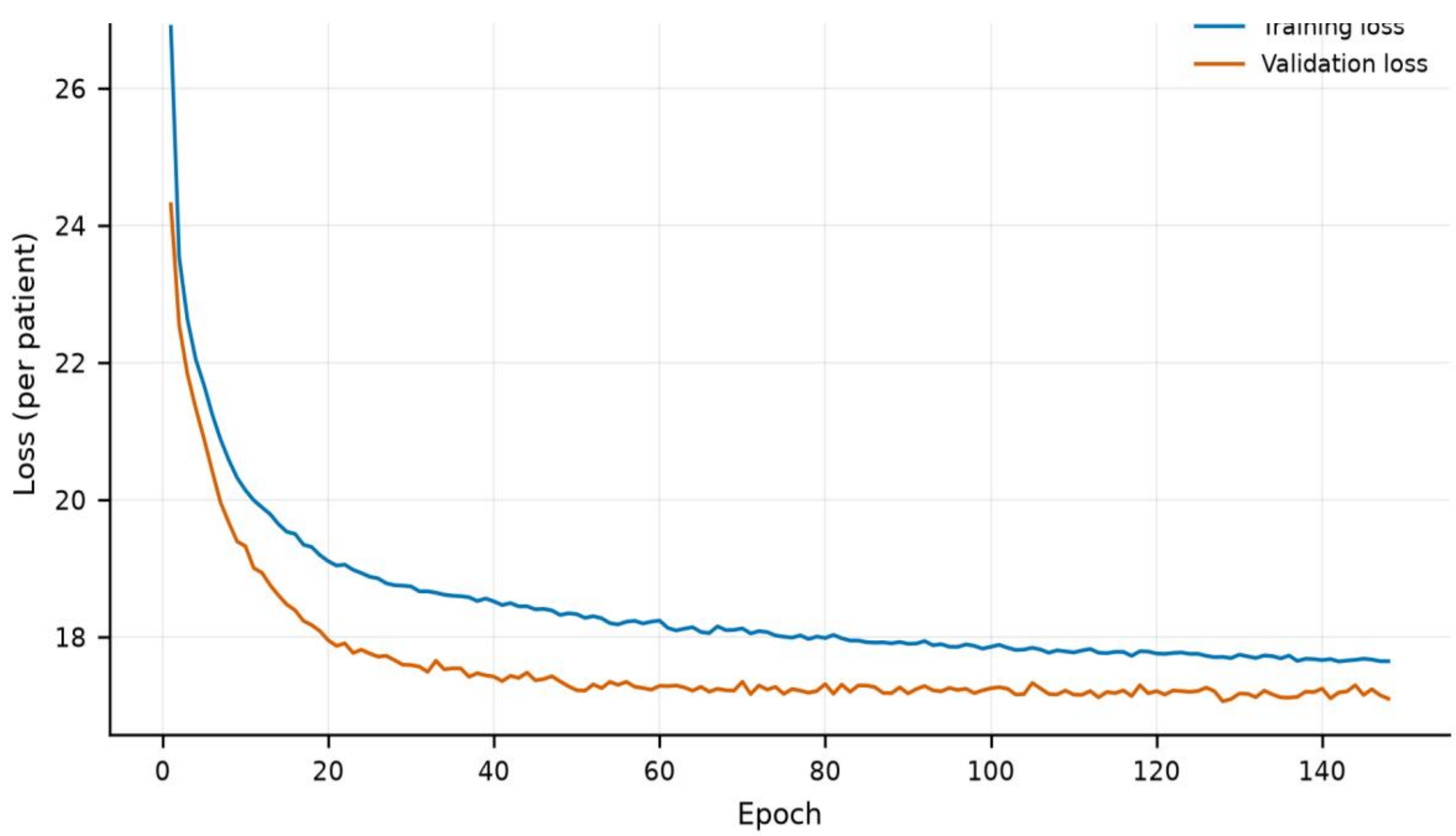

Supplementary Figure S1. VAE training and validation loss curves. Early stopping occurred at epoch 148.

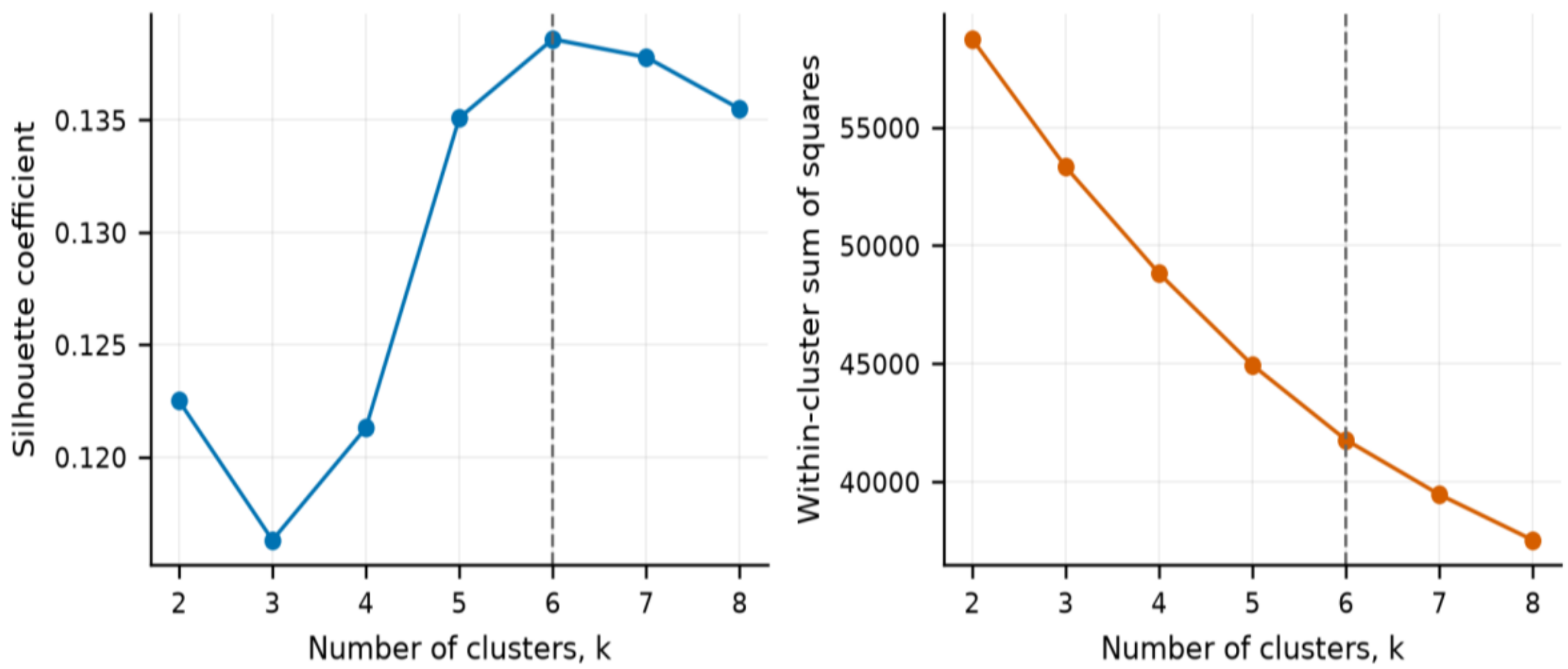

Supplementary Figure S2. Cluster number selection. (A) Silhouette coefficient and (B) within-cluster sum of squares (inertia) as a function of the number of clusters, k. The silhouette coefficient was highest at k = 6 (0.1386). The dashed vertical line in each panel marks the selected solution, k = 6.

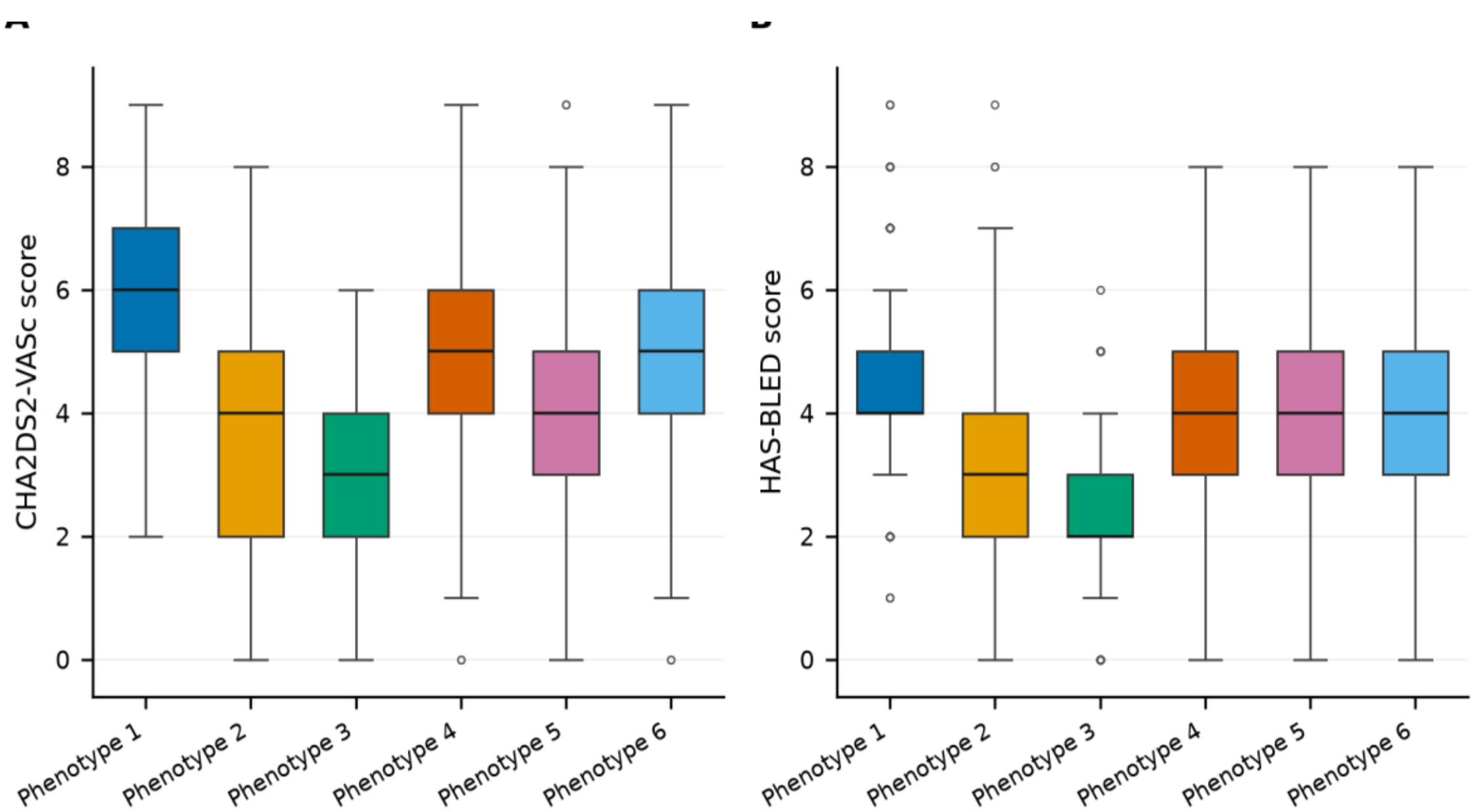

Supplementary Figure S3. Distribution of clinical risk scores by phenotype. (A) CHA2DS2-VASc score. (B) HAS-BLED score. Boxes span the interquartile range, the horizontal line is the median, whiskers

extend to 1.5 times the interquartile range and points beyond the whiskers are plotted individually. Both scores were computed from patient-level ever-recorded flags across the whole record rather than from index-admission values, and both were inputs to the clustering. Phenotypes: Phenotype 1, Cerebrovascular-Predominant; Phenotype 2, Hypoalbuminemic-Anemic; Phenotype 3, Low Comorbidity Burden; Phenotype 4, High AF Chronicity; Phenotype 5, Warfarin-Predominant Anticoagulation; Phenotype 6, Renal Dysfunction.

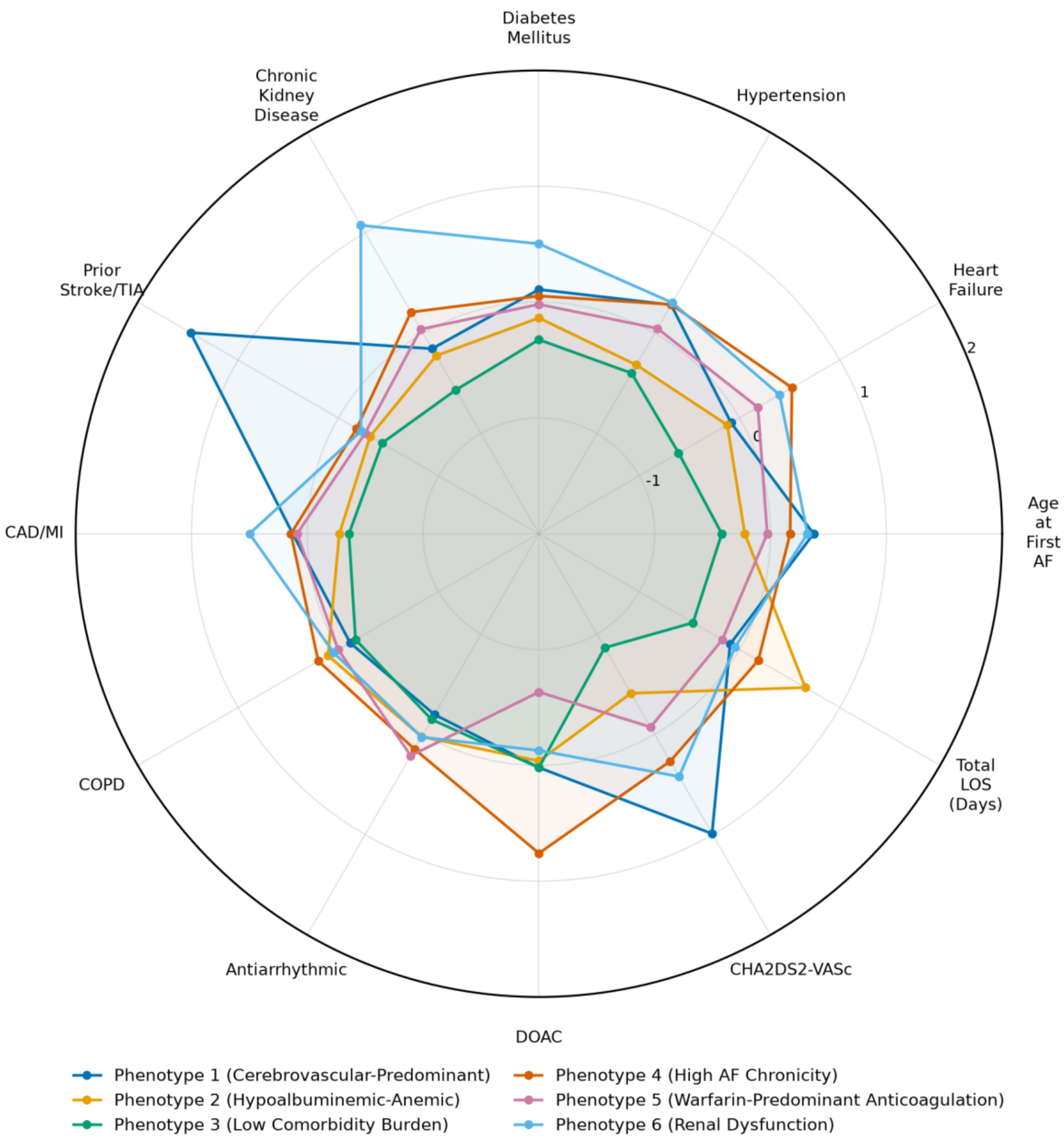

Supplementary Figure S4. Radar plot of key clinical features by phenotype. Values are within-phenotype means expressed as z-scores against the cohort mean, on a common radial scale from -2 to 2. Features shown are a curated subset spanning comorbidity burden (heart failure, hypertension, diabetes mellitus, chronic kidney disease, coronary artery disease or myocardial infarction, chronic obstructive pulmonary disease, prior stroke or transient ischemic attack), treatment (antiarrhythmic drug, direct oral anticoagulant), age at first AF, CHA2DS2-VASc score and healthcare utilization (total length of stay).

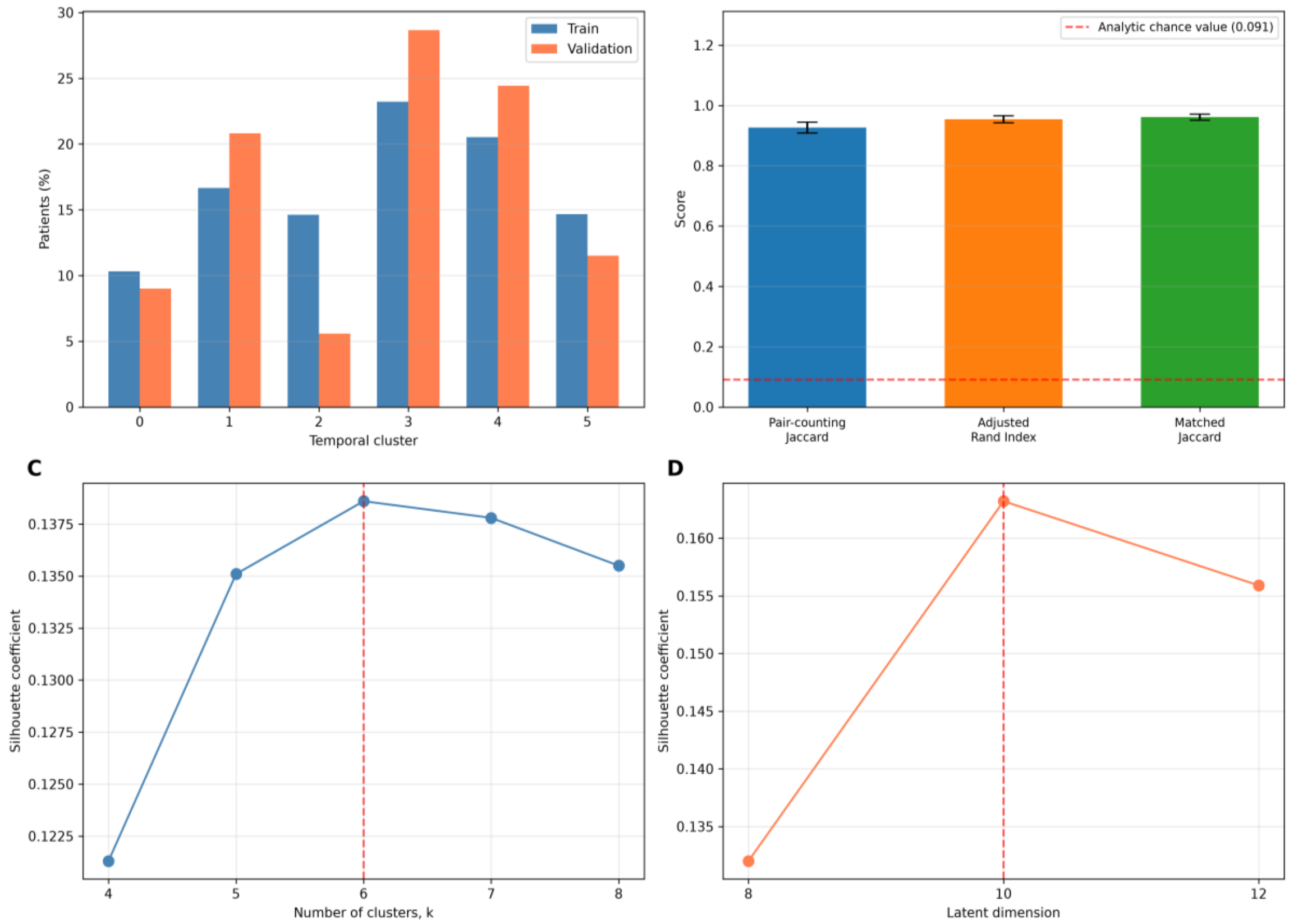

Supplementary Figure S5. Validation panel. (A) Replication of the phenotype distribution between the temporal training window (anchor year group 2014 to 2016, n = 4,072) and the temporal validation window (anchor year groups 2017 to 2019 and 2020 to 2022, n = 9,895), shown as the percentage of patients in each temporal cluster; temporal clusters are numbered as in Supplementary Table S5, and the maximum absolute difference between the two distributions was 9.0 percentage points, which did not meet the prespecified threshold of 5 percentage points. (B) Bootstrap stability of the six-phenotype

partition over 100 iterations of 80 percent subsampling with the trained variational autoencoder held fixed and K-means refit, showing the mean pair-counting Jaccard similarity, the mean adjusted Rand index and the mean per-cluster matched Jaccard index; error bars are one standard deviation and the dashed line is the analytic chance value of 1/(2k-1) = 0.091 for the pair-counting Jaccard. That analytic constant understates the agreement this pipeline produces on data with no joint structure, so an empirical permuted-data null is also reported (Supplementary Figure S8, Supplementary Table S10). (C) Silhouette coefficient as a function of the number of clusters from k = 4 to k = 8; the dashed vertical line marks the selected solution, k = 6. (D) Silhouette coefficient as a function of the latent dimension; the dashed vertical line marks the selected latent dimension of 10.

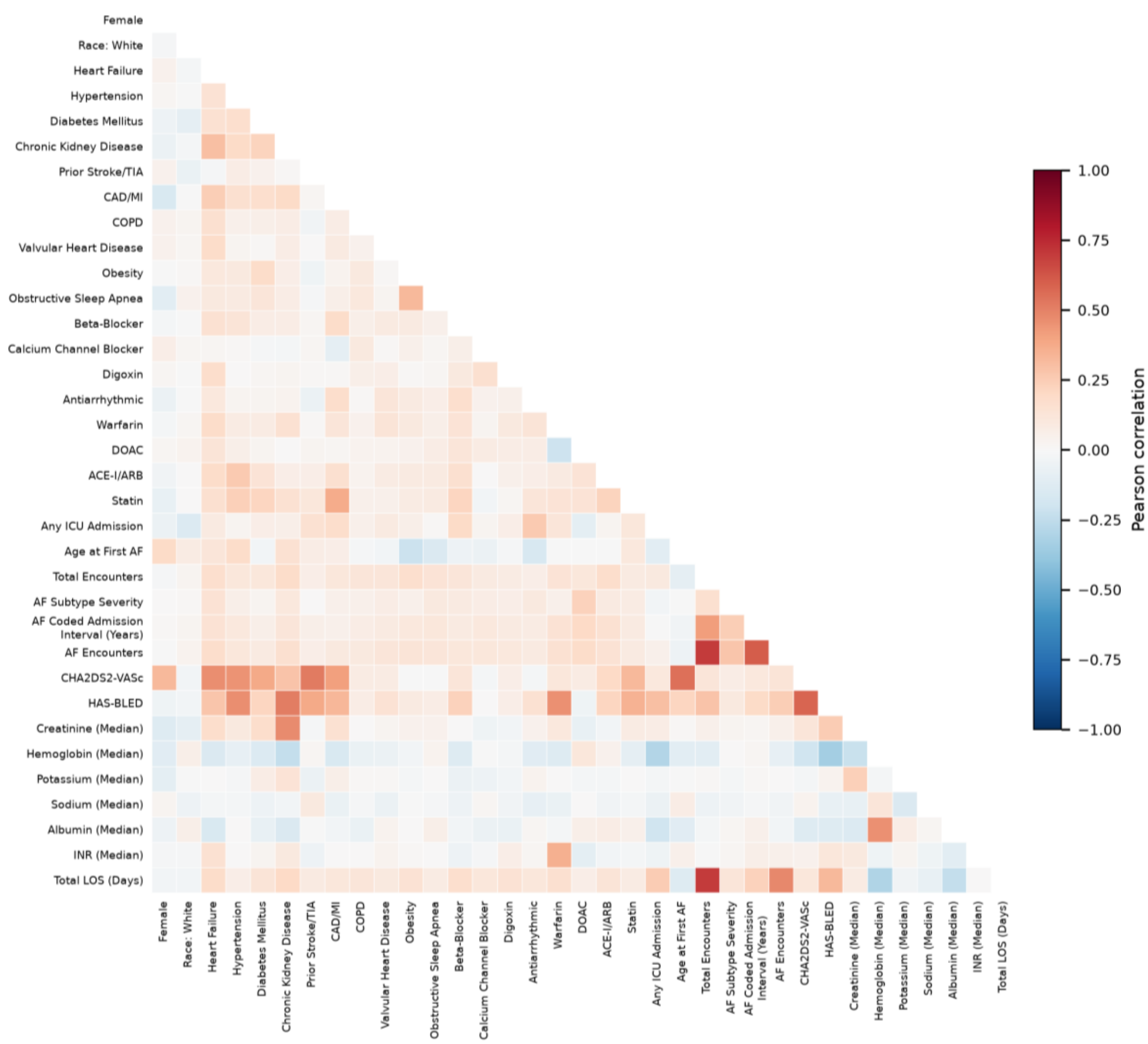

Supplementary Figure S6. Feature correlation matrix. Pearson correlation coefficients between the 35 clustering features, shown for the lower triangle only. Variables are named as in Supplementary Table S1; the AF coded admission interval is the interval in years between the first and last AF-coded admission and is 0.0 for patients with a single admission.

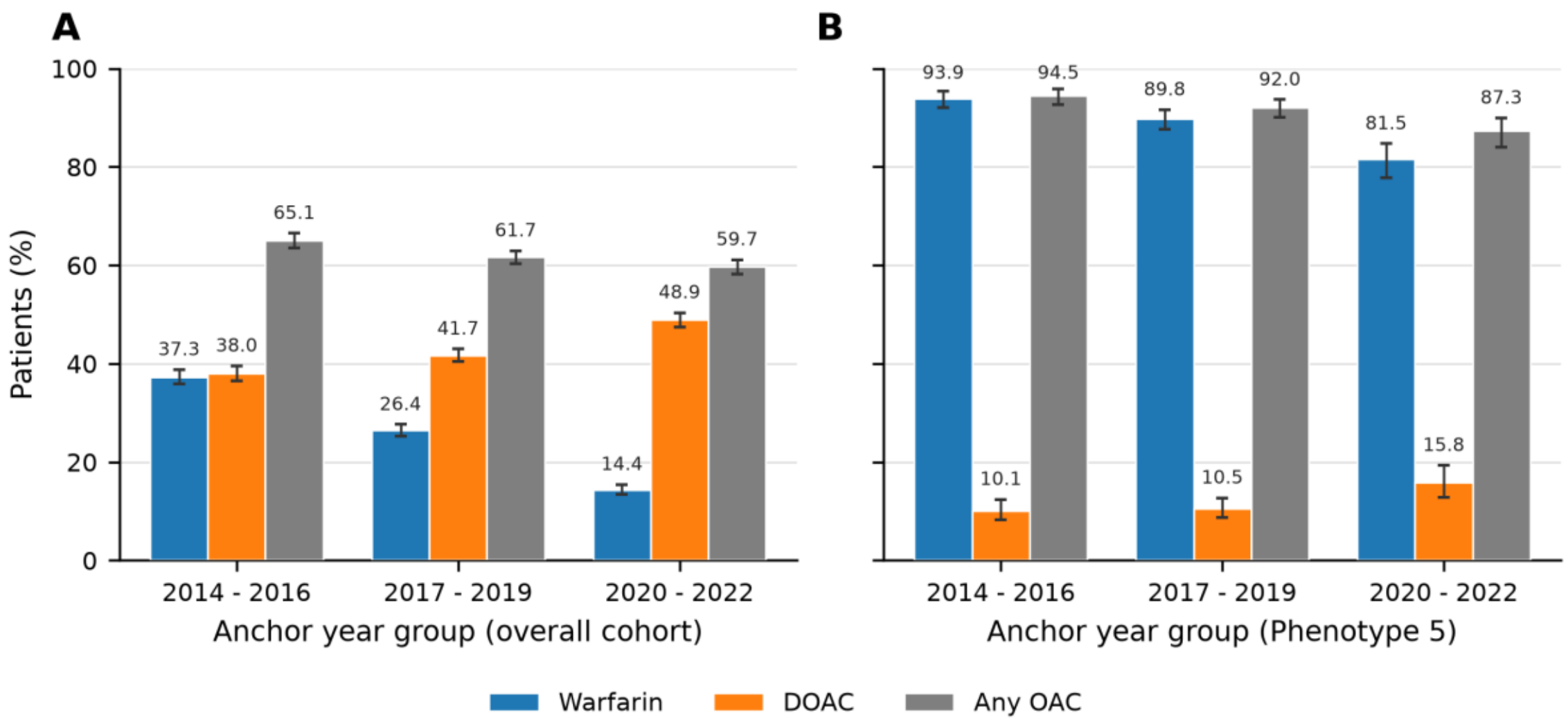

Supplementary Figure S7. Oral anticoagulant exposure by MIMIC-IV anchor year group. Bars show the percentage of patients with an ever-recorded prescription for warfarin (blue), a direct oral anticoagulant (DOAC, orange), or either agent (any OAC, gray) within each anchor year group; error bars are Wilson 95% confidence intervals and numerals above each bar give the point estimate. (A) Whole study cohort (n = 13,967). (B) Phenotype 5 (Warfarin-Predominant Anticoagulation, n = 2,178). Warfarin and DOAC flags are patient-level ever-exposed indicators over the entire MIMIC-IV record and are not mutually exclusive, so the warfarin and DOAC bars may sum to more than the any-OAC bar; mutually exclusive counts (warfarin only, DOAC only, both, neither) are reported in the accompanying supplementary table. Because these are cumulative ever-exposed flags rather than index-encounter prescriptions, absolute levels exceed encounter-level prescribing rates, and patients anchored in earlier windows have longer records and more opportunity to accumulate any exposure; the era-to-era direction, not the absolute level, is the finding, and the same direction is present in the single-encounter sensitivity analysis reported in the

supplementary table. P values are two-sided Cochran-Armitage tests for trend across the three ordered anchor year groups.

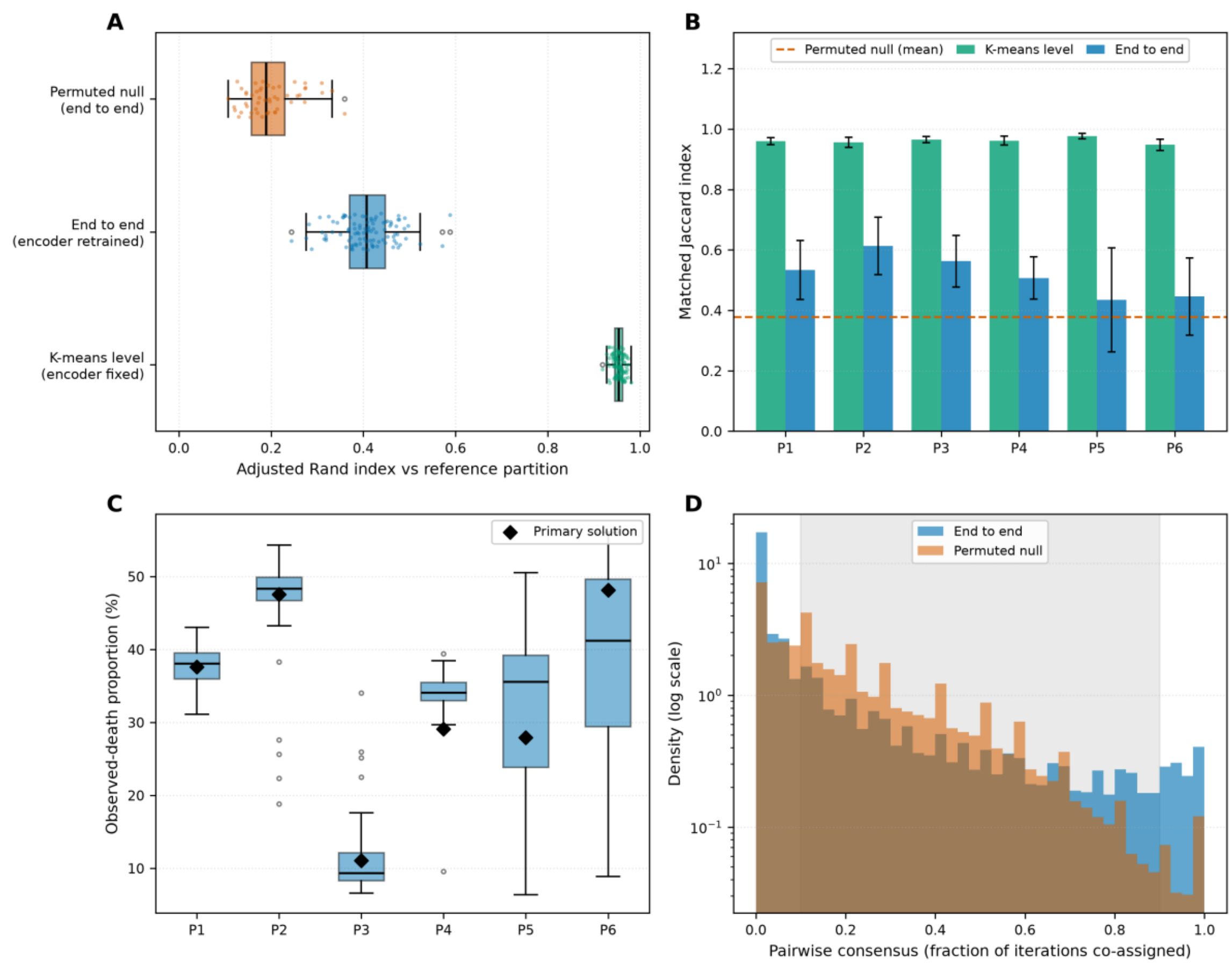

Supplementary Figure S8. Stability of the six-phenotype solution under three bootstrap schemes. (A) Distribution of the adjusted Rand index between each bootstrap partition and the reference partition, for the k-means-level bootstrap (trained encoder held fixed, clustering refit on an 80% subsample, 100 iterations), the end-to-end bootstrap (variational autoencoder retrained from scratch on each 80% subsample before reclustering, 100 iterations), and a permuted null in which every feature column was independently shuffled before the identical end-to-end pipeline was rerun (50 iterations). Boxes show the median and interquartile range, whiskers the 1.5 interquartile range, and points the individual iterations. (B) Mean matched Jaccard index for each phenotype after optimal Hungarian matching of bootstrap clusters to the primary solution; error bars are standard deviations across iterations and the dashed line is the mean matched Jaccard of the permuted null. P1 to P6 denote Phenotype 1 (Cerebrovascular-

Predominant), Phenotype 2 (Hypoalbuminemic-Anemic), Phenotype 3 (Low Comorbidity Burden), Phenotype 4 (High AF Chronicity), Phenotype 5 (Warfarin-Predominant Anticoagulation), and Phenotype 6 (Renal Dysfunction). (C) Bootstrap distribution of the observed-death proportion within each phenotype across the end-to-end iterations; black diamonds mark the value in the primary solution. These are observed-death proportions accrued over markedly unequal observation windows, not comparable risks. (D) Distribution of pairwise consensus, the fraction of iterations in which two patients were assigned to the same cluster, estimated from 5,000,000 randomly sampled patient pairs, for the end-to-end bootstrap and the permuted null. The shaded band marks the ambiguous region, consensus between 0.1 and 0.9, whose mass is the Proportion of Ambiguous Clustering. The vertical axis is on a logarithmic scale.

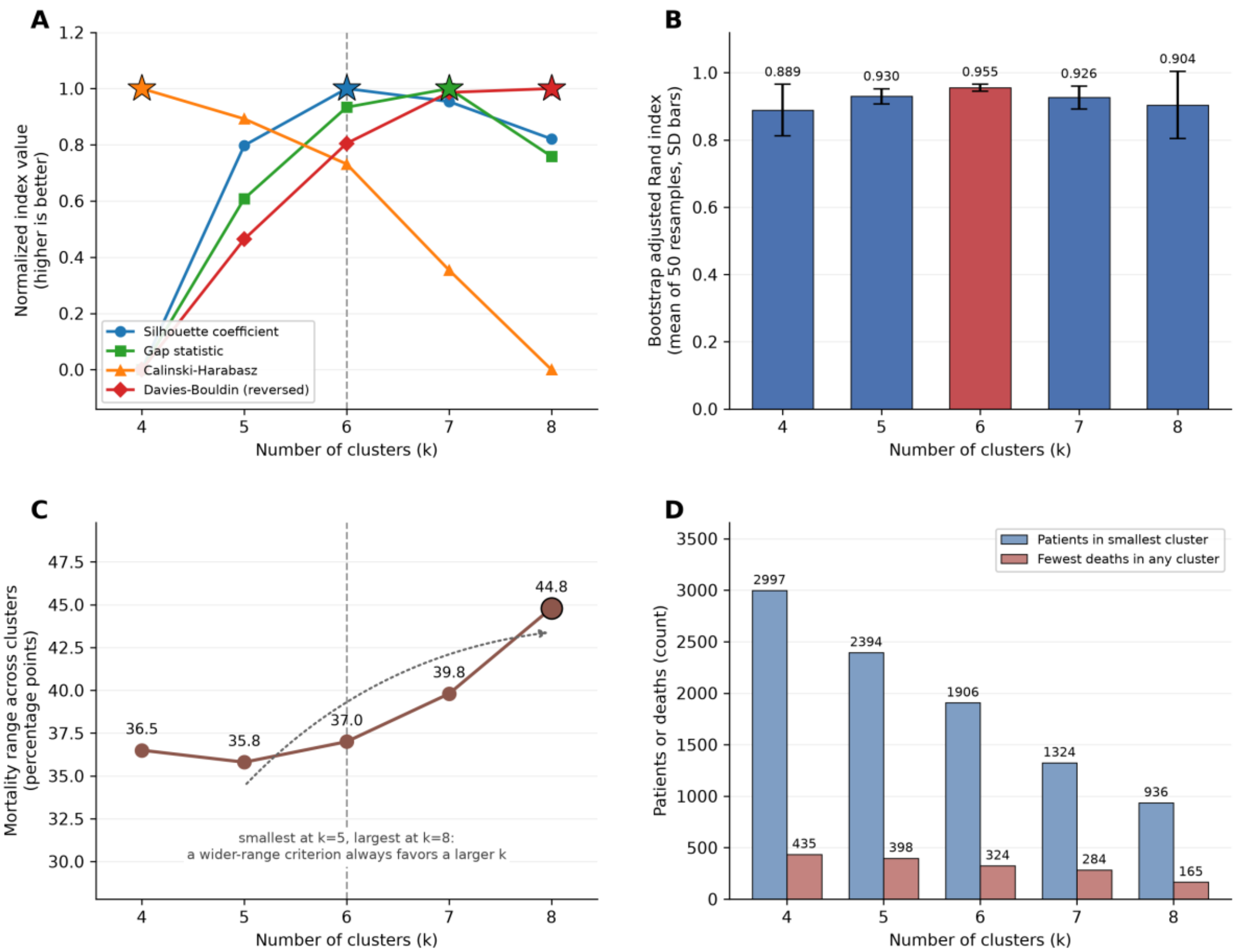

Supplementary Figure S9. Comparison of cluster-number solutions from k=4 to k=8 in the primary variational autoencoder latent space. (A) Four internal validity indices, each min-max normalized across the five solutions so that they can be shown on one axis; raw values are given in Supplementary Table S9.

Higher values are better for the silhouette coefficient, the gap statistic, and the Calinski-Harabasz index, and lower values are better for the Davies-Bouldin index, which is plotted with its sign reversed before normalization so that upward is better for all four curves. Stars mark each index's preferred k. The indices do not converge on a common k, and the absolute spread in the silhouette coefficient across this range is 0.017. (B) Bootstrap stability by k, shown as the mean adjusted Rand index over 50 iterations of 80 percent subsampling with k-means refitting and nearest-centroid assignment of the full cohort; error bars are one standard deviation. Every solution in this range is highly reproducible. (C) Mortality range between the highest and lowest cluster at each k. The range is smallest at k=5 and rises thereafter to its maximum at k=8, so a criterion based on mortality spread always favors a larger k and cannot be used to select k=6. (D) Per-cluster statistical power at each k, shown as the number of patients in the smallest cluster and the smallest number of deaths observed in any cluster of that solution; the two quantities need not refer to the same cluster. Both fall as k rises, from 2,997 patients and 435 deaths at k=4 to 936 patients and 165 deaths at k=8.

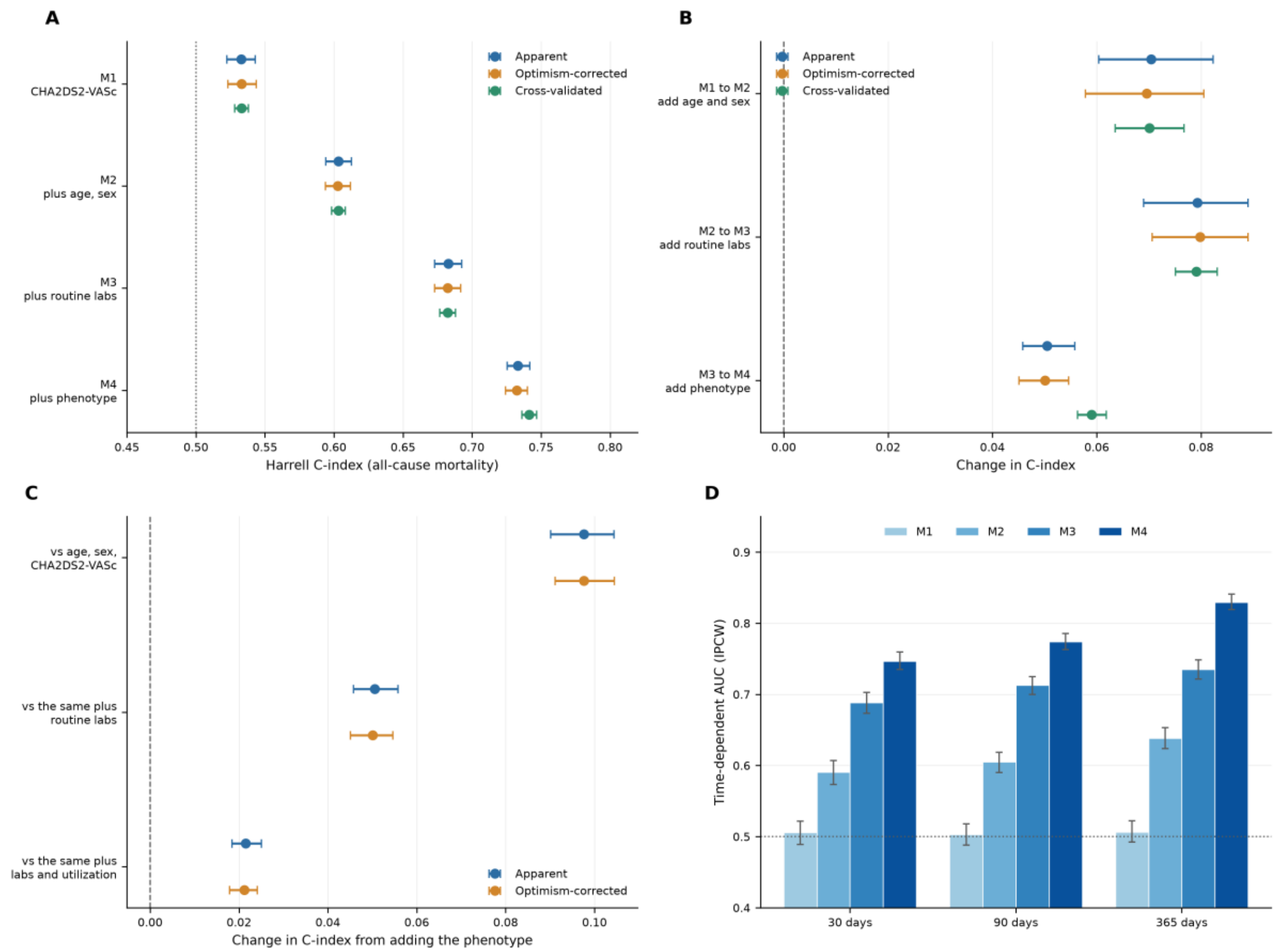

Supplementary Figure S10. Incremental discrimination for all-cause mortality beyond routinely available variables. (A) Harrell C-index for four nested Cox models: M1, CHA2DS2-VASc alone; M2, age, sex and CHA2DS2-VASc; M3, M2 plus three routine laboratory values (creatinine, hemoglobin, albumin); M4, M3 plus the six-level phenotype. Three estimates are shown for each model: apparent (full-cohort fit evaluated on the full cohort), optimism-corrected (Harrell bootstrap, 500 resamples), and cross-validated (3 repeats of stratified 5-fold cross-validation in which the standardizer, the variational autoencoder and the k-means solution were all re-derived inside each training fold and held-out patients were assigned by nearest centroid in that fold's latent space; the plotted value is the mean of the 15 per-fold validation C-indices). Error bars are 95% bootstrap percentile intervals for the apparent and optimism-corrected estimates and a 95% interval across folds for the cross-validated estimate. (B) Change in C-index at each nested step. The dashed line marks no change. (C) Change in C-index attributable to the phenotype against three different comparators, showing how the increment shrinks as routinely available information

is added to the comparator. (D) Cumulative/dynamic time-dependent AUC at 30, 90 and 365 days, estimated with inverse probability of censoring weights. Because censoring in this cohort is strongly informative and follow-up is severely unequal across phenotypes, the time-dependent estimates in panel D depend on a censoring model that is known to be misspecified and should be read as descriptive rather than as unbiased discrimination.

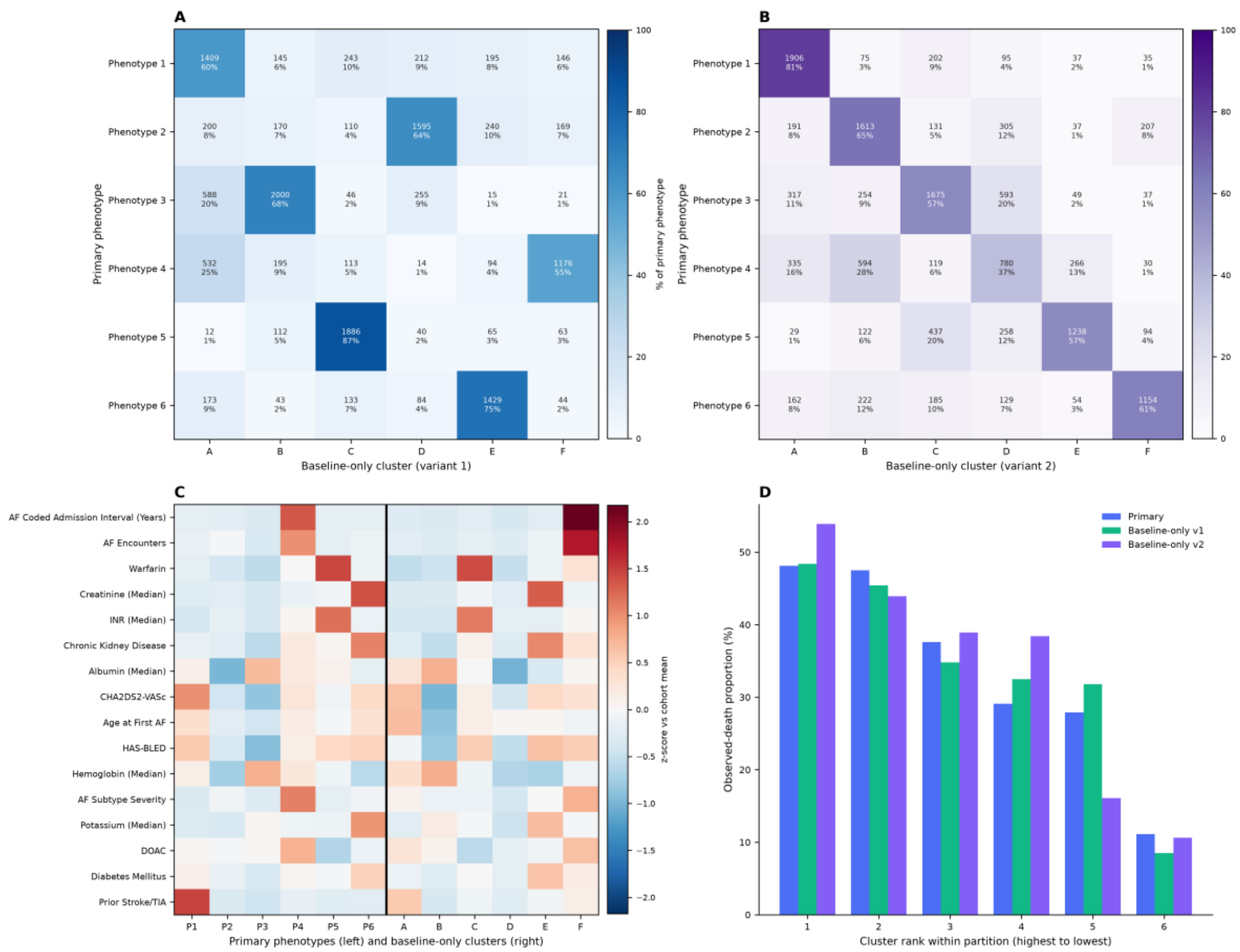

Supplementary Figure S11. Re-clustering using baseline clinical variables only. (A) Cross-tabulation of the six published phenotypes (rows) against the six clusters obtained when total length of stay, number of encounters and any ICU admission are removed from the feature set and the variational autoencoder is retrained from scratch (variant 1, columns). Cells give the patient count and, as a shaded percentage, the share of each published phenotype assigned to that baseline-only cluster. (B) The same cross-tabulation for the stricter variant 2, which additionally removes the number of atrial fibrillation coded encounters and the interval between the first and last atrial fibrillation coded admission. (C) Standardized feature

profiles for the six published phenotypes (left of the divider) and the six variant 1 baseline-only clusters (right), expressed as z-scores against the cohort mean for the 16 most discriminating clinical variables. (D) Observed-death proportions by cluster for the three partitions, with clusters ordered from highest to lowest within each partition; these are observed-death proportions over unequal follow-up, not comparable risks. Baseline-only clusters are lettered A to F because cluster identifiers are not stable across model refits and these clusters are not the published phenotypes. In this figure the letters A to F on the horizontal axis of panel A and in the right-hand block of panel C denote BO1-A to BO1-F, and the letters on the horizontal axis of panel B denote BO2-A to BO2-F, as defined in the note on cluster labels at the start of this supplement.

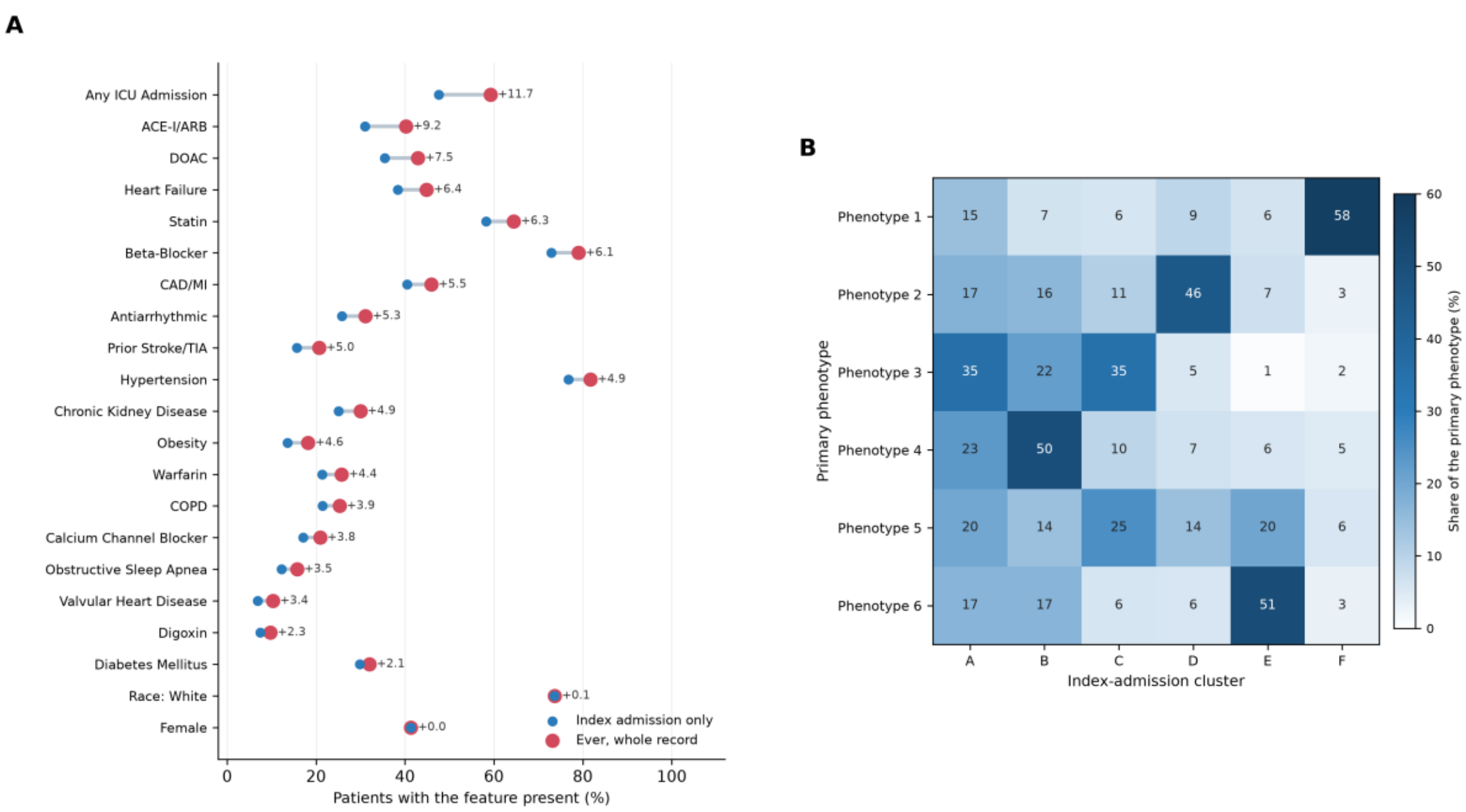

Supplementary Figure S12. Sensitivity of the six-phenotype solution to index-admission feature encoding. (A) Prevalence of each binary clustering feature under the primary cumulative encoding, in which a feature is present if it appears anywhere in the patient's MIMIC-IV record (red), and under index-admission encoding, in which it must be recorded at the first atrial fibrillation coded admission (blue). Each pair is joined by a line and annotated with the absolute difference in percentage points. Across the 18 comorbidity and medication flags the cumulative encoding raised prevalence by a mean of 5.0 percentage points; chronic kidney disease rose from 25.1% to 30.0%. (B) Cross-classification of the

published six phenotypes against the six clusters obtained when the variational autoencoder is retrained from scratch on index-admission features only (28 features, identical architecture, hyperparameters and k). Cells give the percentage of each primary phenotype assigned to each index-admission cluster; rows sum to 100 before rounding, and the displayed whole numbers may sum to 99 or 101. Index clusters are labeled A to F in descending size and were named from their own z-score profiles; the published phenotype names were not reused. Adjusted Rand index 0.148. This should be read against the agreement expected when the model is refit at all, not against 1.0: refitting the autoencoder on the unchanged feature set with nothing but a fresh random initialization already gives a mean adjusted Rand index of 0.432 against the published solution, and an end-to-end bootstrap of the unchanged feature set gives 0.406. In this figure the letters A to F denote IA-A to IA-F, as defined in the note on cluster labels at the start of this supplement.

Supplementary Tables

Supplementary Table S1. Feature Definitions (35 Clustering Features)

Domain	Type	Feature	Description	MIMIC-IV Source	Coverage
**Demographics**	Continuous	Age at First AF	Age at first AF encounter (years)	patients.csv.gz	100%
**Demographics**	Binary	Female	Female sex	patients.csv.gz	100%
**Demographics**	Binary	Race: White	White race/ethnicity	admissions.csv.gz	100%
**Demographic**	Continuous	Total	Total hospital	admissions.csv.	100%

**s**		Encounters	encounters	gz	
**AF Characteristics**	Continuous	AF Subtype Severity	AF subtype severity (1-6 hierarchy)	diagnoses_icd.csv.gz	100%
**AF Characteristics**	Continuous	AF Coded Admission Interval (Years)	Interval in years between the first and last AF-coded admission; 0.0 for patients with a single admission	admissions.csv.gz	100%
**AF Characteristics**	Continuous	AF Encounters	Number of encounters with AF diagnosis	diagnoses_icd.csv.gz	100%
**Comorbidities**	Binary	Heart Failure	Heart failure (ever)	diagnoses_icd.csv.gz	100%
**Comorbidities**	Binary	Hypertension	Hypertension (ever)	diagnoses_icd.csv.gz	100%
**Comorbidities**	Binary	Diabetes Mellitus	Diabetes mellitus (ever)	diagnoses_icd.csv.gz	100%
**Comorbiditie**	Binary	Chronic	Chronic	diagnoses_icd.c	100%

**s**		Kidney Disease	kidney disease (ever)	sv.gz	
**Comorbiditie s**	Binary	Prior Stroke/TIA	Prior stroke/TIA (ever)	diagnoses_icd.c sv.gz	100%
**Comorbiditie s**	Binary	CAD/MI	Coronary artery disease/MI (ever)	diagnoses_icd.c sv.gz	100%
**Comorbiditie s**	Binary	COPD	COPD/asthma (ever)	diagnoses_icd.c sv.gz	100%
**Comorbiditie s**	Binary	Valvular Heart Disease	Valvular heart disease (ever)	diagnoses_icd.c sv.gz	100%
**Comorbiditie s**	Binary	Obesity	Obesity (ever)	diagnoses_icd.c sv.gz	100%
**Comorbiditie s**	Binary	Obstructive Sleep Apnea	Obstructive sleep apnea (ever)	diagnoses_icd.c sv.gz	100%
**Clinical Scores**	Continuous	CHA2DS2-VASc	CHA2DS2-VASc stroke risk score	Computed	100%
**Clinical Scores**	Continuous	HAS-BLED	HAS-BLED bleeding risk	Computed	100%

			score		
**Medications**	Binary	Beta-Blocker	Beta-blocker (ever prescribed)	prescriptions.csv.gz	100%
**Medications**	Binary	Calcium Channel Blocker	Calcium channel blocker (ever)	prescriptions.csv.gz	100%
**Medications**	Binary	Digoxin	Digoxin (ever prescribed)	prescriptions.csv.gz	100%
**Medications**	Binary	Antiarrhythmic	Antiarrhythmic (ever prescribed)	prescriptions.csv.gz	100%
**Medications**	Binary	Warfarin	Warfarin (ever prescribed)	prescriptions.csv.gz	100%
**Medications**	Binary	DOAC	DOAC (ever prescribed)	prescriptions.csv.gz	100%
**Medications**	Binary	ACE-I/ARB	ACE inhibitor/ARB (ever)	prescriptions.csv.gz	100%
**Medications**	Binary	Statin	Statin (ever prescribed)	prescriptions.csv.gz	100%

**Labs**	Continuous	Creatinine (Median)	Serum creatinine, median across encounters	labevents.csv.gz	99.6%
**Labs**	Continuous	Hemoglobin (Median)	Hemoglobin, median across encounters	labevents.csv.gz	99.5%
**Labs**	Continuous	Potassium (Median)	Serum potassium, median across encounters	labevents.csv.gz	99.5%
**Labs**	Continuous	Sodium (Median)	Serum sodium, median across encounters	labevents.csv.gz	99.5%
**Labs**	Continuous	Albumin (Median)	Serum albumin, median across encounters	labevents.csv.gz	79.6%
**Labs**	Continuous	INR (Median)	INR, median across encounters	labevents.csv.gz	96.4%
**Utilization**	Binary	Any ICU	Any ICU	icustays.csv.gz	100%

		Admission	admission (ever)		
**Utilization**	Continuous	Total LOS (Days)	Cumulative length of stay (days)	admissions.csv.gz	100%

Supplementary Table S2. VAE Hyperparameter Grid Search (18 configurations). Best: latent dimension 10, beta 0.5, learning rate 0.0005 (validation reconstruction loss 14.1157)

Latent Dim	Beta	LR	Val Recon Loss	Epochs
**8**	0.5	0.001	14.5903	75
**8**	0.5	0.0005	14.7000	78
**8**	1.0	0.001	16.6487	76
**8**	1.0	0.0005	16.6590	131
**8**	2.0	0.001	19.8461	69
**8**	2.0	0.0005	19.8081	115
**10**	0.5	0.001	14.3615	74
**10**	0.5	0.0005	14.1157	148
**10**	1.0	0.001	16.4051	94
**10**	1.0	0.0005	16.6703	115
**10**	2.0	0.001	19.6669	85
**10**	2.0	0.0005	19.6721	118
**12**	0.5	0.001	14.4886	82

**12**	0.5	0.0005	14.5449	155
**12**	1.0	0.001	16.7967	81
**12**	1.0	0.0005	17.2353	121
**12**	2.0	0.001	19.7469	95
**12**	2.0	0.0005	20.1161	75

Supplementary Table S3. Cluster Selection Metrics (k=2 through k=8)

k	Silhouette	Inertia
**2**	0.1225	58739
**3**	0.1163	53328
**4**	0.1213	48820
**5**	0.1351	44938
**6**	0.1386	41772
**7**	0.1378	39457
**8**	0.1355	37530

Supplementary Table S4. Z-Score Profiles (35 Clustering Features by 6 Phenotypes)

Feature	Phenotype 1 (Cerebrovascular-Predominant)	Phenotype 2 (Hypoalbuminemic-Anemic)	Phenotype 3 (Low Comorbidity Burden)	Phenotype 4 (High AF Chronicity)	Phenotype 5 (Warfarin-Predominant	Phenotype 6 (Renal Dysfunction)

					**Anticoagulation)**	
**Age at First AF**	+0.372	-0.222	-0.421	+0.170	-0.028	+0.319
**Female**	+0.246	+0.148	-0.210	+0.086	-0.081	-0.178
**Race: White**	-0.154	-0.097	+0.128	+0.132	+0.049	-0.083
**Total Encounters**	-0.079	+0.200	-0.337	+0.591	-0.218	-0.055
**AF Subtype Severity**	-0.309	-0.081	-0.288	+1.098	-0.191	-0.078
**AF Coded Admission Interval (Years)**	-0.200	-0.253	-0.321	+1.343	-0.190	-0.212
**AF Encounters**	-0.166	-0.045	-0.330	+1.000	-0.205	-0.111
**Heart Failure**	-0.084	-0.122	-0.606	+0.523	+0.184	+0.400
**Hypertension**	+0.283	-0.317	-0.399	+0.286	+0.047	+0.304
**Diabetes Mellitus**	+0.109	-0.137	-0.324	+0.053	-0.019	+0.503
**Chronic**	-0.155	-0.225	-0.566	+0.209	+0.040	+1.075

**Kidney Disease**						
**Prior Stroke/TIA**	+1.474	-0.318	-0.435	-0.183	-0.267	-0.227
**CAD/MI**	+0.126	-0.277	-0.360	+0.141	+0.088	+0.499
**COPD**	-0.117	+0.103	-0.172	+0.200	+0.001	+0.049
**Valvular Heart Disease**	-0.035	-0.094	-0.167	+0.214	+0.177	-0.018
**Obesity**	-0.151	-0.004	-0.084	+0.199	+0.121	-0.040
**Obstructive Sleep Apnea**	-0.115	-0.129	-0.042	+0.332	+0.011	-0.009
**CHA2DS2-VASc**	+0.991	-0.409	-0.864	+0.268	-0.072	+0.421
**HAS-BLED**	+0.571	-0.333	-0.928	+0.147	+0.439	+0.488
**Beta-Blocker**	+0.040	+0.084	-0.357	+0.230	+0.135	-0.021
**Calcium Channel Blocker**	-0.017	+0.213	-0.115	+0.213	-0.062	-0.246
**Digoxin**	-0.044	+0.133	-0.209	+0.222	+0.094	-0.153
**Antiarrhythmic**	-0.197	+0.025	-0.148	+0.148	+0.214	+0.027

**Warfarin**	-0.217	-0.363	-0.544	+0.016	+1.456	-0.105
**DOAC**	+0.019	-0.038	+0.020	+0.759	-0.633	-0.128
**ACE-I/ARB**	+0.168	-0.231	-0.189	+0.402	+0.022	-0.089
**Statin**	+0.266	-0.341	-0.353	+0.231	+0.123	+0.261
**Creatinine (Median)**	-0.259	-0.241	-0.343	-0.144	-0.059	+1.386
**Hemoglobin (Median)**	+0.135	-0.737	+0.779	+0.255	-0.091	-0.582
**Potassium (Median)**	-0.302	-0.339	+0.058	-0.101	-0.103	+0.955
**Sodium (Median)**	+0.398	-0.058	+0.048	+0.054	-0.127	-0.405
**Albumin (Median)**	+0.104	-0.992	+0.684	+0.212	+0.086	-0.219
**INR (Median)**	-0.363	-0.204	-0.372	+0.041	+1.193	-0.124
**Any ICU Admission**	+0.338	+0.377	-0.522	-0.295	+0.117	+0.088
**Total LOS (Days)**	-0.093	+0.657	-0.464	+0.188	-0.171	-0.043

The 35 features profiled here are the same 35 features defined in Supplementary Table S1 and are the complete set supplied to the variational autoencoder. Seven further variables were computed during feature engineering but were excluded from clustering and therefore appear in neither table: three

electrocardiogram-derived variables, which are available for only a minority of patients and produce a data-availability split that dominates the latent space, and four index-encounter vital sign summaries, which had no recorded values in the derived dataset because the MIMIC-IV chart events table was not used. An earlier version of this table additionally listed the three electrocardiogram variables, which is why it was captioned as 38 features; they were never clustering inputs, and they have been removed so that Supplementary Table S1 and Supplementary Table S4 describe an identical feature set. AF Coded Admission Interval (Years), listed here and in Supplementary Table S1 under the term used throughout the main text, is the interval in years between a patient's first and last AF-coded admission and is 0.0 for patients with a single admission; it measures the span of coded contact with the health system rather than the duration of the arrhythmia. Z-scores are computed within each phenotype relative to the whole-cohort mean and standard deviation of the same feature.

Supplementary Table S5. Temporal Validation: Train (2014 - 2016) vs Validation (2017 - 2019, 2020 - 2022). Cluster matching via Hungarian algorithm on z-score cosine similarity.

Temporal Cluster	Matched Phenotype	Cosine Sim	Train %	Val %	Dist Diff	Train Mort	Val Mort	Mort Diff
**0**	Phenotype 5 (Warfarin-Predominant Anticoagul	0.821	10.3%	9.0%	1.3pp	40.5%	40.1%	0.3pp

	ation)							
**1**	Phenotype 2 (Hypoalbuminemic-Anemic)	0.936	16.7%	20.8%	4.1pp	47.4%	44.7%	2.7pp
**2**	Phenotype 4 (High AF Chronicity)	0.901	14.6%	5.6%	9.0pp	37.8%	29.0%	8.8pp
**3**	Phenotype 1 (Cerebrovascular-Predominant)	0.839	23.2%	28.7%	5.5pp	36.6%	31.3%	5.3pp
**4**	Phenotype 3 (Low Comorbidity Burden)	0.955	20.5%	24.4%	3.9pp	9.6%	9.6%	0.0pp
**5**	Phenotype 6 (Renal Dysfunction)	0.892	14.7%	11.5%	3.2pp	51.6%	45.8%	5.8pp

Supplementary Table S6. Treatment Patterns by Phenotype (chi-square $p < 0.001$ for all comparisons)

Phenotype	N	Rate Control %	Rhythm Control %	Warfarin (ever exposed) %	DOAC (ever exposed) %	Any OAC %	No Anticoag %
**Phenotype 1 (Cerebrovascular-Predominant)**	2350	63.4	22.0	16.3	43.9	54.1	45.9
**Phenotype 2 (Hypoalbuminemic-Anemic)**	2484	56.7	32.3	9.9	41.1	46.9	53.1
**Phenotype 3 (Low Comorbidity Burden)**	2925	49.4	24.3	1.9	43.9	45.2	54.8
**Phenotype 4 (High AF**	2124	56.2	37.9	26.5	80.5	90.1	9.9

**Chronicity)**							
**Phenotype 5 (Warfarin-Predominant Anticoagulation)**	2178	49.3	41.0	89.4	11.6	91.9	8.1
**Phenotype 6 (Renal Dysfunction)**	1906	52.3	32.4	21.1	36.6	51.9	48.1

Warfarin (ever exposed) % and DOAC (ever exposed) % are patient-level ever-exposed flags: a patient counts toward a column if that agent was ever prescribed anywhere in the record, not only during the index admission. The two columns are therefore not mutually exclusive, a patient ever exposed to both agents is counted in both, and they do not sum to the Any OAC column; the excess is the both-agents fraction. Across the whole cohort 25.8% of warfarin-flagged patients also carry a DOAC flag. Ever exposure to both agents reflects switching or overlap across a record rather than concurrent dual anticoagulation. The same data recoded into four mutually exclusive and exhaustive categories, warfarin only, DOAC only, both warfarin and a DOAC, and no oral anticoagulant, are shown in Figure 6 of the main text and, by anchor year group, in Supplementary Table S21. For example, the 16.3% of Phenotype 1 (Cerebrovascular-Predominant) recorded here as ever exposed to warfarin appears in Figure 6 as 10.2% exposed to warfarin only and 6.0% exposed to both agents. Percentages are rounded to one decimal place independently of one another, so the exclusive categories need not add to exactly 100% and need not add to the ever-exposed value shown here.

Supplementary Table S7. Pairwise Log-Rank Comparisons (Bonferroni threshold: p < 0.0033)

Comparison	Statistic	p-value	Significant
**Phenotype 1 (Cerebrovascular-Predominant) vs Phenotype 2 (Hypoalbuminemic-Anemic)**	16.02	< 0.001	Yes
**Phenotype 1 (Cerebrovascular-Predominant) vs Phenotype 3 (Low Comorbidity Burden)**	81.61	< 0.001	Yes
**Phenotype 1 (Cerebrovascular-Predominant) vs Phenotype 4 (High AF Chronicity)**	939.68	< 0.001	Yes
**Phenotype 1 (Cerebrovascular-Predominant) vs**	22.51	< 0.001	Yes

**Phenotype 5 (Warfarin-Predominant Anticoagulation)**			
**Phenotype 1 (Cerebrovascular-Predominant) vs Phenotype 6 (Renal Dysfunction)**	20.39	< 0.001	Yes
**Phenotype 2 (Hypoalbuminemic-Anemic) vs Phenotype 3 (Low Comorbidity Burden)**	147.55	< 0.001	Yes
**Phenotype 2 (Hypoalbuminemic-Anemic) vs Phenotype 4 (High AF Chronicity)**	1307.56	< 0.001	Yes
**Phenotype 2 (Hypoalbuminemic-Anemic) vs Phenotype 5 (Warfarin-**	72.30	< 0.001	Yes

**Predominant Anticoagulation)**			
**Phenotype 2 (Hypoalbuminemic-Anemic) vs Phenotype 6 (Renal Dysfunction)**	0.14	0.71	No
**Phenotype 3 (Low Comorbidity Burden) vs Phenotype 4 (High AF Chronicity)**	306.85	< 0.001	Yes
**Phenotype 3 (Low Comorbidity Burden) vs Phenotype 5 (Warfarin-Predominant Anticoagulation)**	22.87	< 0.001	Yes
**Phenotype 3 (Low Comorbidity Burden) vs Phenotype 6 (Renal Dysfunction)**	162.48	< 0.001	Yes
**Phenotype 4 (High AF Chronicity) vs Phenotype 5**	603.34	< 0.001	Yes

**(Warfarin-Predominant Anticoagulation)**			
**Phenotype 4 (High AF Chronicity) vs Phenotype 6 (Renal Dysfunction)**	1272.96	< 0.001	Yes
**Phenotype 5 (Warfarin-Predominant Anticoagulation) vs Phenotype 6 (Renal Dysfunction)**	77.71	< 0.001	Yes

Supplementary Table S8. Follow-Up Time, Censoring, and Kaplan-Meier Survival Probabilities by Phenotype

Phenotype	Patients	Observed Deaths, n (%)	Median Survival (Days)	Observed Median Follow-Up	Reverse Kaplan-Meier Median Follow-Up, Days (95%	Censored (%)	At Risk at 1 Year, n (%)	1-Year Survival	3-Year Survival	5-Year Survival

				**(Days)**	**CI)**					
**Phenotype 1 (Cerebrovascular-Predominant)**	2,350	883 (37.6)	222	15.0	37.1 (27.7 to 49.8)	62.4	301 (12.8)	39.4%	17.6%	7.3%
**Phenotype 2 (Hypoalbuminemic-Anemic)**	2,484	1,181 (47.5)	138	31.0	125.5 (103.1 to 152.5)	52.5	232 (9.3)	27.5%	12.5%	6.7%
**Phenotype 3 (Low Comorbidity Burden)**	2,925	324 (11.1)	351	5.1	5.3 (5.0 to 5.6)	88.9	164 (5.6)	48.7%	36.4%	22.5%
**Phenotype 4 (High AF Chronicity)**	2,124	618 (29.1)	1,652	543.5	854.9 (776.1 to 910.8)	70.9	1,215 (57.2)	86.0%	69.8%	44.9%
**Phenotype 5 (Warfarin-Predominant Anticoagulation)**	2,178	608 (27.9)	291	12.8	16.6 (14.0 to 20.5)	72.1	253 (11.6)	43.0%	25.3%	14.8%
**Phenotype 6 (Renal Dysfunction)**	1,906	917 (48.1)	168	20.4	116.5 (80.4 to 171.2)	51.9	224 (11.8)	29.3%	10.5%	6.0%

Observed deaths are counts of deaths recorded at any time in the database and the percentages are observed-death proportions accrued over markedly unequal observation windows, not 1-year or

comparable risks. Reverse Kaplan-Meier median follow-up treats censoring as the event and death as censoring, and is the appropriate summary of observation time; the observed median follow-up column is the raw median of follow-up time and is shown for continuity with the submitted version of this table. Across the whole cohort the reverse Kaplan-Meier median follow-up is 43.1 days (95% CI 38.0 to 50.5), 67.6% of patients were censored, and 2,389 patients (17.1%) remained at risk at 1 year. Numbers at risk at seven time points are given in Supplementary Table S11 and the censoring diagnostics in Supplementary Table S12.

Supplementary Table S9. Cluster Selection Metrics for k = 4 to k = 8.

**Number of Clusters**	**4**	**5**	**6**	**7**	**8**
**Silhouette Coefficient**	0.1213	0.1351	0.1386	0.1378	0.1355
**Gap Statistic**	2.4481	2.4655	2.4748	2.4767	2.4698
**Gap Standard Error**	0.0039	0.0022	0.0032	0.0024	0.0023
**Calinski-Harabasz Index**	1,722.1	1,704.6	1,678.5	1,617.2	1,559.7
**Davies-Bouldin Index**	1.9139	1.7535	1.6357	1.5730	1.5683
**Within-Cluster Sum of Squares**	48,820	44,938	41,772	39,457	37,530
**Patients in Smallest Cluster**	2,997	2,394	1,906	1,324	936
**Patients in Largest Cluster**	3,906	3,538	2,925	2,338	2,536
**Size Ratio (Largest to Smallest)**	1.30	1.48	1.53	1.77	2.71
**Deaths in Smallest Cluster**	1,140	1,100	917	503	501
**Fewest Deaths in Any Cluster**	435	398	324	284	165
**Lowest Cluster Mortality (%)**	11.6	11.2	11.1	12.1	8.7
**Highest Cluster Mortality (%)**	48.1	47.1	48.1	51.9	53.5

**Mortality Range (Percentage Points)**	36.5	35.8	37.0	39.8	44.8
**Adjusted Rand Index vs Primary Solution**	0.509	0.701	1.000	0.691	0.515
**Bootstrap Adjusted Rand Index (Mean)**	0.889	0.930	0.955	0.926	0.904
**Bootstrap Adjusted Rand Index (SD)**	0.077	0.023	0.010	0.034	0.100

Metrics were computed on the primary variational autoencoder latent space (13,967 patients, seed 42). The four internal validity indices do not converge on k = 6: the silhouette coefficient is maximal at k = 6, the gap statistic at k = 7, the Calinski-Harabasz index at k = 4 and the Davies-Bouldin index at k = 8 (a lower Davies-Bouldin index is better). Across k = 4 to k = 8 the silhouette coefficient varies by only 0.017 and the gap statistic by 0.025 against standard errors of 0.002 to 0.004. Over the full k = 2 to k = 8 scan the gap statistic peaks at k = 2, and the Tibshirani one standard error rule selects k = 2. The mortality range is smallest at k = 5 and rises at every step thereafter to its maximum at the largest k tested, so a criterion based on mortality spread always prefers a larger k and cannot select an intermediate value; it is reported here for completeness and is not used to justify k = 6. Bootstrap adjusted Rand indices are the mean over 50 iterations of 80 percent subsampling with K-means refitting and nearest-centroid assignment of the full cohort, scored against that k's own full-data solution. Patients in the smallest cluster and the fewest deaths in any cluster need not refer to the same cluster. This table extends Supplementary Table S3, which reports the silhouette coefficient and inertia over the wider range k = 2 to k = 8. Canonical phenotype names attach to the k = 6 solution only; clusters at other values of k were characterized from z-score profiles and carry no phenotype name.

Supplementary Table S10. Bootstrap Stability of the Six-Phenotype Solution Under Three Resampling Schemes.

Quantity	K-means level (encoder held fixed)	End to end (encoder retrained)	Permuted null (end to end)
**Iterations**	100	100	50
**Pair-counting Jaccard, mean (SD)**	0.926 (0.018)	0.343 (0.045)	0.221 (0.035)
**Pair-counting Jaccard, 95% interval**	0.890 to 0.958	0.258 to 0.423	0.176 to 0.298
**Adjusted Rand index, mean (SD)**	0.954 (0.012)	0.406 (0.060)	0.199 (0.058)
**Adjusted Rand index, 95% interval**	0.930 to 0.974	0.284 to 0.511	0.120 to 0.327
**Matched Jaccard, mean (SD)**	0.961 (0.010)	0.516 (0.056)	0.378 (0.060)
**Matched Jaccard, 95% interval**	0.942 to 0.979	0.413 to 0.612	0.265 to 0.484
**Proportion of Ambiguous Clustering**	0.040	0.352	0.573
**Mean within-phenotype consensus**	0.961	0.511	0.466
**Mean between-phenotype consensus**	0.008	0.107	0.131
**Silhouette coefficient in replicates, mean (SD)**	0.1385 (0.0015)	0.1572 (0.0130)	0.1149 (0.0104)
**Matched Jaccard, Phenotype 1 (Cerebrovascular-Predominant)**	0.960 (0.011)	0.533 (0.098)	not applicable
**Matched Jaccard, Phenotype 2 (Hypoalbuminemic-Anemic)**	0.956 (0.017)	0.614 (0.095)	not applicable
**Matched Jaccard, Phenotype 3 (Low Comorbidity Burden)**	0.966 (0.010)	0.563 (0.086)	not applicable
**Matched Jaccard, Phenotype 4 (High**	0.962 (0.015)	0.507 (0.070)	not applicable

**AF Chronicity)**			
**Matched Jaccard, Phenotype 5 (Warfarin-Predominant Anticoagulation)**	0.977 (0.009)	0.435 (0.172)	not applicable
**Matched Jaccard, Phenotype 6 (Renal Dysfunction)**	0.948 (0.019)	0.446 (0.128)	not applicable
**Observed-death proportion range, median (95% interval), percentage points**	37.1 (36.2 to 38.2)	39.2 (34.6 to 45.2)	2.1 (0.9 to 3.8)
**Observed-death proportion range, minimum and maximum across replicates, percentage points**	35.9 to 38.7	18.8 to 47.2	0.5 to 4.4
**Spearman correlation of phenotype mortality with the primary solution, mean**	0.984	0.618	not applicable
**Replicates preserving the exact mortality rank order (%)**	74	16	not applicable

All three schemes resample 80 percent of the cohort. In the K-means level scheme the trained variational autoencoder is held fixed and only K-means is refit, so the scheme measures the reproducibility of the partition given a fixed learned representation. In the end-to-end scheme the standardizer and the variational autoencoder are refit from scratch on every subsample with the same selected hyperparameters and early stopping rule, K-means is refit in the resulting latent space, and the full cohort is assigned by nearest centroid. The permuted null repeats the end-to-end procedure on a dataset in which every feature

column was independently shuffled, preserving all marginal distributions while destroying the joint structure. Bootstrap clusters were matched to the primary solution by the Hungarian algorithm before scoring. The Proportion of Ambiguous Clustering is the fraction of patient pairs whose consensus value falls between 0.1 and 0.9, estimated from 5,000,000 sampled distinct patient pairs. The analytic chance value for the pair-counting Jaccard index, 1/(2k-1) = 0.091, is far below the value the identical pipeline reaches on permuted data, so the permuted null rather than the analytic constant is the operative benchmark. Neither scheme propagates uncertainty in model selection: the hyperparameters and k = 6 were held fixed throughout. The largest off-diagonal end-to-end co-assignment is 0.207, between Phenotype 5 (Warfarin-Predominant Anticoagulation) and Phenotype 6 (Renal Dysfunction), which is where the partition is softest; the next largest is 0.153, between Phenotype 1 (Cerebrovascular-Predominant) and Phenotype 4 (High AF Chronicity). The primary solution has a silhouette coefficient of 0.1386 and a mortality range of 37.0 percentage points. Two permuted-null silhouette coefficients are reported in this package and they are not interchangeable. The value in the row above, 0.1149 (standard deviation 0.0104), is the mean across the permuted-null resampling replicates. The value quoted in the Discussion, 0.1185, is the single reference partition obtained by running the whole pipeline once on the permuted cohort, which is the partition every permuted-null replicate is scored against. Mortality figures are observed-death proportions accrued over markedly unequal observation windows and are not comparable risks.

Supplementary Table S11. Numbers at Risk by Phenotype and Follow-Up Time.

**Phenotype**	**Cluster ID**	**Patients**	**At risk at day 0**	**At risk at day 30**	**At risk at day 90**	**At risk at day 180**	**At risk at day 365**	**At risk at day 730**	**At risk at day 1095**

**Phenotype 1 (Cerebrovascular-Predominant)**	0	2,350	2,350	940	661	494	301	125	56
**Phenotype 2 (Hypoalbuminemic-Anemic)**	1	2,484	2,484	1,261	771	514	232	96	44
**Phenotype 3 (Low Comorbidity Burden)**	2	2,925	2,925	592	442	324	164	63	20
**Phenotype 4 (High AF Chronicity)**	3	2,124	2,124	1,673	1,534	1,422	1,215	906	621
**Phenotype 5 (Warfarin-Predominant Anticoagulation)**	4	2,178	2,178	813	561	417	253	116	64
**Phenotype 6 (Renal Dysfunction)**	5	1,906	1,906	854	600	431	224	93	37
**All patients**		13,967	13,967	6,133	4,569	3,602	2,389	1,399	842

Time zero is the admission time of the index encounter, defined as the first hospital admission carrying an ICD-10 I48 atrial fibrillation diagnosis code. A patient is counted as at risk at a given day if follow-up time is at least that many days. Cluster identifiers are the published integer labels of the primary solution and are not comparable with the integer labels used in any other analysis; join on the phenotype name. Analyses that require follow-up strictly greater than the start of an interval give slightly smaller counts,

6,103 rather than 6,133 at day 30 and 2,383 rather than 2,389 at day 365 (Supplementary Table S14); both conventions are correct and neither is an error.

Supplementary Table S12. Censoring Diagnostics by Phenotype.

**Phenotype**	**Patients**	**Censored, n (%)**	**Censoring Hazard Ratio versus Phenotype 4 (95% CI)**	**Single Admission, n (%)**	**Single Admission and Censored Alive, n (%)**
**Phenotype 1 (Cerebrovascular-Predominant)**	2,350	1,467 (62.4)	2.30 (2.13 to 2.48)	1,195 (50.9)	770 (32.8)
**Phenotype 2 (Hypoalbuminemic-Anemic)**	2,484	1,303 (52.5)	1.76 (1.63 to 1.91)	1,171 (47.1)	635 (25.6)
**Phenotype 3 (Low Comorbidity Burden)**	2,925	2,601 (88.9)	5.62 (5.24 to 6.02)	1,998 (68.3)	1,788 (61.1)
**Phenotype 4 (High AF Chronicity)**	2,124	1,506 (70.9)	1.00 (reference)	360 (16.9)	275 (12.9)
**Phenotype 5 (Warfarin-Predominant Anticoagulation)**	2,178	1,570 (72.1)	2.71 (2.52 to 2.92)	1,212 (55.6)	899 (41.3)
**Phenotype 6 (Renal**	1,906	989 (51.9)	1.86 (1.71 to	991 (52.0)	537 (28.2)

**Dysfunction)**			2.02)		

Patients not known to have died were censored at the latest discharge time recorded across all of their encounters, so censoring time is structurally a measure of healthcare utilization. Censoring hazard ratios come from a Cox model of the censoring hazard on phenotype with Phenotype 4 (High AF Chronicity) as the reference; a ratio above 1 means that phenotype is censored faster, that is followed for a shorter time, than the reference (concordance 0.674, likelihood ratio test $p < 0.001$). The censoring distributions differ across phenotypes (reverse Kaplan-Meier log-rank chi-square 3,213.0 on 5 degrees of freedom, $p < 0.001$), and 14 of the 15 pairwise reverse Kaplan-Meier comparisons are significant at the Bonferroni threshold of 0.0033; the exception is Phenotype 2 (Hypoalbuminemic-Anemic) versus Phenotype 6 (Renal Dysfunction), $p = 0.28$. Among censored patients, follow-up time is strongly rank-correlated with cumulative length of stay (Spearman rho = 0.73) and with the number of encounters (Spearman rho = 0.64), both $p < 0.001$. Across the whole cohort 6,927 patients (49.6%) had a single admission and 4,904 (35.1%) had a single admission and were censored alive; for the latter group follow-up time is identically the index length of stay (Pearson r = 0.9999). Censoring in this cohort is therefore informative, and it is driven by the same utilization variables that contribute to phenotype definition.

Supplementary Table S13. Landmark Analyses at 30 and 90 Days.

Landmark	Phenotype	Patients Retained, n (%)	Deaths After Landmark	Mortality After Landmark (%)	Reverse Kaplan-Meier Median Follow-Up From	Hazard Ratio versus Phenotype 4 (95% CI)

					Landmark (Days)	
**30 days**	Phenotype 1 (Cerebrovascular-Predominant)	937 (39.9)	516	55.1	548.4	3.75 (3.32 to 4.24)
**30 days**	Phenotype 2 (Hypoalbuminemic-Anemic)	1,249 (50.3)	710	56.8	358.6	5.16 (4.61 to 5.79)
**30 days**	Phenotype 3 (Low Comorbidity Burden)	589 (20.1)	220	37.4	346.5	2.71 (2.31 to 3.17)
**30 days**	Phenotype 4 (High AF Chronicity)	1,670 (78.6)	586	35.1	1,108.9	1.00 (reference)
**30 days**	Phenotype 5 (Warfarin-Predominant Anticoagulation)	807 (37.1)	396	49.1	496.2	3.25 (2.86 to 3.71)
**30 days**	Phenotype 6 (Renal Dysfunction)	851 (44.6)	562	66.0	610.2	4.90 (4.35 to 5.53)
**90 days**	Phenotype 1 (Cerebrovascular-Predominant)	660 (28.1)	368	55.8	631.6	3.46 (3.02 to 3.96)
**90 days**	Phenotype 2 (Hypoalbuminemic-Anemic)	767 (30.9)	437	57.0	484.9	4.51 (3.96 to 5.14)

**90 days**	Phenotype 3 (Low Comorbidity Burden)	441 (15.1)	168	38.1	376.6	2.77 (2.32 to 3.30)
**90 days**	Phenotype 4 (High AF Chronicity)	1,534 (72.2)	538	35.1	1,129.2	1.00 (reference)
**90 days**	Phenotype 5 (Warfarin-Predominant Anticoagulation)	561 (25.8)	292	52.0	581.9	3.06 (2.65 to 3.53)
**90 days**	Phenotype 6 (Renal Dysfunction)	597 (31.3)	401	67.2	769.9	4.67 (4.09 to 5.33)

Patients alive and under observation at the landmark, that is with follow-up time strictly greater than the landmark, are retained; time zero is reset to the landmark and follow-up is measured forward from it. At the 30-day landmark 6,103 patients (43.7%) were retained and 2,990 deaths were observed (log-rank chi-square 1,106.5 on 5 degrees of freedom, $p < 0.001$); at the 90-day landmark 4,560 patients (32.6%) were retained and 2,204 deaths were observed (log-rank chi-square 812.6 on 5 degrees of freedom, $p < 0.001$). Hazard ratios are unadjusted and are expressed against Phenotype 4 (High AF Chronicity); all are $p < 0.001$. Mortality after the landmark is an observed-death proportion over unequal follow-up, not a risk.

Supplementary Table S14. Period-Specific Hazard Ratios for All-Cause Mortality.

**Follow-Up Period**	**Phenotype**	**Patients Contributing**	**Deaths Contributing**	**Hazard Ratio (95% CI)**	**P Value**
**0 to 30 days**	Phenotype 1 (Cerebrovascular-	2,350	367	13.88 (9.67 to	< 0.001

	Predominant)			19.92)	
**0 to 30 days**	Phenotype 2 (Hypoalbuminemic-Anemic)	2,484	471	16.96 (11.85 to 24.27)	< 0.001
**0 to 30 days**	Phenotype 3 (Low Comorbidity Burden)	2,925	104	6.67 (4.48 to 9.93)	< 0.001
**0 to 30 days**	Phenotype 4 (High AF Chronicity)	2,124	32	1.00 (reference)	
**0 to 30 days**	Phenotype 5 (Warfarin-Predominant Anticoagulation)	2,178	212	10.14 (6.99 to 14.70)	< 0.001
**0 to 30 days**	Phenotype 6 (Renal Dysfunction)	1,906	355	16.10 (11.21 to 23.13)	< 0.001
**30 to 365 days**	Phenotype 1 (Cerebrovascular-Predominant)	937	370	4.70 (3.94 to 5.60)	< 0.001
**30 to 365 days**	Phenotype 2 (Hypoalbuminemic-Anemic)	1,249	599	9.93 (8.42 to 11.72)	< 0.001
**30 to 365 days**	Phenotype 3 (Low Comorbidity Burden)	589	195	5.59 (4.57 to 6.84)	< 0.001
**30 to 365 days**	Phenotype 4 (High AF Chronicity)	1,670	189	1.00 (reference)	
**30 to 365 days**	Phenotype 5 (Warfarin-Predominant Anticoagulation)	807	300	5.20 (4.33 to 6.24)	< 0.001
**30 to 365 days**	Phenotype 6 (Renal Dysfunction)	851	434	6.57 (5.53 to 7.80)	< 0.001

**Beyond 365 days**	Phenotype 1 (Cerebrovascular-Predominant)	300	146	2.83 (2.33 to 3.43)	< 0.001
**Beyond 365 days**	Phenotype 2 (Hypoalbuminemic-Anemic)	231	111	4.32 (3.47 to 5.37)	< 0.001
**Beyond 365 days**	Phenotype 3 (Low Comorbidity Burden)	163	25	1.90 (1.26 to 2.88)	0.002
**Beyond 365 days**	Phenotype 4 (High AF Chronicity)	1,215	397	1.00 (reference)	
**Beyond 365 days**	Phenotype 5 (Warfarin-Predominant Anticoagulation)	251	96	2.48 (1.97 to 3.11)	< 0.001
**Beyond 365 days**	Phenotype 6 (Renal Dysfunction)	223	128	3.95 (3.22 to 4.85)	< 0.001

The primary Cox model, adjusted for age at first atrial fibrillation admission and for sex, was refitted separately within three disjoint follow-up intervals because the proportional hazards assumption was rejected in every model fitted (Supplementary Table S16). Hazard ratios are expressed against Phenotype 4 (High AF Chronicity). Between the first 30 days and the period beyond 1 year the estimates attenuate 3.5 fold (Phenotype 3, Low Comorbidity Burden) to 4.9 fold (Phenotype 1, Cerebrovascular-Predominant). The rank ordering of the phenotypes matches the pooled model in the first and last intervals; in the 30 to 365 day interval Phenotype 1 (Cerebrovascular-Predominant) and Phenotype 3 (Low Comorbidity Burden) exchange positions. Patients at risk at the start of each interval are 13,967, 6,103 and 2,383 and deaths within each interval are 1,541, 2,087 and 903. An interval requires follow-up strictly greater than its start, which is why these counts are slightly smaller than the at-least-that-day

counts in Supplementary Table S11. This table is the numerical source for panel B of Figure 5, which prints no numerals; the pooled estimates plotted in panel A are given in Table 2 of the main text.

Supplementary Table S15. Variance Inflation Factors for the Adjusted Cox Models.

Term	Primary Model (Age and Sex), VIF	Primary Model, Tolerance	Secondary Model (Age, Sex, CHA2DS2-VASc), VIF	Secondary Model, Tolerance
**Phenotype 1 (Cerebrovascular-Predominant)**	1.76	0.568	1.89	0.530
**Phenotype 2 (Hypoalbuminemic-Anemic)**	1.81	0.552	1.91	0.522
**Phenotype 3 (Low Comorbidity Burden)**	1.95	0.513	2.23	0.448
**Phenotype 5 (Warfarin-Predominant Anticoagulation)**	1.72	0.582	1.74	0.577
**Phenotype 6 (Renal Dysfunction)**	1.65	0.605	1.66	0.603
**Age at first atrial fibrillation admission, per year**	1.13	0.884	1.46	0.688
**Female sex**	1.06	0.940	1.17	0.859
**CHA2DS2-VASc score, per point**	not in model	not in model	2.37	0.422

**Maximum variance inflation factor**	1.95		2.37	

Variance inflation factors were computed on the Cox design matrix (an intercept plus the model terms), which is the standard construction; the Cox partial likelihood carries no intercept, so the intercept is used only to make each auxiliary regression the conventional centered one. Phenotype 4 (High AF Chronicity) is the reference category and has no indicator term. A variance inflation factor of v means the variance of the estimated coefficient is v times larger than it would be if that term were uncorrelated with the other terms. No term exceeds any conventional threshold: none is above 10 (serious), above 5 (warrants inspection), or above the conservative alternative of 2.5. Variance inflation is therefore not the mechanism of concern in these models. The concern is instead that the composite CHA2DS2-VASc score and all eight of its component terms were supplied to the clustering model, as were age and sex, so no adjustment set available in this design is fully independent of the exposure. The CHA2DS2-VASc term also behaves incoherently once age is in the model, moving from a hazard ratio of 1.069 per point when fitted alone to 0.955 per point (95% CI 0.932 to 0.977) when fitted alongside age, sex and phenotype, and it raises concordance by only 0.002. Estimates from that model are therefore reported as a secondary analysis.

Supplementary Table S16. Proportional Hazards Assessment by Schoenfeld Residual Test.

Test or Covariate	Rank Transform Chi-Square	df	Rank Transform P	Kaplan-Meier Transform Chi-Square	Kaplan-Meier Transform P	Log Hazard Ratio Over Time
**Global test, Model 1**	116.0	5	< 0.001	144.2	< 0.001	

**(unadjusted)**						
**Global test, Model 2 (age and sex, primary)**	140.6	7	< 0.001	157.6	< 0.001	
**Global test, Model 3 (age, sex, CHA2DS2-VASc, secondary)**	178.2	8	< 0.001	198.8	< 0.001	
**Phenotype 1 (Cerebrovascular-Predominant)**	93.37	1	< 0.001	113.67	< 0.001	Decreases with time
**Phenotype 2 (Hypoalbuminemic-Anemic)**	32.66	1	< 0.001	58.37	< 0.001	Decreases with time
**Phenotype 3 (Low Comorbidity Burden)**	2.96	1	0.085	10.31	0.001	Decreases with time
**Phenotype 5 (Warfarin-Predominant Anticoagulation)**	38.17	1	< 0.001	58.55	< 0.001	Decreases with time
**Phenotype 6 (Renal Dysfunction)**	64.50	1	< 0.001	80.52	< 0.001	Decreases with time
**Age at first atrial fibrillation admission, per year**	30.04	1	< 0.001	26.05	< 0.001	Increases with time
**Female sex**	0.02	1	0.890	0.11	0.739	Decreases with time

Schoenfeld residual tests (Grambsch and Therneau) were computed under two time transforms, rank and Kaplan-Meier, for all three Cox models; per-covariate results are shown for the primary model adjusted for age and sex. The assumption is rejected globally in every model under either transform. Under the rank transform 5 of 7 covariates in the primary model violate the assumption at the 0.05 level and the same 5 at the Bonferroni threshold of 0.0071. Every phenotype log hazard ratio decreases with follow-up time while the age effect increases, so each pooled hazard ratio is an average of a relative hazard that falls as follow-up lengthens rather than a constant multiplier; the period-specific estimates are given in Supplementary Table S14. This assessment was added in revision. The submitted manuscript and supplement contained no proportional hazards testing, and the violation overturns no submitted conclusion, since the ordering and the significance of the phenotype effects hold within every interval.

Supplementary Table S17. Discrimination for All-Cause Mortality Across Nested Cox Models.

Analysis	Model or Comparison	Covariates or Terms Added	Apparent C-Index (95% CI)	Optimism-Corrected C-Index (95% CI)	Cross-Validated C-Index (95% CI)	Likelihood Ratio Chi-Square (df)
**Discrimination**	M0	Phenotype alone	0.664 (0.652 to 0.671)	0.663 (0.655 to 0.671)	0.686 (0.676 to 0.696)	
**Discrimination**	M1	CHA2DS2-VASc alone	0.533 (0.522 to 0.543)	0.533 (0.523 to 0.544)	0.533 (0.528 to 0.538)	
**Discrimination**	M2	Age, sex, CHA2DS2-	0.603	0.603	0.603	

**ation**		VASc	(0.594 to 0.613)	(0.594 to 0.612)	(0.598 to 0.608)	
**Discrimination**	M2p	Age, sex, CHA2DS2-VASc, phenotype	0.701 (0.694 to 0.710)	0.700 (0.692 to 0.708)	0.709 (0.703 to 0.716)	
**Discrimination**	M3	Age, sex, CHA2DS2-VASc, routine labs	0.683 (0.673 to 0.692)	0.682 (0.673 to 0.692)	0.682 (0.676 to 0.688)	
**Discrimination**	M4	Age, sex, CHA2DS2-VASc, routine labs, phenotype	0.733 (0.725 to 0.742)	0.732 (0.724 to 0.740)	0.741 (0.736 to 0.747)	
**Discrimination**	M5	Age, sex, CHA2DS2-VASc, routine labs, utilization	0.745 (0.737 to 0.754)	0.744 (0.736 to 0.752)	0.744 (0.739 to 0.750)	
**Discrimination**	M6	Age, sex, CHA2DS2-VASc, routine labs, utilization, phenotype	0.766 (0.759 to 0.774)	0.765 (0.757 to 0.772)	0.769 (0.763 to 0.775)	
**Discrimination**	M4 leaky	M4 with full-cohort phenotype labels applied to held-out patients	0.733	not estimated	0.732 (0.726 to 0.739)	
**Increment**	M1 to M2	Age at first atrial fibrillation admission,	0.070 (0.060 to	0.070 (0.058 to	0.070 (0.064 to	556.4 (2 df)

		sex	0.082)	0.081)	0.077)	
**Increment**	M2 to M3	Creatinine, hemoglobin, albumin	0.079 (0.069 to 0.089)	0.080 (0.071 to 0.089)	0.079 (0.075 to 0.083)	1,256.7 (3 df)
**Increment**	M3 to M4	Phenotype, as five indicator terms with Phenotype 4 (High AF Chronicity) as the reference	0.051 (0.046 to 0.056)	0.050 (0.045 to 0.055)	0.059 (0.056 to 0.062)	1,244.5 (5 df)
**Increment**	M2 to M2p	Phenotype, as five indicator terms with Phenotype 4 (High AF Chronicity) as the reference	0.098 (0.090 to 0.104)	0.098 (0.091 to 0.104)	0.106 (0.101 to 0.112)	1,869.9 (5 df)
**Increment**	M5 to M6	Phenotype, as five indicator terms with Phenotype 4 (High AF Chronicity) as the reference	0.022 (0.018 to 0.025)	0.021 (0.018 to 0.024)	0.025 (0.022 to 0.028)	857.6 (5 df)

Harrell's concordance index for all-cause mortality in 13,967 patients with 4,531 deaths. Apparent values are the full-cohort fit evaluated on the full cohort. Optimism-corrected values use Harrell's bootstrap with 500 resamples, refitting every model on each resample, evaluating it on the resample and on the original cohort, averaging the difference and subtracting it from the apparent value; optimism was negligible for

every model, at most 0.0008 concordance units. Cross-validated values use three repeats of stratified five-fold cross-validation in which the standardizer, the variational autoencoder and the K-means solution were refitted from scratch inside each training fold and validation patients were assigned to the nearest training-fold centroid, giving 15 independent refits of the representation and the clustering. The row labeled M4 leaky repeats those folds while assigning held-out patients the phenotype labels derived from the full cohort; it gives a lower cross-validated concordance (0.732) than the honest fold-wise re-derivation (0.741), so information leakage does not explain the phenotype's contribution. The phenotype-alone model has a fitted concordance of 0.6635, which is 0.664 on the apparent scale and 0.663 after optimism correction. A conventional model containing age, sex, CHA2DS2-VASc, the three routine laboratory values and three utilization variables, with no phenotype term (M5), reaches an apparent concordance of 0.745, which exceeds the phenotype model M4 at 0.733. Routine laboratory values are creatinine, hemoglobin and albumin, observed in 99.6%, 99.5% and 79.6% of patients and k-nearest-neighbor imputed during feature engineering, so no patient was excluded. Because censoring in this cohort is informative and follow-up is severely unequal across phenotypes, these discrimination statistics are reported as descriptive rather than as unbiased measures.

Supplementary Table S18. Re-Clustering Using Baseline Clinical Variables Only.

**Quantity**	**Primary Published Solution**	**Refit Control (Unchanged Feature Set)**	**Variant 1, Utilization Removed (BO1)**	**Variant 2, Utilization and Encounter-Derived Removed (BO2)**
**Variables in the feature set**	35	35	32	30

**Model refits**	not applicable	10	10	10
**Adjusted Rand index versus the primary partition, mean (SD)**	1.000 (reference)	0.400 (0.070)	0.438 (0.054)	0.382 (0.077)
**Adjusted Rand index versus the primary partition, range across refits**	not applicable	0.344 to 0.514	0.371 to 0.565	0.246 to 0.485
**Adjusted mutual information versus the primary partition, mean (SD)**	1.000 (reference)	0.430 (0.050)	0.449 (0.042)	0.406 (0.062)
**Mean pairwise adjusted Rand index between refits of the same solution**	not applicable	0.480	0.569	0.424
**Silhouette coefficient, mean (SD)**	0.1386	0.151 (0.014)	0.144 (0.013)	0.138 (0.010)
**Observed-death proportion, lowest to highest cluster (%)**	11.1 to 48.1	not reported	8.5 to 48.4	10.6 to 53.9
**Mortality range (percentage points)**	37.0	not reported	39.9	43.3
**Global log-rank chi-square (5 df)**	1,594.7	not reported	2,604.7	909.1
**Unadjusted Cox concordance**	0.663	not reported	0.698	0.632
**Median cumulative length of stay, lowest to highest cluster (days)**	4.6 to 20.0	not reported	5.8 to 25.6	6.2 to 14.5

**Length of stay, fold difference between the extreme clusters**	4.3	not reported	4.4	2.3
**Intensive care admission, lowest to highest cluster (%)**	33.6 to 77.8	not reported	48.2 to 73.3	50.3 to 68.5
**Intensive care admission range (percentage points)**	44.2	not reported	25.1	18.2
**Share of each published phenotype assigned to a single cluster of this solution (%)**	100	not reported	55.4 to 86.6	36.7 to 81.1

The entire representation learning and clustering procedure was repeated on reduced feature sets with the same architecture, the same selected hyperparameters (latent dimension 10, beta 0.5, learning rate 0.0005), the same early stopping rule and k = 6. Variant 1 (BO1) removes cumulative length of stay, number of encounters and any intensive care admission; variant 2 (BO2) additionally removes the number of atrial fibrillation coded encounters and the interval between the first and last atrial fibrillation coded admission. The refit control repeats the unchanged 35-variable feature set ten times with the training and validation split pinned at seed 42 and only the network initialization varied, so it isolates refit instability from the feature change. Because agreement between two refits of the same specification is itself well below one, the agreement of each variant must be read against the refit control rather than against complete agreement: variant 1 reaches 0.438 against a control of 0.400, and the two distributions overlap entirely (Mann-Whitney U = 72.0, p = 0.104). Two further constructions of the same benchmark agree with it, 0.432 when the split is also allowed to vary (Supplementary Table S19) and 0.406 for an end-to-end bootstrap that additionally resamples patients (Supplementary Table S10), so refitting this pipeline on unchanged data reproduces the published partition at an adjusted Rand index of roughly 0.40 to 0.43 depending on which sources of randomness are held fixed. Clusters of the two variants are labeled BO1-A

to BO1-F and BO2-A to BO2-F and are never given a phenotype name. Observed-death proportions accrue over unequal follow-up; in variant 1 the reverse Kaplan-Meier median follow-up still ranges from 6.5 to 1,264.5 days across clusters, so censoring remains structurally tied to utilization even after the utilization variables are removed from the model. Full six by six cross-tabulations with row and column percentages for both variants are shown in Supplementary Figure S11.

Supplementary Table S19. Index-Admission Encoding: Agreement With the Primary Partition.

Comparison	Variables	Encoding	Refits	Adjusted Rand Index, Mean (Range)
**Refit control (arm C) versus the primary partition**	35	Cumulative	5	0.432 (0.384 to 0.513)
**Feature-restriction control (arm R) versus the primary partition**	28	Cumulative	5	0.239 (0.219 to 0.268)
**Index-admission solution (arm I) versus the primary partition**	28	Index admission	5	0.159 (0.148 to 0.165)
**Index-admission solution versus the feature-restriction control, matched seeds**	28	Index admission versus cumulative	5	0.291 (0.245 to 0.359)
**Adjusted Rand index lost by dropping the seven features without an index-admission counterpart**				0.193 (71% of the total drop)
**Adjusted Rand index lost by re-**				0.080 (29% of the

**encoding the remaining 28 features**				total drop)
**End-to-end bootstrap of the unchanged feature set (Supplementary Table S10)**	35	Cumulative	100 iterations	0.406 (SD 0.060)

Every comorbidity, medication, clinical score and laboratory variable was recomputed using only data recorded at the index admission, with identical International Classification of Diseases code sets, identical medication keyword lists and identical score component definitions; the only difference between the two encodings is the unit of aggregation. Seven features have no index-admission counterpart and were excluded from both the index arm and its matched control: total encounters, the atrial fibrillation coded admission interval and the number of atrial fibrillation coded encounters are counts or intervals defined over the whole record, and potassium, sodium, albumin and the international normalized ratio are not carried at admission level in the derived dataset. That leaves 28 features. All three arms retrain the variational autoencoder from scratch and vary both the random initialization and the training and validation split with the seed. The index-admission solution reported in the text is the seed 42 solution (adjusted Rand index 0.148, adjusted mutual information 0.187, silhouette coefficient 0.1471 against 0.1386 for the primary solution). The index-admission value must be read against the refit control rather than against complete agreement, and the 0.291 figure must be read against the matched within-arm control of 0.533, since 0.291 compares one refit with another refit while 0.432 compares a refit with the single published partition. The permuted-data null of 0.199 is not a valid comparator here: it is the agreement of one specification with itself under resampling of structureless data, not a floor for agreement between two different specifications. Clusters of the index-admission solution are labeled IA-A to IA-F and are never given a phenotype name.

Supplementary Table S20. Index-Admission Encoding: Effect on Each Phenotype.

Phenotype	Patients	Largest Share Assigned to One Index-Admission Cluster (%)	Matched Jaccard, Index-Admission Encoding	Matched Jaccard, End-to-End Refit	Mean Absolute Shift in Feature Prevalence (Percentage Points)	Chronic Kidney Disease, Cumulative Then Index (%)	Longstanding Persistent or Permanent AF, Cumulative Then Index (%)
**Phenotype 1 (Cerebrovascular-Predominant)**	2,350	57.5 (IA-F)	0.490	0.533	4.98	22.9 then 19.5	0.5 then 0.4
**Phenotype 2 (Hypoalbuminemic-Anemic)**	2,484	45.7 (IA-D)	0.332	0.614	4.32	19.6 then 15.8	1.0 then 0.7
**Phenotype 3 (Low Comorbidity Burden)**	2,925	35.5 (IA-A)	0.210	0.563	1.61	4.0 then 3.5	0.1 then 0.1
**Phenotype 4 (High AF Chronicity)**	2,124	50.3 (IA-B)	0.270	0.507	10.80	39.5 then 25.1	19.0 then 9.7
**Phenotype 5 (Warfarin-**	2,178	25.3 (IA-C)	0.141	0.435	4.18	31.8 then 26.9	1.1 then 1.1

**Predominant Anticoagulation)**							
**Phenotype 6 (Renal Dysfunction)**	1,906	51.0 (IA-E)	0.347	0.446	4.28	79.2 then 74.8	1.6 then 1.0

Membership robustness is the share of each published phenotype whose patients remain together in a single index-admission cluster, and the matched Jaccard index after optimal matching of the two partitions. The end-to-end refit column is the matched Jaccard obtained for the same phenotype when the unchanged feature set is refit (Supplementary Table S10) and is the fair yardstick, since some loss of agreement follows from refitting alone. Across the 18 comorbidity and medication flags the cumulative encoding raises prevalence by a mean of 4.96 percentage points (median 4.92, maximum 9.24 for angiotensin converting enzyme inhibitors or angiotensin receptor blockers), a mean relative inflation of 1.22. The inflation is entirely a function of record length: among the 6,927 patients with a single admission all 18 flags are identical under the two encodings, whereas among the 7,040 patients with more than one admission the mean inflation is 9.84 percentage points. Cohort-wide, chronic kidney disease falls from 29.96% (4,185 patients) to 25.05% (3,499), so 686 patients (16.4% of those ever coded) carry the diagnosis only from a non-index admission, and of the 499 patients ever coded longstanding persistent or permanent atrial fibrillation only 280 (56.1%) are still coded that way at the index admission. Continuous features move in both directions: the mean CHA2DS2-VASc score is 4.21 under cumulative encoding and 3.92 at the index admission and the mean HAS-BLED score 3.54 and 3.10, whereas the mean creatinine is 1.254 and 1.370 and the mean hemoglobin 10.76 and 11.21, because the index admission is an acute event while cumulative encoding takes a median across every admission. Reviewer 1 predicted that the phenotypes defined by chronic kidney disease and by atrial fibrillation chronicity would be the ones most affected by this encoding. The prediction holds for Phenotype 4 (High AF Chronicity), which has the largest composition shift of any phenotype by more than a factor of two and

whose defining chronicity code halves within it. It does not hold for Phenotype 6 (Renal Dysfunction), the second most robust phenotype by membership, which is anchored by creatinine measured at the index admission rather than by a cumulative flag; a renal cluster with the highest observed-death proportion of the six (49.8%) re-emerges under index-admission encoding. The two least reproducible phenotypes are instead Phenotype 5 (Warfarin-Predominant Anticoagulation), which is defined largely by a cumulative medication flag, and Phenotype 3 (Low Comorbidity Burden), which is defined by the absence of comorbidity and is therefore the most sensitive to which patients lose flags. The observed-death gradient survives the re-encoding at 36.2 percentage points across the six index-admission clusters against 37.0 percentage points in the primary solution.

Supplementary Table S21. Oral Anticoagulant Exposure by Anchor Year Group.

**Stratum**	**Measure**	**2014 to 2016, % (95% CI)**	**2017 to 2019, % (95% CI)**	**2020 to 2022, % (95% CI)**	**Trend P**
**Overall cohort**	Warfarin, ever	37.3 (35.8 to 38.8)	26.4 (25.3 to 27.6)	14.4 (13.4 to 15.4)	< 0.001
**Overall cohort**	DOAC, ever	38.0 (36.6 to 39.5)	41.7 (40.4 to 43.0)	48.9 (47.4 to 50.4)	< 0.001
**Overall cohort**	Any oral anticoagulant, ever	65.1 (63.6 to 66.5)	61.7 (60.4 to 62.9)	59.7 (58.3 to 61.1)	< 0.001
**Overall cohort**	No oral anticoagulant	34.9 (33.5 to 36.4)	38.3 (37.1 to 39.6)	40.3 (38.9 to 41.7)	< 0.001
**Phenotype 1**	Warfarin, ever	28.2 (25.0 to	14.4 (12.3 to	6.6 (5.0 to	<

**(Cerebrovascular-Predominant)**		31.6)	16.8)	8.7)	0.001
**Phenotype 1 (Cerebrovascular-Predominant)**	DOAC, ever	39.6 (36.1 to 43.3)	45.2 (42.0 to 48.4)	46.4 (42.7 to 50.1)	0.010
**Phenotype 1 (Cerebrovascular-Predominant)**	Any oral anticoagulant, ever	57.1 (53.5 to 60.7)	54.7 (51.5 to 57.9)	50.2 (46.5 to 53.9)	0.009
**Phenotype 1 (Cerebrovascular-Predominant)**	No oral anticoagulant	42.9 (39.3 to 46.5)	45.3 (42.1 to 48.5)	49.8 (46.1 to 53.5)	0.009
**Phenotype 2 (Hypoalbuminemic-Anemic)**	Warfarin, ever	15.7 (13.0 to 18.8)	10.6 (8.8 to 12.8)	5.4 (4.1 to 7.0)	< 0.001
**Phenotype 2 (Hypoalbuminemic-Anemic)**	DOAC, ever	28.7 (25.2 to 32.5)	39.0 (36.0 to 42.2)	50.9 (47.7 to 54.1)	< 0.001
**Phenotype 2 (Hypoalbuminemic-Anemic)**	Any oral anticoagulant, ever	39.9 (36.1 to 43.9)	44.6 (41.4 to 47.8)	53.8 (50.6 to 56.9)	< 0.001
**Phenotype 2 (Hypoalbuminemic-Anemic)**	No oral anticoagulant	60.1 (56.1 to 63.9)	55.4 (52.2 to 58.6)	46.2 (43.1 to 49.4)	< 0.001

**Phenotype 3 (Low Comorbidity Burden)**	Warfarin, ever	3.1 (2.1 to 4.7)	2.1 (1.5 to 3.1)	0.9 (0.5 to 1.7)	< 0.001
**Phenotype 3 (Low Comorbidity Burden)**	DOAC, ever	41.1 (37.5 to 44.8)	43.2 (40.3 to 46.0)	46.7 (43.7 to 49.7)	0.017
**Phenotype 3 (Low Comorbidity Burden)**	Any oral anticoagulant, ever	42.7 (39.0 to 46.3)	44.9 (42.0 to 47.7)	47.3 (44.4 to 50.4)	0.050
**Phenotype 3 (Low Comorbidity Burden)**	No oral anticoagulant	57.3 (53.7 to 61.0)	55.1 (52.3 to 58.0)	52.7 (49.6 to 55.6)	0.050
**Phenotype 4 (High AF Chronicity)**	Warfarin, ever	38.4 (35.0 to 41.9)	25.0 (22.1 to 28.2)	12.6 (10.1 to 15.5)	< 0.001
**Phenotype 4 (High AF Chronicity)**	DOAC, ever	76.3 (73.2 to 79.1)	81.9 (79.1 to 84.5)	84.2 (81.0 to 86.9)	< 0.001
**Phenotype 4 (High AF Chronicity)**	Any oral anticoagulant, ever	90.3 (88.1 to 92.2)	89.7 (87.4 to 91.7)	90.2 (87.5 to 92.3)	0.886
**Phenotype 4 (High AF Chronicity)**	No oral anticoagulant	9.7 (7.8 to 11.9)	10.3 (8.3 to 12.6)	9.8 (7.7 to 12.5)	0.886
**Phenotype 5 (Warfarin-Predominant Anticoagulation)**	Warfarin, ever	93.9 (92.0 to 95.3)	89.8 (87.6 to 91.6)	81.5 (77.8 to 84.7)	< 0.001
**Phenotype 5 (Warfarin-Predominant**	DOAC, ever	10.1 (8.2 to 12.4)	10.5 (8.7 to 12.7)	15.8 (12.8 to 19.3)	0.004

**Anticoagulation)**					
**Phenotype 5 (Warfarin-Predominant Anticoagulation)**	Any oral anticoagulant, ever	94.5 (92.7 to 95.9)	92.0 (90.1 to 93.6)	87.3 (84.0 to 89.9)	< 0.001
**Phenotype 5 (Warfarin-Predominant Anticoagulation)**	No oral anticoagulant	5.5 (4.1 to 7.3)	8.0 (6.4 to 9.9)	12.7 (10.1 to 16.0)	< 0.001
**Phenotype 6 (Renal Dysfunction)**	Warfarin, ever	31.7 (27.7 to 36.0)	25.0 (22.1 to 28.3)	9.2 (7.3 to 11.7)	< 0.001
**Phenotype 6 (Renal Dysfunction)**	DOAC, ever	27.5 (23.7 to 31.7)	34.6 (31.3 to 38.1)	45.2 (41.5 to 49.0)	< 0.001
**Phenotype 6 (Renal Dysfunction)**	Any oral anticoagulant, ever	51.1 (46.7 to 55.6)	53.4 (49.8 to 56.9)	50.9 (47.1 to 54.7)	0.851
**Phenotype 6 (Renal Dysfunction)**	No oral anticoagulant	48.9 (44.4 to 53.3)	46.6 (43.1 to 50.2)	49.1 (45.3 to 52.9)	0.851
**Overall cohort**	Warfarin only, no DOAC	27.0 (25.7 to 28.4)	20.0 (18.9 to 21.1)	10.8 (9.9 to 11.8)	< 0.001
**Overall cohort**	DOAC only, no warfarin	27.8 (26.4 to 29.2)	35.2 (34.0 to 36.5)	45.3 (43.9 to 46.8)	< 0.001
**Overall cohort**	Both warfarin and a DOAC	10.3 (9.4 to 11.2)	6.5 (5.8 to 7.1)	3.6 (3.1 to 4.2)	< 0.001
**Overall cohort**	No oral	34.9 (33.5 to	38.3 (37.1 to	40.3 (38.9 to	<

	anticoagulant	36.4)	39.6)	41.7)	0.001
**Phenotype 5 (Warfarin-Predominant Anticoagulation)**	Warfarin only, no DOAC	84.4 (81.7 to 86.7)	81.5 (78.8 to 83.9)	71.5 (67.3 to 75.3)	< 0.001
**Phenotype 5 (Warfarin-Predominant Anticoagulation)**	DOAC only, no warfarin	0.6 (0.3 to 1.5)	2.2 (1.5 to 3.4)	5.7 (4.0 to 8.2)	< 0.001
**Phenotype 5 (Warfarin-Predominant Anticoagulation)**	Both warfarin and a DOAC	9.5 (7.7 to 11.7)	8.3 (6.7 to 10.3)	10.1 (7.7 to 13.1)	0.882
**Phenotype 5 (Warfarin-Predominant Anticoagulation)**	No oral anticoagulant	5.5 (4.1 to 7.3)	8.0 (6.4 to 9.9)	12.7 (10.1 to 16.0)	< 0.001
**Overall cohort, single-encounter patients**	Warfarin, ever	29.5 (26.9 to 32.2)	24.0 (22.4 to 25.6)	12.9 (11.7 to 14.1)	< 0.001
**Overall cohort, single-encounter patients**	DOAC, ever	20.1 (17.9 to 22.6)	30.1 (28.4 to 31.8)	43.1 (41.3 to 44.8)	< 0.001
**Overall cohort, single-encounter patients**	Any oral anticoagulant, ever	48.5 (45.6 to 51.4)	52.1 (50.2 to 54.0)	54.1 (52.3 to 55.8)	0.001
**Overall cohort, single-encounter patients**	No oral anticoagulant	51.5 (48.6 to 54.4)	47.9 (46.0 to 49.8)	45.9 (44.2 to 47.7)	0.001
**Phenotype 5 (Warfarin-**	Warfarin, ever	90.0 (86.0 to	86.2 (83.1 to	78.0 (73.5 to	<

**Predominant Anticoagulation), single-encounter patients**		92.9)	88.8)	82.0)	0.001
**Phenotype 5 (Warfarin-Predominant Anticoagulation), single-encounter patients**	DOAC, ever	2.1 (1.0 to 4.4)	7.0 (5.2 to 9.4)	12.6 (9.6 to 16.4)	< 0.001
**Phenotype 5 (Warfarin-Predominant Anticoagulation), single-encounter patients**	Any oral anticoagulant, ever	90.0 (86.0 to 92.9)	88.7 (85.8 to 91.1)	84.6 (80.5 to 88.0)	0.031
**Phenotype 5 (Warfarin-Predominant Anticoagulation), single-encounter patients**	No oral anticoagulant	10.0 (7.1 to 14.0)	11.3 (8.9 to 14.2)	15.4 (12.0 to 19.5)	0.031
**Phenotype 1 (Cerebrovascular-Predominant)**	Share of the era cohort	17.5 (16.4 to 18.7)	17.1 (16.1 to 18.1)	15.9 (14.8 to 17.0)	0.038
**Phenotype 2 (Hypoalbuminemic-Anemic)**	Share of the era cohort	14.7 (13.7 to 15.8)	17.3 (16.3 to 18.3)	21.2 (20.0 to 22.4)	< 0.001
**Phenotype 3 (Low Comorbidity Burden)**	Share of the era cohort	17.2 (16.1 to 18.4)	21.5 (20.4 to 22.6)	23.7 (22.5 to 25.0)	< 0.001

**Phenotype 4 (High AF Chronicity)**	Share of the era cohort	19.1 (17.9 to 20.3)	14.0 (13.1 to 14.9)	13.2 (12.3 to 14.2)	< 0.001
**Phenotype 5 (Warfarin-Predominant Anticoagulation)**	Share of the era cohort	19.6 (18.4 to 20.9)	16.4 (15.4 to 17.4)	10.9 (10.0 to 11.9)	< 0.001
**Phenotype 6 (Renal Dysfunction)**	Share of the era cohort	11.9 (10.9 to 12.9)	13.8 (12.9 to 14.8)	15.1 (14.1 to 16.2)	< 0.001

Percentages are patient-level with Wilson 95% confidence intervals. Overlapping ever flags are not mutually exclusive, so warfarin and DOAC may sum to more than any oral anticoagulant; the mutually exclusive categories (warfarin only, DOAC only, both, no oral anticoagulant) sum to 100% within each era. Trend P values are two-sided Cochran-Armitage tests for trend across the three ordered anchor year groups using equally spaced era scores. Across the whole cohort 25.80% of warfarin-flagged patients also carry a DOAC flag; within Phenotype 5 (Warfarin-Predominant Anticoagulation) almost the entire DOAC signal is that overlap rather than DOAC-only use. Because these are cumulative ever-exposed flags over the whole record, patients anchored in earlier windows have longer records and more opportunity to accumulate any exposure: mean encounters per patient fall from 3.47 to 2.26 to 1.59 across the three eras and the single-encounter share rises from 27.8% to 50.5% to 68.4% (Kruskal-Wallis $H = 1,758.1$, $p < 0.001$). The modest decline in the any oral anticoagulant row should therefore not be read as reduced anticoagulation. The single-encounter sensitivity analysis, restricted to the 6,927 patients with exactly one admission so that the ever flag reduces to index-encounter exposure, shows the same era direction at different absolute levels. Anchor year group is a deidentified three-year window, not a calendar prescription date. Patients per era (2014 to 2016, 2017 to 2019, 2020 to 2022): Overall cohort 4,072, 5,439, 4,456; Phenotype 1 (Cerebrovascular-Predominant) 714, 929, 707; Phenotype 2 (Hypoalbuminemic-Anemic) 599, 940, 945; Phenotype 3 (Low Comorbidity Burden) 701, 1,168, 1,056;

Phenotype 4 (High AF Chronicity) 776, 759, 589; Phenotype 5 (Warfarin-Predominant Anticoagulation) 799, 892, 487; Phenotype 6 (Renal Dysfunction) 483, 751, 672; Overall cohort, single-encounter patients 1,133, 2,746, 3,048; Phenotype 5 (Warfarin-Predominant Anticoagulation), single-encounter patients 290, 558, 364.
